# Association of capsular types with carbapenem resistance, disease severity, and mortality in *Acinetobacter baumannii*

**DOI:** 10.1080/22221751.2020.1822757

**Published:** 2020-09-24

**Authors:** Yu-Chia Hsieh, Shi-Heng Wang, Yi-Yin Chen, Tzu-Lung Lin, Shian-Sen Shie, Ching-Tai Huang, Chen-Hsiang Lee, Yi-Ching Chen, Tran Lam Tu Quyen, Yi-Jiun Pan

**Affiliations:** a Department of Pediatrics, Chang Gung Children’s Hospital, Chang Gung Memorial Hospital, Chang Gung University, College of Medicine, Taoyuan, Taiwan; b Department of Occupational Safety and Health and Public Health, College of Public Health, China Medical University, Taichung, Taiwan; c Department of Medical Biotechnology and Laboratory Science, Chang Gung University, Taoyuan, Taiwan; d Division of Infectious Diseases, Department of Internal Medicine, Chang Gung Memorial Hospital, Taipei, Taoyuan, Taiwan; e Division of Infectious Diseases, Department of Internal Medicine, Chang Gung Memorial Hospital – Kaohsiung Medical Center, Chang Gung University, Kaohsiung, Taiwan; f Department of Microbiology and Immunology, School of Medicine, College of Medicine, China Medical University, Taichung, Taiwan

**Keywords:** *Acinetobacter baumannii*, capsular type, serotype, typing system, carbapenem resistance

## Abstract

*Acinetobacter baumannii* emerged as one of the most important pathogens that causes nosocomial infections due to its increased multidrug resistance. Identifying capsular epidemiology in *A. baumannii* can aid in the development of effective treatments and preventive measures against this emerging pathogen. Here we established a *wzc*-based method, and combined it with *wzy*-PCR to determine capsular types of *A. baumannii* causing nosocomial bacteraemia collected at two medical centres in Taiwan from 2015 to 2017. Among the 237 patients with *A. baumannii* bacteraemia, 98 (41.4%) isolates were resistant to carbapenems. Four prevalent capsular types (KL2, KL10, KL22, and KL52) accounted for 84.7% of carbapenem-resistant *A. baumannii* (CRAB) and 12.2% of non-CRAB. The rate of pneumonia, intensive care unit admission, APACHE II score, and Pitt bacteraemia score were higher in patients with KL2/10/22/52 infection than in those with non-KL2/10/22/52 infection. Patients with KL2/10/22/52 infection and patients with CRAB infection have a higher cumulative incidence of attributable and all-cause in-hospital 30-day mortality. On multivariate analysis, appropriate empirical antimicrobial therapy within 24 h was associated with a lower risk of 30-day attributable mortality in the KL2/10/22/52 isolates (odds ratio = 0.19, 95% CI: 0.06–0.66, *p* = 0.008) but not in non-KL2/10/22/52 isolates. Early recognition of carbapenem resistance-associated capsular types may help clinicians to promptly implement appropriate antimicrobial therapy for improving the outcomes in patients with CRAB bacteraemia.

## Introduction

*Acinetobacter baumannii* has emerged as a predominant cause of nosocomial infections in the last decade. It poses characteristics of environmental persistence, resistance to dryness, and evasion of host immunity, which makes it a major threat in health-care facilities, particularly among immunocompromised patients [1]. Studies have documented that mortality associated with gram-negative bacteraemia was significantly increased in *A. baumannii* infections compared to other gram-negative bacilli [2,3]. It has emerged as one of the most troublesome gram-negative bacteria worldwide due to its high level of antibiotic resistance, with some exhibiting resistance to most clinically available antibiotics [4]. The spread of multidrug-resistant *A. baumannii* (MDRAB), especially carbapenem-resistant *A. baumannii* (CRAB) in intensive care units (ICU), has been growing globally [5]. More than 50% of *A. baumannii* isolates from US ICU during 2009–2012 were resistant to carbapenem and all other antibiotics except colistin or tigecycline [6]; the rate of CRAB has reached 70.2% in 2018, observed in Taiwan Nosocomial Infection Surveillance (TNIS) system supported by Centers for Disease Control (CDC), Taiwan [7]. Crude mortality for CRAB infections ranged from 16 to 76% [8]. A systemic review suggested that infection with CRAB may be associated with a two-fold increase in the risk of mortality compared to carbapenem susceptible *A. baumannii* [8]. Therefore, the World Health Organization declared CRAB as a top priority pathogen, which desperately needed the development of active antimicrobials [9].

Capsular polysaccharide (CPS) is a critical virulence factor for *A. baumannii* [10]. The layer of CPS makes *A. baumannii* more resistant to external stresses such as complement-mediated killing and certain antibiotics, and contributes to its ability to survive in the hospital environment for long periods [10,11,12]. Functional studies also revealed the importance of CPS during *A. baumannii* infection and growth in serum [10,13]. Capsular serotype-specific antibody against *A. baumannii* increased neutrophil opsonopagocytosis *in vitro* and increased bacterial clearance *in vivo* [14]. It is biologically plausible that capsule of *A. baumannii* is an adequate preventative and therapeutic target for the development of novel control strategies for *A. baumannii* infection. However, available capsular typing methods for *A. baumannii* rely on complete sequences of ∼20 kb *cps* region and thus is difficult for comprehensively serotyping multiple infecting strains.

A *wzc* genotyping method was reported in *Klebsiella pneumoniae* [15], which permits detection of capsular types (K-types) of *K. pneumoniae,* including the ones where *cps* sequences are unavailable; moreover, only one round of sequencing of PCR products is needed, and has been used for identifying K-types of *K. pneumoniae* in different studies [16,17,18]. Here we establish a *wzc*-based method for *A. baumannii*, and combined it with *wzy*-PCR to determine K-types of *A. baumannii* causing nosocomial bacteraemia. To our knowledge, this is the first study to screen K-types in large numbers of clinical isolate of *A. baumannii* without whole genome sequencing, and discovered the prevalent K-types in CRAB.

## Materials and methods

### Study population

**Figure 1:**
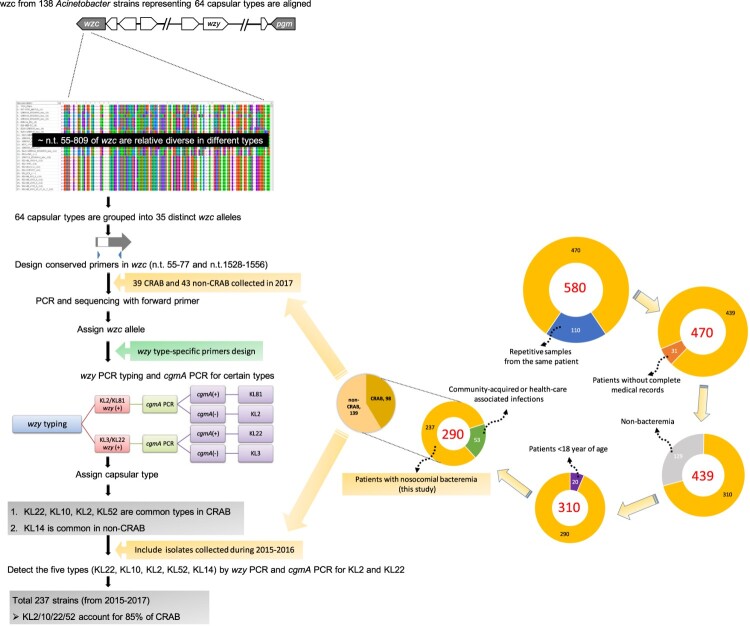
. Flow diagram of capsular typing using *wzc*-based method followed by *wzy*-PCR genotyping and samples collection.

This study was conducted at Chang Gung Memorial Hospital (CGMH)-Lin Kou branch, a 3700-bed medical centre in northern Taiwan, and CGMH-Kaohsiung branch, a 2700-bed medical center in southern Taiwan, from January 2015 to December 2017. Nosocomial *A. baumannii* bacteraemia was defined by the presence of ≥1 positive blood culture results for patients with sign and symptoms of infection, which occurred >48 h after hospital admission. For patients with multiple episodes of bacteraemia, only the first episode was included. Patients<18 years of age or with incomplete medical records were excluded. This protocol was approved by the institutional review board of CGMH (Approval number: 201801433B0). The iterative process of sample collection was described as follows. A total of 580 samples were collected, repetitive samples from the same patient (*n *= 110) were excluded, patients without complete medical records (*n *= 31) were excluded, non-bacteraemia samples (*n *= 129) were excluded, patients <18 year of age (*n *= 20) were excluded, and community-acquired or health-care associated infection (*n *= 53) were excluded. Finally, a total of 237 samples from patients with nosocomial bacteraemia were analysed in this study (Figure 1 and supplementary file).

### Microbiological studies

Identification of *A. baumannii* was performed using a matrix-assisted laser desorption-time of flight mass spectrometry (MALDI-TOF-MS) [19]. Susceptibility to all tested antibiotics except tigecycline [20] was determined according to the Clinical and Laboratory Standards Institute (CLSI) interpretive criteria for the disk diffusion method [21]. Minimum inhibitory concentration (MIC) of imipenem and meropenem was further determined using the broth dilution method [21], respectively. Carbapenem resistance was defined as MICs of >4 μg/ml for both imipenem/ meropenem.

### K-typing

Owing to the successful K-typing of *Klebsiella* on the *wzc* gene [15], and because Wzc is believed to function as a tyrosine kinase, which interacts with the outer membrane protein Wza and phosphatase Wzb to form a trans-envelope capsule translocation complex, it is thus reasonable that Wzc domains might interact with type-specific capsular polysaccharides during the process of translocation. Therefore, we analysed the sequences of *wzc* genes from 138 *Acinetobacter* strains representing 64 K-types (see Supplementary Table 1; 5 non-baumannii strains described in a previous study analysing *Acinetobacter cps* [22] were also included because different *Acinetobacter* spp. may share the same capsule structure). Two nomenclature systems for capsule biosynthesis locus, KL(K) types [23], and PSgc types [22], have been used in different reports. KL type and corresponding PSgc types were given in Supplementary Table 1. Since KL has been widely adopted, we use KL nomenclature in the text unless KL type was unavailable.

The results showed that ∼n.t.55 to ∼n.t.809 of *wzc* genes were relatively diverse in different types but conserved in strains with the same type. The *wzc* sequences are provided in the supplementary file. Most of the strains with the same type showed ≧99% DNA identity with some exceptions. KL2 and Psgc63 showed 97% identity with their same K-type strains and four KL10 strains were clustered into two groups (TYTH_1 and NCGM_237) showed 98% DNA identity, and BAL_030 and XH857 showed 100% DNA identity, and the DNA identity between the two groups is 91% (Supplementary Table 2). On the other hand, most of the strains with different K-types showed <95% DNA identity with the exception of some K-types sharing relative high similarity with each other (Supplementary Table 3). We assigned the sequences given ≧95% DNA identity as an allele. A total of 64 K-types were grouped into 35 distinct alleles (Supplementary Table 3). We further designed conserved primers AB_wzcF1 and AB_wzcR5-plus located at n.t. 55–77 and n.t. 1528–1556, respectively. After *wzc* PCR was conducted, the expected PCR products were subjected to Sanger sequencing with AB_wzcF1. The sequences were compared to the 138 *wzc* sequences in our *wzc* panel (sequences are provided in the supplementary file). *wzy* PCR was further conducted to confirm the K-types with type-specific *wzy* primers (Supplementary Table 4), starting with the corresponding *wzc* type with highest DNA identify. If *wzy* PCR was negative for the most likely type (the type with highest identity), the second possible type would be tested. The iterative PCR process was continued to assign a *wzy* type for the strain. For certain K-types share the same *wzy*, further examination relies on the difference in other *cps* genes is needed to identify the K-types. For example, KL2 and KL81 share a *wzy* gene with three nucleotide differences and thus were further distinguished by *cgmA* PCR (*cgmA* was present in KL81 but not in KL2). KL3 and KL22 share a *wzy* with only one nucleotide difference, and thus were further distinguished by *cgmA* PCR (*cgmA* was present in KL22 but not in KL3).

### Data collection and definitions

Medical records were reviewed to collect clinical information, including demographic characteristics; comorbid conditions, hospital stays; ICU stays; time of antimicrobial therapy; and the presence of a ventilator, central venous catheters, or a foley catheter at the time of bacteraemia onset. Hospital stays were defined from the date of bacteria sample collection to date of discharge. Comorbidity at diagnosis was classified using the Charlson comorbidity index. The severity of illness was assessed using Acute Physiology and Chronic Health Evaluation II (APACHE II) score [24] in ICU patients and the Pitt bacteraemia score [25] in all patients. Immunosuppressive therapy was defined as receipt of cytotoxic agents, or other immunosuppressive agents within 6 weeks, or corticosteroids at a dosage equivalent to or higher than 20 mg of prednisolone daily for 2 weeks or 30 mg of prednisolone daily for at least 1 week before bacteraemia onset. The primary infection source of bacteraemia was determined according to the definitions of the Centers for Disease Control and Prevention [26]. If no infectious focus was identified, the bacteraemia was counted as primary. Polymicrobial bacteraemia was defined as isolation of ≥1 microorganisms other than *A. baumannii* from blood during the same bacteraemic episode. Antimicrobial therapy was considered appropriate if the drugs used at therapeutic doses had *in vitro* activity against the strain isolated after the onset of bacteraemia [27,28](we use “appropriate empirical antimicrobial therapy” in this study). Antimicrobial therapy that did not meet this definition was considered inappropriate. Data on 14-day and 30-day mortality and all-cause in-hospital mortalities were recorded. Attributable mortality (bacteraemia-related death) was defined as death before resolution of symptoms and signs of bacteraemia and at least one blood culture positive for *A. baumannii*.

### Statistical analysis

We examined whether the distribution of demographic characteristics underlying diseases, sources of infection, and clinical characteristics differed in terms of *A. baumannii* K-types. The analysis of variance (ANOVA) tests was performed for continuous variables and Fisher’s exact tests were performed for categorical variables. We used the Kaplan-Meier survival curves to display the cumulative probabilities of all-cause mortality and attributable mortality within 30 days, and used a log-rank test to compare the difference in survival functions between *A. baumannii* K-type and carbapenem resistance.

With adjustment for potential confounders, we used multivariate regression models to estimate the effects of *A. baumannii* K-type for four clinical outcomes; logistic regression models were performed for 30-day attributable mortality, 30-day all-cause mortality, and carbapenem resistance, and linear regression model was performed for Pitts score. We performed different models with varied adjustments to evaluate the influence of *A. baumannii* K-type after adjusting different covariates. To further explore the interactive effects between *A. baumannii* K-type, carbapenem resistance, and appropriate empirical antimicrobial therapy on 30-day attributable mortality, stratified analysis based on *A. baumannii* K-type or carbapenem resistance were carried out. All statistical analyses were conducted with the SAS 9.4. A *p*-value of  < 0.05 was considered statistically significant.

## Results

### Antibiotic susceptibility

A total of 237 non-redundant *A. baumannii* strains causing nosocomial bacteraemia were collected from the Linkou CGMH (156 strains) and Kaohsiung CGMH (81 strains) from 2015 to 2017. Out of these strains, 41% (*n *= 98) were defined as carbapenem-resistant. The meropenem MIC_50_ and MIC_90_ of CRAB were both >32 μg/mL, and the imipenem MIC_50_ and MIC_90_ were 32 μg/mL and >32 μg/mL, respectively. All isolates were susceptible to colistin. The non-susceptibility rates were 41% to amikacin, 49% to ceftazidime, 48% to ciprofloxacin, 47% to cefepime, 49% to gentamicin, 44% to ampicillin-sulbactam, 49% to tazobactam, and 15% to tigecycline.

### K-typing of *A. baumannii*

**Figure 2.**
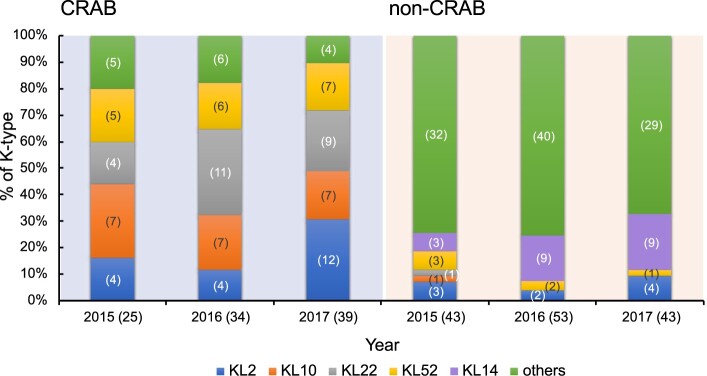
The distribution of *Acinetobacter baumannii* capsular types with or without carbapenem resistance during 2015–2017. Number of isolates were shown in parentheses.

*Wzc* typing were conducted in 39 CRAB and 43 non-CRAB collected in 2017 (Figure 1). Sequences of 39 CRAB strains showed ≧97% DNA identity with 7 different *wzc* alleles and those of the 43 non-CRAB strains showed 87%-100% DNA identity with 16 *wzc* alleles (Supplementary Table 5). Based on *wzc* typing results, K-types of these strains were further determined by *wzy*-PCR with type-specific *wzy* primers. The distribution of KL types in 2017 is shown in (Figure 2). The four types KL2/10/22/52 account for 89.7% CRAB. For 43 non-CRAB, 28 strains were assigned to 12 different K-types; 15 strains were unknown (i.e. *wzy*-PCR was negative for possible *wzc* types and these strains were also confirmed to be non-KL2/10/22/52 type). In contrast to CRAB, type KL2/10/22/52 only account for 11.6% non-CRAB and the most prevalent type is KL14 (20.9%). We then tested strains from 2015to 2016 with *wzy* primers to determine the prevalence of the five types (KL2, KL10, KL14, KL22, and KL52) (Figure 2). In total, the four major KL2/10/22/52 types were predominant in CRAB, but not non-CRAB (85% vs. 12%) during the study period. Notably, the number of KL2 apparently increased in CRAB (from 16% in 2015 to 30.8% in 2017) but not in non-CRAB (7.0% in 2015 and 9.3% in 2017).

### The distribution of demographic and clinical factors by *A. baumannii* capsular type

The demographic characteristics, underlying diseases, sources of infection, and clinical characteristics of patients with *A. baumannii* bacteraemia are shown in Table 1. There were no signiﬁcant differences among patients with different K-types with respect to age, sex, and the Charlson scores. However, patients with the four major types of KL2/10/22/52 bacteraemia had more frequent chronic renal insufficiency and had less metastatic tumour than did patients with non-KL2/10/22/52 bacteraemia. The source of bacteraemia being pneumonia, ICU admission rate, ICU stays, APACHE II score, and Pitt bacteraemia score were significantly higher in the KL2/10/22/52 isolates. Besides, the KL2/10/22/52 isolates showed more carbapenem resistance. There were lower rates of appropriate empirical antimicrobial therapy within 24 h among patients with the KL2/10/22/52 isolates than there were among those with non- KL2/10/22/52 isolates.

**Table 1.** Demographic characteristics, underlying diseases, sources of infection, clinical characteristics of patients with *Acinetobacter baumannii* Bacteraemia.

	KL type					p* for comparing KL 2, 10, 22, 52, and other	p* for comparing total number of KL2/10/22/52 and other
	2 (*n* = 29)	10 (*n *= 22)	22 (*n *= 25)	52 (*n *= 24)	Other (*n *= 137)		
Male, number (%)	19 (65.5)	16 (72.7)	13 (52.0)	16 (66.7)	77 (56.2)	0.46	0.28
Age (years), mean (SD)	68.0 (12.6)	58.5 (13.7)	65.9 (13.1)	60.3 (16.2)	62.8 (13.9)	0.09	0.68
Charlson score, mean (SD)	4.5 (2.8)	5.2 (3.4)	4.3 (2.2)	4.0 (2.2)	5.0 (2.5)	0.31	0.15
Underlying conditions, number (%)							
DM with end organ disease	1 (3.5)	3 (13.6)	5 (20.0)	5 (20.8)	10 (7.3)	0.05	0.13
Liver cirrhosis	8 (27.6)	4 (18.2)	4 (16.0)	3 (12.5)	21 (15.3)	0.57	0.49
Hypertension	12 (41.4)	14 (63.6)	10 (40.0)	11 (45.8)	55 (40.2)	0.35	0.35
Coronary artery disease	1 (3.5)	5 (22.7)	2 (8.0)	1 (4.2)	13 (9.5)	0.21	0.99
Congestive heart failure	0 (0.0)	3 (13.6)	2 (8.0)	5 (20.8)	14 (10.2)	0.1	0.99
Chronic renal insufficiency	4 (13.8)	11 (50.0)	10 (40.0)	11 (45.8)	28 (20.4)	0.001	0.01
Chronic obstructive pulmonary disease	6 (20.7)	2 (9.1)	5 (20.0)	2 (8.3)	11 (8.0)	0.16	0.1
Autoimmune disease	2 (6.9)	1 (4.6)	2 (8.0)	1 (4.2)	2 (1.5)	0.11	0.07
Tumour with metastases	3 (10.3)	6 (27.3)	2 (8.0)	2 (8.3)	49 (35.8)	0.0006	<0.0001
Leukemia	1 (3.5)	1 (4.6)	1 (4.0)	1 (4.2)	9 (6.6)	0.99	0.57
Lymphoma	1 (3.5)	1 (4.6)	0 (0.0)	1 (4.2)	0 (0.0)	0.06	0.07
Solid malignancy	11 (37.9)	9 (40.9)	8 (32.0)	3 (12.5)	61 (44.5)	0.04	0.04
Use of immunosuppressive agent, number (%)	4 (13.8)	9 (40.9)	7 (28.0)	5 (20.8)	43 (31.4)	0.2	0.31
Source of bacteraemia, number (%)	2 (6.9)	1 (4.6)	1 (4.0)	2 (8.3)	19 (13.9)	0.55	0.06
Primary bacteraemia							
Pneumonia	19 (65.5)	13 (59.1)	16 (64.0)	12 (50.0)	37 (27.0)	<0.0001	<0.0001
Ventilator-associated pneumonia	16 (55.2)	10 (45.5)	14 (56.0)	8 (33.3)	7 (5.1)	<0.0001	<0.0001
Central venous catheter (CLABSI)	13 (44.8)	9 (40.9)	3 (12.0)	6 (25.0)	50 (36.5)	0.06	0.41
Intra-abdominal infection	1 (3.5)	1 (4.6)	1 (4.0)	3 (12.5)	19 (13.9)	0.35	0.06
Surgical site infection	3 (10.3)	2 (9.1)	2 (8.0)	1 (4.2)	6 (4.4)	0.5	0.27
Urinary tract infection	3 (10.3)	2 (9.1)	2 (8.0)	2 (8.3)	11 (8.0)	0.99	0.82
Foley’s catheter	2 (6.9)	2 (9.1)	0 (0.0)	1 (4.2)	2 (1.5)	0.09	0.14
Polymicrobial bacteraemia, number (%)	11 (37.9)	6 (27.3)	7 (28.0)	10 (41.7)	56 (40.9)	0.62	0.34
Carbapenem resistant, number (%)	20 (69.0)	21 (95.5)	24 (96.0)	18 (75.0)	15 (11.0)	<0.0001	<0.0001
Appropriate empirical antimicrobial therapy^a^ within 24 h, number (%)	9 (31.0)	7 (31.8)	3 (12.0)	11 (45.8)	83 (60.6)	<0.0001	<0.0001
ICU, number (%)	13 (44.8)	14 (63.6)	12 (48.0)	15 (62.5)	30 (21.9)	<0.0001	<0.0001
ICU stay days, mean (SD)	36.8 (21.4)	42.8 (50.9)	25.0 (17.6)	24.8 (26.2)	16.2 (18.4)	0.04	0.01
APACHE II score (if ICU = y), mean (SD)	25.0 (5.5)	25.0 (6.0)	24.8 (9.1)	24.9 (9.0)	19.6 (8.8)	0.08	0.004
Pitt score, mean (SD)	3.6 (3.6)	4.9 (4.0)	4.8 (3.9)	4.5 (3.5)	2.4 (3.2)	0.0003	<0.0001
Hospital stay days, mean (SD)	22.0 (22.0)	27.3 (36.1)	15.4 (28.3)	25.5 (29.3)	21.2 (25.5)	0.58	0.74
All cause in hospital mortality, number (%)	15 (51.7)	14 (63.6)	22 (88.0)	14 (58.3)	45 (32.9)	<0.0001	<0.0001
Within 14 days	10 (34.5)	8 (36.4)	15 (60.0)	9 (37.5)	31 (22.6)	0.005	0.002
Within 30 days	13 (44.8)	10 (45.5)	21 (84.0)	10 (41.7)	38 (27.7)	<0.0001	<0.0001
Attributable mortality, number (%)	9 (31.0)	10 (45.5)	19 (76.0)	10 (41.7)	33 (24.1)	<0.0001	0.0002
Within 14 days	8 (27.6)	9 (40.9)	14 (56.0)	8 (33.3)	30 (21.9)	0.009	0.006
Within 30 days	9 (31.0)	10 (45.5)	18 (72.0)	8 (33.3)	33 (24.1)	0.0001	0.0008

Note: DM: diabetes mellitus, ICU: intensive care unit, APACHE: acute physiology and chronic health evaluation, CLABSI, central line associated blood stream infection.

^a^ appropriate empirical antimicrobial therapy within 24 h was defined by in vitro susceptibility test.

*ANOVA test or Fisher exact test.

**Figure 3.**
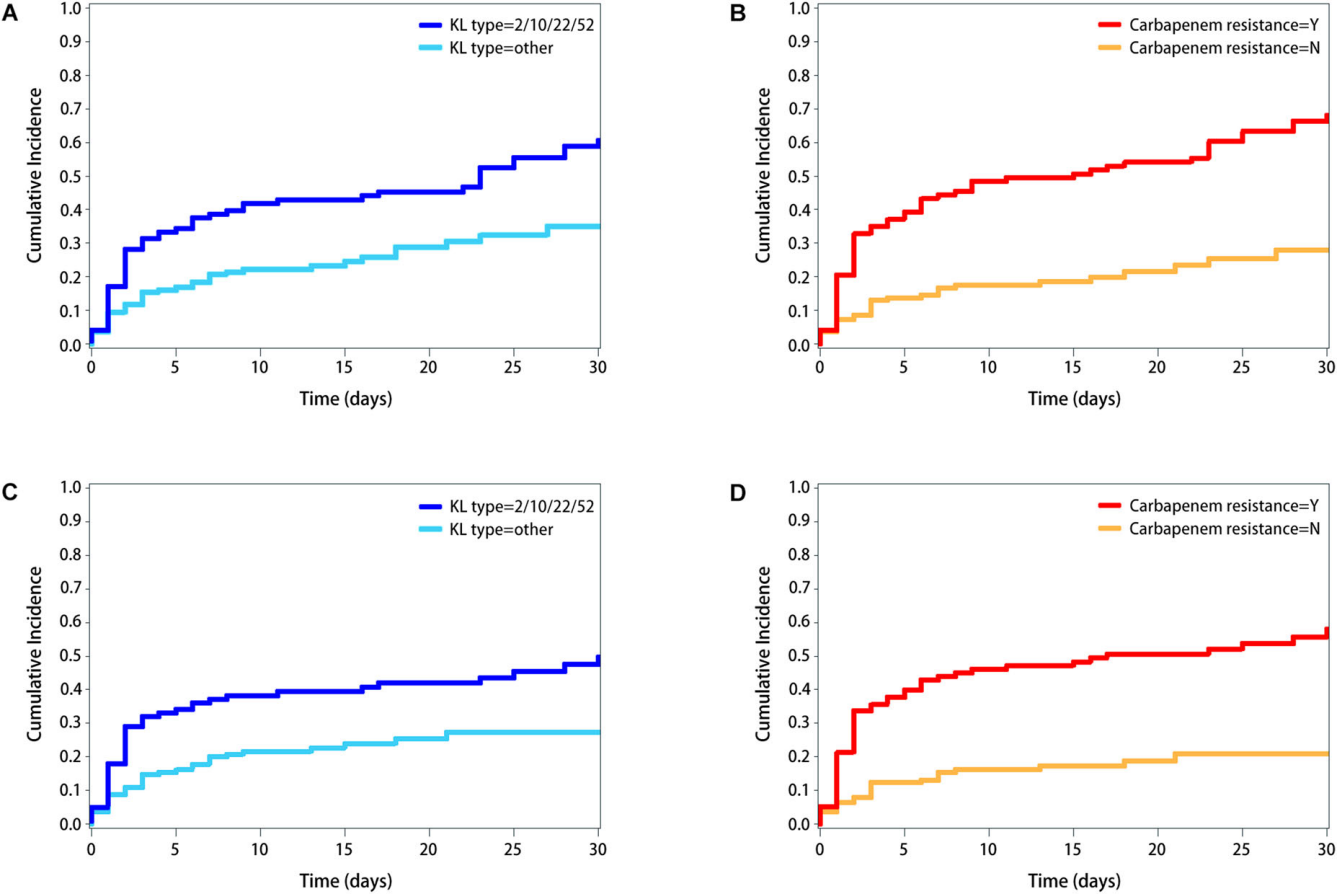
Time to occurrence of all-cause mortality within 30 days in (A) Four major K-type KL2/10/22/52 v.s. other types (B) Carbapenem resistance, Yes v.s. No. Time to occurrence of attributable mortality within 30 days in (C) Four major K-type KL2/10/22/52 v.s. other types (D) Carbapenem resistance, Yes v.s. No.

Both attributable and all-cause in-hospital mortality were higher for those with the KL2/10/22/52 isolates. Kaplen–Meier survival curves were shown in Figure 3. The patients infected with the major four types of KL2/10/22/52 and patients with carbapenem resistance had a higher cumulative incidence of attributable and all-cause in-hospital mortality in 30 days (all *p* < 0.001 for log-rank tests).

### Association between K-type and outcomes

With adjustment for sex, age, charlson score, and ICU, the patients infected with the KL2/10/22/52 isolates had a higher risk of 30-days attributable and all-cause mortality and a higher Pitt score (Table 2). However, the risk effect disappeared after further adjusting pneumonia, appropriate empirical antimicrobial therapy, and carbapenem resistance. The KL2/10/22/52 isolates were strongly associated with carbapenem resistance regardless of adjustments.

**Table 2.** Association between capsular type and outcomes.

	30-days Attributable Mortality		30-days all-cause Mortality		Carbapenem resistance		Pitt score	
Model: adjustment	OR (95% CI)*	*p*	OR (95% CI)*	*p*	OR (95% CI)*	*p*	beta (95% CI)^#^	*p*
M0: Crude model	2.58 (1.48–4.49)	0.0008	3.06 (1.78–5.26)	<0.0001	39.71 (18.79–83.92)	<0.0001	2.01 (1.12, 2.90)	<0.0001
M1: sex, age, Charlson score, ICU	2.26 (1.25–4.09)	0.007	2.60 (1.46–4.65)	0.001	38.93 (16.90–89.70)	<0.0001	1.62 (0.68, 2.57)	0.0008
M2: M1, Pneumonia	1.60 (0.84–3.04)	0.15	1.88 (1.01–3.51)	0.05	35.93 (14.90–86.68)	<0.0001	0.97 (0.03, 1.91)	0.04
M3: M2, Appropriate empirical antimicrobial therapy ^a^ within 24 h	1.30 (0.66–2.56)	0.45	1.80 (0.93–3.48)	0.08	28.26 (11.54–69.21)	<0.0001	0.92 (−0.07, 1.92)	0.07
M4: M3, Carbapenem resistance	0.47 (0.17–1.24)	0.13	0.64 (0.25–1.63)	0.35			0.26 (−0.97, 1.50)	0.68

Note: ICU: intensive care unit.

^a^ appropriate empirical antimicrobial therapy within 24 h was defined by in vitro susceptibility test.

*estimated from logistic regression model; ^#^estimated from linear regression model. Severity of illness was assessed using Pitt score [25].

In the stratified analysis by K-types (Table 3), appropriate empirical antimicrobial therapy was associated with a lower risk of 30-days attributable mortality in the KL2/10/22/52 isolates (OR = 0.19, 95% confidence interval 0.06–0.66, *p *= 0.008), whereas such association was lacking in other types. The difference in the effect of appropriate empirical antimicrobial therapy by K-type reached statistical significance (p for interaction between appropriate empirical antimicrobial therapy and K-type = 0.02). In both KL2/10/22/52 and non-KL2/10/22/52 types, carbapenem resistance was the strongest risk factor for 30-days attributable mortality. In the stratified analysis by carbapenem resistance (Table 4), K-type was not associated with 30-days attributable mortality. Appropriate empirical antimicrobial therapy was associated with a lower risk of 30-days attributable mortality in patients with carbapenem resistance (OR = 0.23, 95% confidence interval 0.07–0.77, *p *= 0.02), whereas such association lacked in patients without carbapenem resistance, though such difference did not reach statistical significance.

**Table 3.** Association between carbapenem resistance, appropriate antibiotic treatment within 24 h, and 30-days attributable mortality, stratified by capsular types.

	Non-2/10/22/52 (*n *= 137)		2/10/22/52 (*n *= 100)		*P* of interaction
	OR (95% CI)*	*p*-value	OR (95% CI)*	*p*-value	
Sex	1.47 (0.54–4.01)	0.45	0.75 (0.25–2.32)	0.62	0.43
Age	1.03 (0.99–1.07)	0.18	0.99 (0.95–1.04)	0.79	0.58
Charlson score	1.10 (0.89–1.35)	0.39	1.13 (0.90–1.42)	0.30	0.92
Pneumonia	1.64 (0.58–4.61)	0.35	3.89 (1.20–12.63)	0.02	0.37
Pitt score	1.43 (1.21–1.69)	<0.0001	1.40 (1.15–1.70)	0.0007	0.70
Appropriate empirical antimicrobial therapy ^a^ within 24 h	1.50 (0.51–4.39)	0.46	0.19 (0.06–0.66)	0.008	0.02
Carbapenem resistance	3.86 (0.88–16.93)	0.07	7.90 (1.25–49.82)	0.03	0.33

^a^ appropriate empirical antimicrobial therapy within 24 h was defined by in vitro susceptibility test.

*estimated from logistic regression model.

**Table 4.** Association between capsular type, appropriate antibiotic treatment within 24 h, and 30-days attributable mortality, stratified by carbapenem resistance.

	Carbapenem resistance: N (*n *= 139)		Carbapenem resistance: Y (*n *= 98)		*P* of interaction
	OR (95% CI)*	*p*-value	OR (95% CI)*	*p*-value	
KL2/10/22/52 v.s. non-KL2/10/22/52	0.24 (0.04–1.47)	0.13	0.59 (0.13–2.64)	0.49	0.33
Sex	1.81 (0.64–5.08)	0.26	0.76 (0.26–2.21)	0.61	0.29
Age	1.03 (0.99–1.08)	0.12	0.99 (0.95–1.03)	0.57	0.20
Charlson score	1.08 (0.88–1.32)	0.46	1.09 (0.87–1.37)	0.44	0.99
Pneumonia	2.26 (0.81–6.35)	0.12	1.51 (0.50–4.52)	0.46	0.95
Pitt score	1.31 (1.13–1.52)	0.0004	1.56 (1.25–1.96)	0.0001	0.17
Appropriate empirical antimicrobial therapy ^a^ within 24 h	1.05 (0.37–2.97)	0.93	0.23 (0.07–0.77)	0.02	0.22

^a^ appropriate empirical antimicrobial therapy within 24 h was defined by in vitro susceptibility test.

*estimated from logistic regression model.

## Discussion

Biosynthesis of capsular polysaccharides in *Acinetobacter* is similar to *Escherichia coli* K30, as a model system of *E. coli* group 1 capsule assembly involved in *wza*/*wzb*/*wzc* export pathway [29,30]. The process is initiated by initial glycosyltransferase, which transfers a sugar precursor to inner membrane lipid carrier, and is followed by joining additional sugars catalysed by other specific glycosyltransferases [30]. The lipid-linked repeat units are translocated across the inner membrane into the periplasm by Wzx flippase and then polymerized into a chain by Wzy polymerase [30,31]. In general, genes required for capsule biosynthesis are clustered and variation in these loci leads to structural heterogeneity in K antigens. Previous reports described the difference of *A. baumannii* strains in capsule biosynthesis locus/polysaccharide gene cluster using two nomenclature systems, KL(K) types [23] and PSgc types [22], respectively. KL refers to K (capsule) locus, whereas PSgc refers to polysaccharide gene clusters. Kenyon et al. identified nine distinct capsule biosynthesis loci, designated KL1-KL9 [23]. Hu et al. analysed the polysaccharide gene clusters from 190 *Acinetobacter* genome sequences, and identified 25 pre-existing gene clusters [designated PSgc1-PSgc27 (PSgc7 and PSgc16 are identical to PSgc9 and PSgc23, respectively)] [22] and additional 52 new gene clusters. Both studies indicated the conservation of genetic organization, i.e. the gene clusters were between *fkpA* and *lldP* genes, with *wza*, *wzb*, and *wzc* export genes on the left, followed by a highly variable region (*wzx*, *wzy*, and glycosyltranferase genes), and several genes for synthesis of common sugar precursors. It was proposed that a *cps* locus with specific genetic contents represents a specific K-type. A recent study has provided a useful tool – *Kaptive* to compare the whole *cps* region with known K-types of *A. baumannii* when full sequences of *cps locus* is available [32]. It’s a reliable method if recombination occurs in *cps* region, however, amplification of the whole *cps* region or high cost of sequencing an entire genome is challenging for large numbers of clinical isolates. Therefore, for the rapid detection of K-type, an easier alternative strategy could be applied. One method based on *wzy* has been documented [22]. Hu et al. proposed that *wzy*, which is highly variable in sequence between distinct types, would be a candidate gene for a PCR-based molecular serotyping scheme. Despite the fact that *wzy* PCR is a reliable method, it’s difficult to conduct *wzy* typing for large numbers of clinical strains because a separate pair of primers is needed for each type (>106 K-types exist in *A. baumannii*). The *wzc* typing method reported here provides an alternative way to determine possible K-types for *A. baumannii.* According to our results, *wzc* sequencing can be used for prediction of possible K-types and then *wzy* PCR can be performed to confirm the type. It should be noted that even *wzy* PCR is not a gold standard for K-typing because it has been known that two distinct K-types may share the same *wzy* which may be due to recombination events in *cps* region. In these cases, further examination relies on the difference in other *cps* genes is needed (eg. *cgmA* PCR for KL2/KL81). However, if complete *cps* region or whole genome sequences were unavailable, *wzc*-*wzy* typing systems would be helpful for K-type analysis. To date, *wzc* typing has been used in two bacteria, *K. pneumoniae* and *A. baumannii*. Compared to *wzc* typing system in *K. pneumoniae* which needs four primer pairs to amplify *wzc* regions, only one primer pair for *A. baumannii* we designed in this current study can successfully amplify all 82 tested strains. However, *wzc* typing seems more discriminative in *K. pneumoniae* K-typing than in *A. baumannii*. Thus, combined with *wzy* genotyping would be strongly suggested in *A. baumannii*.

Previous studies conducted whole genome sequence analysis for MDRAB and KL1 and KL2 seem to be common K-types in global clone (GC) 1 and GC2, respectively [33,34]. A recent study analysed 134 ST1 of GC1 and 2016 ST2 of GC2 *A. baumannii* genome from NCBI and identified the most common K-types were KL1(31.3%) and KL4(18.7%) for ST1 and KL2(32.2%) and KL22(14.4%) for ST2 [32]. Despite that the analysis is not exclusively for MDRAB, KL1 and KL2 were predominant in these strains. In our current study, 4 major K-types were predominant in CRAB with a prevalence rate of KL2(20.4%), KL10(21.4%), KL22(24.5%), and KL52(18.4%) and account for a total of 85% for CRAB. Interestingly, no or rare studies report on KL10 and KL52. Therefore, we speculated that there could be new clones emerging in Taiwan. To clarify this notion, more strains from different hospitals should be included and the characteristics including K-types, virulence genes, and drug resistance genes should be further examined.

Among patients with KL2/10/22/52 infections, coherent with other studies, Pittsburgh bacteraemia scores and inappropriate empirical antimicrobial therapy were risk factors for mortality of *A. baumannii* bacteraemia [35]. Although KL2/10/22/52 were signiﬁcantly more often associated with pneumonia, severe infection, carbapenem resistance, and high mortality, appropriate empirical antimicrobial therapy within 24 h was associated with higher reduction in mortality caused by KL2/10/22/52 infection. In this current study, we evaluate whether the patients received appropriate empirical antimicrobial therapy within 24 h by in vitro drug susceptibility test (please see the definition in the methods section). It should be noted that some drugs may show activity in vitro but still does not cause an effective cure in vivo, however, our results provided evidence on effectiveness which indicated that appropriate empirical antimicrobial therapy within 24 h decreases mortality for KL2/10/22/52 infection. Evidence to date has shown that early institution of appropriate antimicrobial therapy can improve the survival of patients with CRAB infection [35,36]. More than 50% of patients with CRAB infection received discordant antimicrobial therapy after illness, which led to death rates as high as 60–70% [35,36]. Therefore, recognition of KL2/10/22/52 types as soon as possible, especially in patients with high Pittsburgh bacteraemia scores and pneumonia, assist clinicians to promptly implement effective antimicrobial therapy for improving the outcomes in patients with KL2/10/22/52 bacteraemia before the result of in vitro susceptibility test. Generally, in vitro antimicrobial susceptibility test takes >12 h to determine the results. Thus, we suggested that for clinical application in patients with *A. baumannii* infection, *wzy* PCR for four common K-types (KL2, KL10, KL22, KL52) which is associated with carbapenem resistance can be performed (time spent <2 h). Even though an additional primer pair will be needed for distinguishing KL3/KL22 and KL2/KL81, it’s not necessary to perform the second PCR for them because KL3 and KL81 are rare compared to KL22 and KL2. If the strain is positive for common K-type, colistin could be considered for treatment. The procedure seems more tedious than in vitro antibiotics susceptibility test, however, it would be a time-saving method. Notably, despite that K-typing can be considered a rapid strategy for CRAB detection because of the strong association between K-type and carbapenem resistance, a combination of K-typing and in vitro antibiotics susceptibility test will be suggested (former for early detection, latter for confirmation). On the other hand, if researchers work on *A. baumannii* with no antibiotic assessment data, *wzc* typing could be conducted for initial K-type classification (time spent ∼1 day for PCR and sequencing), after that, *wzy* and other specific *cps* genes PCR will be further performed for K-type determination (time spent <2 h).

KL2/10/22/52 may frequently colonize in the respiratory tract and be exposed to antibiotic drugs, therefore may have been potentially submitted to resistance selective pressure, turning into pneumonia-causing CRAB strains. Unexpectedly, we found that appropriate empirical antimicrobial therapy within 24 h did not reduce mortality in patients with non- KL2/10/22/52 infections. The result may indicate that not all strains of *A. baumannii* are equally virulent, there is the propensity for mortality to be associated with the structure of a specific polysaccharide capsule or other capsule-related bacterial factors in certain types of *A. baumannii*.

Polysaccharide capsule, which shields *A. baumannii* from the host immune system, is critical to *A. baumannii* survival during interactions with host immunity. Nevertheless, not all capsule types appear to be similarly effective in shielding. Our study suggested that the disease pattern, antibiotic resistance, and outcome vary with capsule types. There may be variability in the uptake of exogenous DNA (natural competence) [37], transmission, and invasiveness of a given K-type in *A. baumannii*. K-type identification is important for understanding the virulence characterization and clinical manifestation of *A. baumannii* infection. In the future, an investigation into the K-type in *A. baumannii* serve as a target for medical treatment with phage and immune sera, rapid prediction of resistance, preventive measures such as vaccination, and novel strategies for intervention against this emerging pathogen.

## Supplementary Material

Supplemental_file_5_tables_R1_with_RECORD_checklist.docx
