# Supplementary material for: Association of capsular types with carbapenem resistance, disease severity, and mortality in Acinetobacter baumannii
Source: Emerg Microbes Infect. 2020 Sep 24;9(1):2094–104. doi: 10.1080/22221751.2020.1822757 (PMC7534287; doi:10.1080/22221751.2020.1822757)
Supplement: Supplemental_file_5_tables_R1_with_RECORD_checklist.docx [file TEMI_A_1822757_SM2693.docx]

Supplementary Table 1. *Acinetobacter* strains included in *wzc* database

| PSgc | KL | Strain no. | Strain | Species | Accession no. |
| --- | --- | --- | --- | --- | --- |
| 1 | 7 | 2 | 7804 | *baumannii* | CP022283.1 |
|  |  |  | TCDC_AB0715 | *baumannii* | CP002522 |
| 3 | 82 | 1 | LUH5534 | *baumannii* | KC526908 |
| 4 | 116 | 1 | LUH5536 | *nosocomialis* | KC526912 |
| 5 | 9 | 3 | LUH3484 | *baumannii* | KC526895 |
|  |  |  | RUH134 | *baumannii* | CP032055.1 |
|  |  |  | MDR-TJ | *baumannii* | CP003500 |
| 6 | 35 | 1 | LUH5535 | *baumannii* | KC526896 |
| 8 | 83 | 2 | LUH5538 | *baumannii* | KC526898 |
|  |  |  | ABOB15 | *baumannii* | LLJE01000049 |
| 9 | 22 | 4 | LUH5539 | *baumannii* | KC526915 |
|  | 22 |  | LUH5537 | *baumannii* | KC526920 |
|  | 3 |  | ATCC_17978 | *baumannii* | CP000521 |
| 10 | 84 | 1 | LUH5540 | *baumannii* | KC526902 |
| 11 | 14 | 2 | LUH5541 | *nosocomialis* | KC526906 |
|  |  |  | D46 | *baumannii* | KF030679.2 |
| 12 | 2 | 22 | AB_1582-8 | *baumannii* | AMHB00000000 |
|  |  |  | 3990 | *baumannii* | AEOY00000000 |
|  |  |  | Ab11111 | *baumannii* | AKAQ00000000 |
|  |  |  | OIFC087 | *baumannii* | AMFS00000000 |
|  |  |  | A74 | *baumannii* | KJ459911.1 |
|  |  |  | AB_515_8 | *baumannii* | AMHU00000000 |
|  |  |  | AB_1583_8 | *baumannii* | AMHC00000000 |
|  |  |  | AB_1595_8 | *baumannii* | AMHE00000000 |
|  |  |  | AB_1766_8 | *baumannii* | AMJO00000000 |
|  |  |  | AB_2008_23_07_01_7 | *baumannii* | AMHR00000000 |
|  |  |  | AB_2009_04_02_7 | *baumannii* | AMHT00000000 |
|  |  |  | AB_908_12 | *baumannii* | AMHV00000000 |
|  |  |  | AB_909_05 | *baumannii* | AMIA00000000 |
|  |  |  | AB_909_14_7 | *baumannii* | AMIB00000000 |
|  |  |  | AB_TG2026 | *baumannii* | AMIH00000000 |
|  |  |  | AB_TG2631 | *baumannii* | AMIM00000000 |
|  |  |  | AB_TG27323 | *baumannii* | AMIN00000000 |
|  |  |  | AB_TG27327 | *baumannii* | AMIO00000000 |
|  |  |  | AB_TG27331 | *baumannii* | AMIP00000000 |
|  |  |  | Naval_113 | *baumannii* | AMZU00000000 |
|  |  |  | ACICU | *baumannii* | CP000863 |
|  |  |  | OIFC189 | *baumannii* | AFDM00000000 |
|  | 81 | 1 | LUH3713 | *baumannii* | KC526916 |
| 13 | 1 | 4 | LUH5542 | *baumannii* | KC526901 |
|  |  |  | AB307-0294 | *baumannii* | CP001172 |
|  |  |  | AYE | *baumannii* | CU459141 |
|  |  |  | A1 | *baumannii* | CP010781.1 |
| 14 | 85 | 1 | LUH5543 | *baumannii* | KC526913 |
| 15 | 43 | 2 | LUH5544 | *baumannii* | KC526905 |
| 17 | 11 | 9 | LUH5545 | *baumannii* | KC526904 |
|  |  |  | AB900 | *baumannii* | ABXK00000000 |
|  |  |  | J9 | *baumannii* | KF002790.2 |
|  |  |  | OIFC111 | *baumannii* | AMFY00000000 |
|  |  |  | 3.5D | *baumannii* | NZ_MABZ00000000.1 |
|  |  |  | 3207 | *baumannii* | CP015364.1 |
|  |  |  | 1293320 | *baumannii* | NZ_JFEE00000000.1 |
|  |  |  | 1461963 | *baumannii* | NZ_JEWQ00000000.1 |
|  |  |  | ABBL071 | *baumannii* | NZ_LLGB00000000.1 |
|  | 29 | 4 | AB_2007_16_25_01_7 | *baumannii* | AMHI00000000 |
|  |  |  | AB_2007_16_27_01 | *baumannii* | AMHJ00000000 |
|  |  |  | AB_TG27339 | *baumannii* | AMIR00000000 |
|  |  |  | ABBL038 | *baumannii* | NZ_LLDS00000000.1 |
| 18 | 52 | 1 | LUH5546 | *baumannii* | KC526899 |
| 19 | 87a | 1 | LUH5547 | *baumannii* | KC526918 |
| 20 | 88 | 1 | LUH5548 | *baumannii* | KC526910 |
| 21 | 32 | 1 | BAL_058 | *baumannii* | KT359615.1 |
| 22 | 42 | 1 | LUH5550 | *baumannii* | KC526903 |
| 23 | 80 | 2 | LUH3714 | *baumannii* | KC526911 |
|  |  |  | LUH3712 | *baumannii* | KC526914 |
| 24 | 63 | 1 | BAL_103 | *baumannii* | KX712117.2 |
| 25 | 89 | 1 | LUH5552 | *baumannii* | KC526919 |
| 26 | 90 | 1 | LUH5553 | *baumannii* | KC526917 |
| 27 | 15 | 1 | LUH5554 | *baumannii* | KC526900 |
| 39 | 4 | 2 | AB0057 | *baumannii* | CP001182 |
|  |  |  | D81 | *baumannii* | NZ_FBXC00000000.1 |
| 40 | 6/6a | 6 | AB_908-14-7 | *baumannii* | AMHX00000000 |
|  |  |  | AB_2008-15-45 | *baumannii* | AMHL00000000 |
|  |  |  | 1656_2 | *baumannii* | CP001921 |
|  |  |  | AB_2008_15_70 | *baumannii* | AMHO00000000 |
|  |  |  | ABNIH3 | *baumannii* | AFTB00000000 |
|  |  |  | RBH4 | *baumannii* | KF130871 |
| 41 | 19 | 3 | AB_1594-8 | *baumannii* | AMHD00000000 |
|  |  |  | AB_1536_8 | *baumannii* | AMHA00000000 |
|  |  |  | AB_2009_04_01_7 | *baumannii* | AMHS00000000 |
| 43 | 25 | 1 | AB5075 | *baumannii* | AHAH00000000 |
| 44 | 13 | 6 | Ab689 | *baumannii* | MF522810.1 |
|  |  |  | OIFC180 | *baumannii* | AMDQ00000000 |
|  |  |  | AB_2008_15_34_7 | *baumannii* | AMHK00000000 |
|  |  |  | 6014059 | *baumannii* | ACYS02000025 |
|  |  |  | Naval_17 | *baumannii* | AFDO00000000 |
|  |  |  | UMB001 | *baumannii* | AEPK00000000 |
| 46 | 5 | 1 | SDF | *baumannii* | CU468230 |
| 51 | 26 | 1 | BZICU-2 | *baumannii* | ALOH00000000 |
| 52 | 8 | 1 | BAL_097 | *baumannii* | KX712116.2 |
| 53 | 36 | 1 | Naval-72 | *baumannii* | AMFI00000000 |
| 54 | 23 | 2 | OIFC143 | *baumannii* | AFDL00000000 |
|  |  |  | WC-A-92 | *baumannii* | NZ_AMFU00000000.1 |
| 55 | 31 | 1 | OIFC0162 | *baumannii* | AMFH00000000 |
| 56 | 10 | 4 | TYTH_1 | *baumannii* | CP003856 |
|  |  |  | BAL_030 | *baumannii* | KY434633.1 |
|  |  |  | XH857 | *baumannii* | CP014540.1 |
|  |  |  | NCGM_237 | *baumannii* | AP013357.1 |
| 57 | 33 | 1 | WC_141 | *baumannii* | AMSS00000000 |
| 62 | N.A. | 1 | ADP1 | *baylyi* | CR543861 |
| 63 | N.A. | 2 | DR1 | *oleivorans* | CP002080 |
|  |  |  | PHEA-2 | *calcoaceticus* | CP002177 |
| N.A. | 12 | 3 | 6013113 | *baumannii* | ACYR02000042 |
|  |  |  | 6013150 | *baumannii* | ACYQ02000047 |
|  |  |  | D36 | *baumannii* | JN107991.2 |
| N.A. | 24 | 8 | 1043794 | *baumannii* | JEYX01000013 |
|  |  |  | BAL255 | *baumannii* | CZWB01000054 |
|  |  |  | 232184 | *baumannii* | JEYI01000009 |
|  |  |  | 268680 | *baumannii* | JEYN01000012 |
|  |  |  | 655378 | *baumannii* | NZ_JFCE00000000.2 |
|  |  |  | FDAARGOS_123 | *baumannii* | LORJ01000003 |
|  |  |  | M2 | *baumannii* | LAKP01000004 |
|  |  |  | UMB002 | *baumannii* | AEPL01000017 |
| N.A. | 27 | 1 | 4190 | *baumannii* | KT266827.1 |
| N.A. | 28 | 2 | Ab908 | *baumannii* | MF522807.1 |
|  |  |  | OIFC035 | *baumannii* | AMTB01000027 |
| N.A. | 30 | 1 | NIPH190 | *baumannii* | MN166189.1 |
| N.A. | 37 | 1 | NIPH146 | *baumannii* | APOU01000009 |
| N.A. | 39 | 1 | AB_2008-15-71 | *baumannii* | AMHP01000019.1 |
| N.A. | 44 | 1 | NIPH_70 | *baumannii* | MN148385.1 |
| N.A. | 45 | 1 | NIPH201 | *baumannii* | MN166190.1 |
| N.A. | 47 | 2 | NIPH601 | *baumannii* | APQZ01000009 |
|  |  |  | UV1043 | *baumannii* | KX661320.1 |
| N.A. | 48 | 1 | NIPH615 | *baumannii* | MN166191.1 |
| N.A. | 49 | 4 | NIPH1734 | *baumannii* | NZ_KB849325 |
|  |  |  | BAL_173 | *baumannii* | KT359616.1 |
|  |  |  | NIPH335 | *baumannii* | NZ_KB849886 |
|  |  |  | LAC4 | *baumannii* | JICJ01000028 |
| N.A. | 57 | 1 | BAL_212 | *baumannii* | KY434631.1 |
| N.A. | 58 | 1 | BAL_114 | *baumannii* | KT359617.1 |
| N.A. | 73 | 1 | SGH0703 | *baumannii* | MF362178.1 |
| N.A. | 91 | 1 | 1053 | *baumannii* | KM402814.1 |
| N.A. | 93 | 1 | B11911 | *baumannii* | BK010902 |
| N.A. | 102 | 1 | KZ-1102 | *baumannii* | MK399429 |
| N.A. | 105 | 1 | 625974 | *baumannii* | JEXD01000015.1 |
| N.A. | 106 | 2 | 219_ABAU | *baumannii* | JVPN01000008.1 |
|  |  |  | TG22198 | *baumannii* | NZ_ASFT01000013 |
| N.A. | 107 | 1 | MSHR_183 | *baumannii* | MK370022.1 |

KL, K (capsule) locus. PSgc, polysaccharide gene clusters. N.A., not available

| PSgc | KL | Strain no. | DNA identity | Note |
| --- | --- | --- | --- | --- |
| 1 | 7 | 2 | 100 |  |
| 5 | 9 | 3 | 100 |  |
| 8 | 83 | 2 | 100 |  |
| 9 | 22 | 2 | 100 |  |
| 11 | 14 | 2 | 100 |  |
| 12 | 2 | 22 | 97-100 | AB_1583_8 showed 97% identity to others |
| 13 | 1 | 4 | 100 |  |
| 17 | 11 | 9 | 100 |  |
|  | 29 | 4 | 100 |  |
| 23 | 80 | 2 | 100 |  |
| 39 | 4 | 2 | 100 |  |
| 40 | 6/6a | 6 | 99-100 |  |
| 41 | 19 | 3 | 100 |  |
| 44 | 13 | 6 | 100 |  |
| 54 | 23 | 2 | 99-100 |  |
| 56 | 10 | 4 | 91-100 | G1^a^:TYTH_1 and NCGM_237; G2^a^:BAL_030 and XH857 |
| 63 | N.A. | 2 | 97 |  |
| N.A. | 28 | 2 | 100 |  |
| N.A. | 12 | 3 | 100 |  |
| N.A. | 24 | 8 | 100 |  |
| N.A. | 47 | 2 | 100 |  |
| N.A. | 49 | 4 | 100 |  |
| N.A. | 106 | 2 | 100 |  |

Supplementary Table 2. DNA identity of the strains with the same types

KL, K (capsule) locus. PSgc, polysaccharide gene clusters. N.A., not available

a, the *wzc* sequences of KL10 strains were clustered into 2 groups, G1 and G2. (TYTH_1 and NCGM_237 showed 98% DNA identity, and BAL_030 and XH857 showed 100% DNA identity, and the DNA identity between the two groups is 91%)

Supplementary Table 3. *wzc* alleles and K-types

| *wzc* allele | K-type | In-group diversity (DNA identity between different types of the same allele) |
| --- | --- | --- |
| wzc1 | KL7, KL28, KL90 | 99%-100% |
| wzc2 | KL9, KL82 | 97% |
| wzc3 | KL10_G2^a^, KL14, KL43, KL52, KL37, KL48, KL116 | 97%-100% |
| wzc4 | KL35 |  |
| wzc5 | KL26, KL83, KL105 | 100% |
| wzc6 | KL3, KL22 | 100% |
| wzc7 | KL84 |  |
| wzc8 | KL2, KL42, KL58, KL81, KL93 | 97%-100% |
| wzc9 | KL1, KL107 | 99% |
| wzc10 | KL85 |  |
| wzc11 | KL11, KL29 | 100% |
| wzc12 | KL87a |  |
| wzc13 | KL45, KL47, KL88, KL102 | 95%-99% |
| wzc14 | KL32 |  |
| wzc15 | KL10_G1^a^, KL80 | 98-100% |
| wzc16 | KL63 |  |
| wzc17 | KL30, KL89 | 95% |
| wzc18 | KL15 |  |
| wzc19 | KL4 |  |
| wzc20 | KL6/6a, KL23, KL31 | 98-100% |
| wzc21 | KL19, KL39 | 100% |
| wzc22 | KL25, KL91 | 97% |
| wzc23 | KL12, KL13, KL73 | 100% |
| wzc24 | KL5 |  |
| wzc25 | KL8 |  |
| wzc26 | KL36 |  |
| wzc27 | KL33 |  |
| wzc28 | P62^b^ |  |
| wzc29 | P63^b^ |  |
| wzc30 | KL24 |  |
| wzc31 | KL27 |  |
| wzc32 | KL44 |  |
| wzc33 | KL49 |  |
| wzc34 | KL57 |  |
| wzc35 | KL106 |  |

a, the *wzc* seqeunces of KL10 strains were clustered into 2 groups, G1 and G2. (TYTH_1 and NCGM_237 showed 98% DNA identity, and BAL_030 and XH857 showed 100% DNA identity, and the DNA identity between the two groups is 91%)

b, P indicates PSgc type because the corresponding KL type is not available

Supplementary Table 4. Primers used in this study

| **Primer** | **Sequences** | **Purpose** |
| --- | --- | --- |
| AB_wzcF1 | TTTTCACTGATTGCTCAGTGGAA | *wzc* sequencing |
| AB_wzcR5-plus | CTACGTAAGCTCTCAATGGCAATATCATC | *wzc* sequencing |
| Psgc 9_wzy_F | GGAGTAGAGATTGGTTGGG | *wzy* PCR |
| Psgc 9_wzy_R | GCCAACACTTTCAGCATAATC | *wzy* PCR |
| PSgc 11_wzy_F | TGGTGCTCAACGTATAGCAAG | *wzy* PCR |
| PSgc 11_wzy_R | AGACCACCCTAAACCAAACC | *wzy* PCR |
| Psgc 12_wzy_F | TTTTCTCCTGTTTGATGGGG | *wzy* PCR |
| Psgc 12_wzy_R | AAATCAGCATTCCAGCGCAC | *wzy* PCR |
| PSgc 18_wzy_F | ACAGGGTTTTGCTGTTGCAG | *wzy* PCR |
| PSgc 18_wzy_R | ACCTAAGCGAAAACCTAACC | *wzy* PCR |
| Psgc 56_wzy_F | GTTTGGTTGGGATAGGTGC | *wzy* PCR |
| Psgc 56_wzy_R | AAATAACCGACATCCGTCTC | *wzy* PCR |
| PSgc 4_wzy_F | GGGTTGAAGTTGGTTGGGGAT | *wzy* PCR |
| PSgc 4_wzy_R | CCCAAAAGACTAACACTTTCAGC | *wzy* PCR |
| Psgc 13_wzy_F | TTCTTTAGGGGTGTTTGGTG | *wzy* PCR |
| Psgc 13_wzy_R | GTAACACCTCCCCCATCATC | *wzy* PCR |
| PSgc 14_wzy_F | AGCCCATAAAGTCACCTTC | *wzy* PCR |
| PSgc 14_wzy_R | ACACCCTTTAACCAAGCCC | *wzy* PCR |
| PSgc 15_wzy_F | ACGCTAGCCAAGCTGATAG | *wzy* PCR |
| PSgc 15_wzy_R | CCACCCCAAACCATAACCC | *wzy* PCR |
| PSgc 17_wzy_F | AACATAGGAGTCGAACCTG | *wzy* PCR |
| PSgc 17_wzy_R | TGTCTAAGAGACAAGGCTG | *wzy* PCR |
| PSgc 24_wzy_F | CCACCAGCATTATCTGGCG | *wzy* PCR |
| PSgc 24_wzy_R | CAAAGCAACCGTACGCATC | *wzy* PCR |
| PSgc 25_wzy_F | GGCAATCTGTAGCAATCGC | *wzy* PCR |
| PSgc 25_wzy_R | GGCAACCATGCATGAGACC | *wzy* PCR |
| PSgc 44_wzy_F | GATTTAATGTGGTGGCAATATC | *wzy* PCR |
| PSgc 44_wzy_R | CATTCTGATGTATAAACTGGTGC | *wzy* PCR |
| Psgc 52_wzy_F | TGCAGGCCGGTAATCTTTG | *wzy* PCR |
| Psgc 52_wzy_R | AAGCGCCGATTGAACATGC | *wzy* PCR |
| KL24_wzy_F | TAGTACAAATGCGCCTCCC | *wzy* PCR |
| KL24_wzy_R | AGCCCCTTTTCTTAACCGC | *wzy* PCR |
| KL36_wzy_F | TCTACATCTAATGTTGAGCC | *wzy* PCR |
| KL36_wzy_R | AGCCTTACAGCAAAGAAGG | *wzy* PCR |
| KL37_wzy_F | AGAACTGGGGTTGAAGTTGG | *wzy* PCR |
| KL37_wzy_R | AATACATTAGACAAGCGGCC | *wzy* PCR |
| KL44_wzy_F | TCCAACTAAGTTATGTCGC | *wzy* PCR |
| KL44_wzy_R | GCGCAGCATTATTTTGTGC | *wzy* PCR |
| KL47_wzy_F | TGGCTATCTGGCTTTGCAC | *wzy* PCR |
| KL47_wzy_R | GTGAAACGGCACCCAAAAG | *wzy* PCR |
| KL48_wzy_F | TCATTTCCACATTACCTTG | *wzy* PCR |
| KL48_wzy_R | TTAGGCAAGCTATAGCAACC | *wzy* PCR |
| KL49_wzy_F | TGTGGTTTGGTTTGGCACC | *wzy* PCR |
| KL49_wzy_R | AAAAGATTGTGGGCAAGCC | *wzy* PCR |
| KL105_wzy_F | AGCCAAGTCAAGCAGAGTG | *wzy* PCR |
| KL105_wzy_R | GGATCGTTTGTGACTCAGAG | *wzy* PCR |
| KL106_wzy_F | GGCAAGGTATTGCAATTGG | *wzy* PCR |
| KL106_wzy_R | GGAATCAGAAAAACTGGACC | *wzy* PCR |
| cgmA-F | ATGAAGGCAACTCCTTGGC | *cgmA* PCR |
| cgmA-R | TCAAACTCGACATTCGCCG | *cgmA* PCR |

Supplementary Table 5. *wzc* typing of 82 clinical strains

| Strain (no.) | *wzc* allele | K-types of *wzc* allele | No. of strains | K type^a^ | DNA identity to the allele | DNA identity to the corresponding K-type |
| --- | --- | --- | --- | --- | --- | --- |
| CRAB (39) | 8 | KL2, KL42, KL58, KL81, KL93 | 12 | KL2^c^ | 97-100% | 99-100% |
|  | 6 | KL3, KL22 | 9 | KL22^d^ | 99-100% | 99-100% |
|  | 3 | KL10_G2, KL14, KL43, KL52, KL37, KL48, KL116 | 7 | KL52 | 97-100% | 99-100% |
|  | 15 | KL10_G1, KL80 | 7 | KL10 | 97-100% | 99-100% |
|  | 9 | KL1, KL107 | 2 | KL1/KL107^e^ | 100% | 100% |
|  | 13 | KL45, KL47, KL88, KL102 | 1 | KL47/KL102^e^ | 98-100% | 99-100% |
|  | 33 | KL49 | 1 | KL49 | 100% | 100% |
| non-CRAB(43) | 3 | KL10_G2, KL14, KL43, KL52, KL37, KL48, KL116 | 9 | KL14 | 94-97% | 94-95% |
|  |  |  | 2 | KL116/KL37^e^ | 93-96% | 93-96% |
|  |  |  | 1 | KL52 | 99% | 99% |
|  |  |  | 1 | KL48 | 97-99% | 98% |
|  |  |  | 5 | unknown | 93-100% | - |
|  | 35 | KL106 | 5 | KL106 | 99% | 99% |
|  | 8 | KL2, KL42, KL58, KL81, KL93 | 4 | KL2 | 97-100% | 99-100% |
|  | 11 | KL11, KL29 | 1 | KL11/ KL29^e^ | 100% | 100% |
|  | 23 | KL12, KL13, KL73 | 1 | KL13/KL73^e^ | 99% | 99% |
|  | 30 | KL24 | 1 | KL24 | 100% | 100% |
|  | 13 | KL45, KL47, KL88, KL102 | 1 | KL47/KL102^e^ | 98-100% | 99-100% |
|  | 5 | KL26, KL83, KL105 | 1 | KL105/KL83^e^ | 99% | 99% |
|  | 16 | KL63 | 1 | KL63 | 100% | 100% |
|  |  |  | 1 | unknown | 99% | - |
|  | 17 | KL30^b^, KL89 | 2 | unknown | 91-100% | - |
|  | 32 | KL44 | 2 | unknown | 96% | - |
|  | 9 | KL1, KL107 | 1 | unknown | 99% | - |
|  | 10 | KL85 | 1 | unknown | 96% | - |
|  | 24 | KL5^b^ | 1 | unknown | 87% | - |
|  | 26 | KL36 | 1 | unknown | 95% | - |
|  | 25 | KL8 | 1 | unknown | 89% | - |

CRAB, carbapenem-resistant *A. baumannii*

a, *wzy*-PCR genotyping; b, *wzy* gene was not found in *cps* locus, -, not available

c, KL2 and KL81 share a *wzy* gene with three nucleotide difference and thus were further distinguished by *cgmA* PCR (*cgmA* was present in KL81 but not in KL2). d, KL3 and KL22 share a *wzy* with only one nucleotide difference, and thus were further distinguished by *cgmA* PCR (*cgmA* was present in KL22 but not in KL3). e, KL1/KL107, KL13/KL73, KL47/KL102, KL11/ KL29, KL116/KL37, KL105/KL83 were undistinguished by *wzy* PCR

*Wzc* sequences included in *wzc* panel

>7804 [accession no. CP022283.1]

ttttcactgattgctcagtggaagcttattgctctatgcgttattttaagcgttgtgtgtgctctactctatttacgtgtaacgccagatacctattcggtagatgctttggttcaggttgaagacagcaagggtgcttctgctgcgcttttaggtgatttatcacaaatgatcgagcaaaaatcaccagctcaggcagaaatagaaattttaaaatctcgtttggttttaggttctgtgattaaagatttacatcttaatatacaggtctctagcacagagaatactttcactcatcgcttactaagtaatccagaatatcaaactgaatataattcgaaatcagtcatttttaaggatggtttaaaaagttttgatatccggcagtttgagattcctacttattacctagataagaacttacttcttgattttgataaacagtctttacgtttaaccgatcctgcaactgaagaagttatcttaactgttccactaaaccaagttaaccaagttacaggtcctcatggtgtatggaaagttgctatctttacaaaagatcaatttgacgcgacctataatattaaaagtttatctttacctattgctgtaaatgcgattagtgcaaattatgctgtggccgaacgtggtaaacttactggggttttaggcttaacttatcaaggacaagataaagagcatattaccaaagtcttaaatgctattttagcgacttatagtgcgcaaaatattga

>TCDC_AB0715[accession no. CP002522]

ttttcactgattgctcagtggaagcttattgctctatgcgttattttaagcgttgtgtgtgctctactctatttacgtgtaacgccagatacctattcggtagatgctttggttcaggttgaagacagcaagggtgcttctgctgcgcttttaggtgatttatcacaaatgatcgagcaaaaatcaccagctcaggcagaaatagaaattttaaaatctcgtttggttttaggttctgtgattaaagatttacatcttaatatacaggtctctagcacagagaatactttcactcatcgcttactaagtaatccagaatatcaaactgaatataattcgaaatcagtcatttttaaggatggtttaaaaagttttgatatccggcagtttgagattcctacttattacctagataagaacttacttcttgattttgataaacagtctttacgtttaaccgatcctgcaactgaagaagttatcttaactgttccactaaaccaagttaaccaagttacaggtcctcatggtgtatggaaagttgctatctttacaaaagatcaatttgacgcgacctataatattaaaagtttatctttacctattgctgtaaatgcgattagtgcaaattatgctgtggccgaacgtggtaaacttactggggttttaggcttaacttatcaaggacaagataaagagcatattaccaaagtcttaaatgctattttagcgacttatagtgcgcaaaatattga

>LUH5534 [accession no. KC526908]

ttttcactgattgctcagtggaaactcattgctctatgcgttattttaagcgttgtatgtgccctactctatttacgtgtaacgccagatacctattcggtagatgctttagttcaggttgaggacagtaaaggggcttctgctgcacttttaggtgatctctcacaaatgattgagcaaaaatcaccagctcaagcagaaatagaaattttaaaatctcgtttggttttaggcacggtcattaaggacttacatctaaatatacaggtatctagtacagaaaatacgcttacccatcgcttattaagtgatactgactataaaactgaatacactaaaaaatcggttttatttaaagatgacttaaaaagttttgaagtacgtgaatttgaagttccagcttactatcttgacagaaacttacttcttaattttgacaaacaatctttacgtctaaccgatcctgcaactgaagaagtcatcttaactgttccattaaaccaagctaatcacgttgctggtcctcatggtgtatggaaagttgctatctttacgaaagatcaatttgacgcgacctataatattaaaagtcaatcaataccagctgctgtaaatgcacttagcacaaattattcagtagcagaacgcggcaaacttacaggagtgttaggtcttaactatcaaggtcaagacaaagagcatattaccaaagttctcaatgcgattttggcgacctatagcgcacaaaatattga

>LUH5536_ [accession no. KC526912]

ttttcattgattgcacagtggaaaatcatagcatgctgcgtggtcttaagtttaatttgtgccttgctatacttacggataacgccagatacttattctgtagatgcaatggtacaagttgaggacagtaaaggcgctgcctctgctgctttgcttggtgacctttctaaagtcagtggaggtctatctcaaaaatcaccagctgatccagaaatagagattcttcgttctagaatggttttaggacaagtcatccagaaccttaatcttgatattaacgtcaaagataaccaatcaggattgatcgacaagttaatttcacaagataagagtagacttgagtatcgtcatgaatctgtactgtacagtaatcaaaataataatttgattatacgagaacttaaagtacctgaatattatttagataaacccttaaagttagagttcaaaggtgcaaatcaattcactttaacatacaaagatcaagttgtatttaatggtcaattaaacaaaaaaaatatactaaatacaaatagaggtctttggcaggtacaacttaatgctcaaggtaacctgaaagaacaaagctatactttaactaaactcgctctaccaacagccgttaaaaattttaatgatatctatagtgttgctgaaaaaggtaaagcgactggtgttattggtttaaattatctaggtcaagatcctgagcacattacacaagtactaaataatgttctaactgtttatcaccaacaaaacattga

>LUH3484_ [accession no. KC526895]

ttttcactgattgctcagtggaaactcattgctctatgcgttattttaagcgttgtatgtgccctactctatttacgtgtaacgccagatacctattcggtagatgctttagttcaggttgaggacagtaaaggggcttctgctgcacttttaggtgatctatcacaaatgattgagcaaaaatcaccagctcaagcagaaatagaaattttaaaatctcgtttggttttaggcacggtcattaaggacttacatctaaatatacaggtctctagcactgaaaatacacttacccatcgcttattaagtgataccgactataaaactgaatacactaaaaaatcggttttatttaaagatgacttaaaaagttttgcagtacgtgaatttgaagttccagcttactatcttgacagaaacttacttcttaattttgataaacagtctttacgtttaaccgatcctgcaactgaagaagtcatcttaactgttccattaaaccaagctaatcacgttgctggtcctcatggtttatggaaagttgctatctttacgaaagatcaatttgacgcgacctataatattaaaagtcaatcaataccagctgctgtaaatgcacttagcgcaaattattcagtagcagaacgcggcaaacttacaggagtgttaggtcttaactatcaaggtcaagataaagaacaaatcactaaagttttaaatgccattttagctacctatagtgcccaaaatattga

>RUH134_KL9 [accession no. CP032055.1]

ttttcactgattgctcagtggaaactcattgctctatgcgttattttaagcgttgtatgtgccctactctatttacgtgtaacgccagatacctattcggtagatgctttagttcaggttgaggacagtaaaggggcttctgctgcacttttaggtgatctatcacaaatgattgagcaaaaatcaccagctcaagcagaaatagaaattttaaaatctcgtttggttttaggcacggtcattaaggacttacatctaaatatacaggtctctagcactgaaaatacacttacccatcgcttattaagtgataccgactataaaactgaatacactaaaaaatcggttttatttaaagatgacttaaaaagttttgcagtacgtgaatttgaagttccagcttactatcttgacagaaacttacttcttaattttgataaacagtctttacgtttaaccgatcctgcaactgaagaagtcatcttaactgttccattaaaccaagctaatcacgttgctggtcctcatggtttatggaaagttgctatctttacgaaagatcaatttgacgcgacctataatattaaaagtcaatcaataccagctgctgtaaatgcacttagcgcaaattattcagtagcagaacgcggcaaacttacaggagtgttaggtcttaactatcaaggtcaagataaagaacaaatcactaaagttttaaatgccattttagctacctatagtgcccaaaatattga

>KL9-MDR-TJ [CP003500]

ttttcactgattgctcagtggaaactcattgctctatgcgttattttaagcgttgtatgtgccctactctatttacgtgtaacgccagatacctattcggtagatgctttagttcaggttgaggacagtaaaggggcttctgctgcacttttaggtgatctatcacaaatgattgagcaaaaatcaccagctcaagcagaaatagaaattttaaaatctcgtttggttttaggcacggtcattaaggacttacatctaaatatacaggtctctagcactgaaaatacacttacccatcgcttattaagtgataccgactataaaactgaatacactaaaaaatcggttttatttaaagatgacttaaaaagttttgcagtacgtgaatttgaagttccagcttactatgttgacagaaacttacttcttaattttgataaacagtctttacgtttaaccgatcctgcaactgaagaagtcatcttaactgttccattaaaccaagctaatcacgttgctggtcctcatggtttatggaaagttgctatctttacgaaagatcaatttgatgcgacctataatattaaaagtcaatcaataccagctgctgtaaatgcacttagcgcaaattattcagtagcagaacgcggcaaacttacaggagtgttaggtcttaactatcaaggtcaagataaagaacaaatcactaaagttttaaatgccattttagctacctatagtgcccaaaatattga

>KL35-LUH5535 [KC526896]

ttttcactcattgcacagtggaagcttatcgcactctgcgttattttaagtcttgtatgtgctttaatctacttacgtgtaactccaaacacctattcagttgatgctcttgttcaggtagaagatagtaaaggagcttctgcagctttacttggtgatttatcaaatatgatagagcaaaaatcacctgctcaagctgaaattgaaattttaaaatctcggttggtactgggttctgtcattaaagaattacatctgaatatacaagtttccagtacagaaaatacttttagccatcgcttattaagcgatactgactataaaactgaatactctaaaaaatcagttatctttaaggacggtctcaaaagttttgagattcgtacattagaagttccagcctattatttggataagaccttacatcttaactttgataaacaatctgtacgtttaactgacccagcaacggaacaaactcttttaacgataccacttaatcagctaaaccaagtcactggtccatatggcacttggaaagtcggtatttttactaaagatcagtttaataccacttataatattaaaaacctatctgttcctgctgctgtaaatgcgattagttcaaaatactctgtagctgaacgcggcaaacttactggagttttgggcttaaattatcaaggtcaagacaaagagcacattactaaagttttaaatgcaattttagcaacttatagcgcgcaaaatattga

>KL83-LUH5538 [KC526898]

ttttcattgattgcacagtggaaaatcatagcatgctgcgtgatcttaagtttaatttgtgccttgttatatttacgcataacaacagatacttattctgtagatgctatggtacaagttgaagacagtaaaggcgctgcctctgctgctttattgggagacctttctaaagttactggaggtatatctcaaaaatcaccagctgatccagaaatagagattttaaaatctcgtatggttttagggcaagtgattcataaccttaatcttgatattaaaattaaagataatcaatcaggattgattgataagttaatttcacaagataagagtaaaattgaatatcgtcatgaatcggtagtttacaataacttaaacgctagtttgattattcaagaatttaaggtgcctgaatattattttgataaacctttgaaattagagtttaaagatacaaatcaatttactctaatgtataaagatcaagttgtatttaatggtcaattaaacaagaaaaatatattaaattcaaataaaggtttatggcaagttcaaatcaatgcacaaggcaatttaaaagatcaaagttatactttaactaaattagctcttttaacagctgtaaatcaatttaattcaatatattcagttaatgaaaaaggtaaaatgaccggagttatcggtttaagctatttaggccaagatcctgaacatattactcaagtgctaaataatgttctaaatgtttatcatgaacaaaatattga-

>KL83-ABOB15 [LLJE01000049]

ttttcattgattgcacagtggaaaatcatagcatgctgcgtgatcttaagtttaatttgtgccttgttatatttacgcataacaacagatacttattctgtagatgctatggtacaagttgaagacagtaaaggcgctgcctctgctgctttattgggagacctttctaaagttactggaggtatatctcaaaaatcaccagctgatccagaaatagagattttaaaatctcgtatggttttagggcaagtgattcataaccttaatcttgatattaaaattaaagataatcaatcaggattgattgataagttaatttcacaagataagagtaaaattgaatatcgtcatgaatcggtagtttacaataacttaaacgctagtttgattattcaagaatttaaggtgcctgaatattattttgataaacctttgaaattagagtttaaagatacaaatcaatttactctaatgtataaagatcaagttgtatttaatggtcaattaaacaagaaaaatatattaaattcaaataaaggtttatggcaagttcaaatcaatgcacaaggcaatttaaaagatcaaagttatactttaactaaattagctcttttaacagctgtaaatcaatttaattcaatatattcagttaatgaaaaaggtaaaatgaccggagttatcggtttaagctatttaggccaagatcctgaacatattactcaagtgctcaataatgttctaaatgtttatcatgaacaaaatattga-

>LUH5539_[KC526915]

ttttcactgattgctcagtggaaactcattgctctatgcgttattttaagcgttgtatgtgccctactatatttacgtgtaacgccagatacctattcggtagatgcgttggttcaggttgaggacagtaaaggggcttctgctgcacttttaggtgatctctcacaaatgattgagcaaaaatcaccggctcaagcagaaatagaaattttaaaatctcgtttggttttaggcacggtcattaaggacttacatctaaatatacaggtctctagcactgaaaatacgcttacccatcgcttattgagtgatgctgactataaaactgaatatactacgaaatcagtcatttttaaggatggcttaaaaagttttgatatccgtcagtttgagattcctgcttattacttagataagaacttacttcttgattttgataaacaatctttacgtttaaccgatcctgtaactgaagaagtcatcttaactgttccattaaaccaagttaaccatgttgctggtcctcatggtttatggaaagttgctatctttacaaaagaccaatttgacgcaacctataatattaaaagtctatctttacctattgctgtaaatgctattagtgcgaattatgctgtggccgaacgcggcaaacttacaggagtgttaggtcttaactatcaaggtcaagacaaagagcatattaccaaagttctcaatgcgatcttagcgacctatagtgcacaaaatattga

>LUH5537 [KC526920]

ttttcactgattgctcagtggaaactcattgctctatgcgttattttaagcgttgtatgtgccctactatatttacgtgtaacgccagatacctattcggtagatgcgttggttcaggttgaggacagtaaaggggcttctgctgcacttttaggtgatctctcacaaatgattgagcaaaaatcaccggctcaagcagaaatagaaattttaaaatctcgtttggttttaggcacggtcattaaggacttacatctaaatatacaggtctctagcactgaaaatacgcttacccatcgcttattgagtgatgctgactataaaactgaatatactacgaaatcagtcatttttaaggatggcttaaaaagttttgatatccgtcagtttgagattcctgcttattacttagataagaacttacttcttgattttgataaacaatctttacgtttaaccgatcctgtaactgaagaagtcatcttaactgttccattaaaccaagttaaccatgttgctggtcctcatggtttatggaaagttgctatctttacaaaagaccaatttgacgcaacctataatattaaaagtctatctttacctattgctgtaaatgctattagtgcgaattatgctgtggccgaacgcggcaaacttacaggagtgttaggtcttaactatcaaggtcaagacaaagagcatattaccaaagttctcaatgcgatcttagcgacctatagtgcacaaaatattga

>ATCC_17978_KL3 [CP000521]

ttttcactgattgctcagtggaaactcattgctctatgcgttattttaagcgttgtatgtgccctactatatttacgtgtaacgccagatacctattcggtagatgctttagttcaggttgaggacagtaaaggggcttctgctgcacttttaggtgatctctcacaaatgattgagcaaaaatcaccggctcaagcagaaatagaaattttaaaatctcgtttggttttaggcacggtcattaaggacttacatctaaatatacaggtctctagcactgaaaatacgcttacccatcgcttattgagtgatgctgactataaaactgaatatactacgaaatcagtcatttttaaggatggcttaaaaagttttgatatccgtcagtttgagattcctgcttattacttagataagaacttacttcttgattttgataaacaatctttacgtttaaccgatcctgtaactgaagaagtcatcttaactgttccattaaaccaagttaaccatgttgctggtcctcatggtttatggaaagttgctatctttacaaaagaccaatttgacgcaacctataatattaaaagtctatctttacctattgctgtaaatgctattagtgcgaattatgctgtggccgaacgcggcaaacttacaggagtgttaggtcttaactatcaaggtcaagacaaagagcatattaccaaagttctcaatgcgatcttagcgacctatagtgcacaaaatattga

>LUH5540 [KC526902]

ttttcattgattgcacagtggaaaatcatagcatgctgcgtggtcttaagtttaatttgtgccttgctatatttacgtataacgccagacacctacgctgtagatgcaatggtacaagttgaagacagtaaaggcgctgcctctgctgctctacttggggatctttccaaggttagcggaggtctatctcaaaaatctccagctgatccagaaatagagattcttcgttctagaatggttttaggacaagtgattcataaccttaatcttgatattaaaattaaagataatcaattaggtttgattggtaaacttgtttcacaagataaaagtaaactcgaatattatcatgatgctgtaacttatactaatcaaaacaatagattaatcgttaaacaacttagtgtacctgaatactatttagataagcccctaaaattagagtttaaagatcttaatcaatttactctgacctataaagatcaagttgtttttaatggatctctaaacaaaaaaaatgtattcaatactgaaaaagggttatggcaagtacaacttaatgcccaaggtgacttgaaaaaacagagcttcactttaactaaactcgctcttccaacagctgttaaaaattttaataatatttatggtgttgcagaaaaaggaaagatgactggtgttattggtttaagctacttaggtcaagatccagaacatattacccaagtactcaataacgttctaaatatttatcatgtacaaaacattga-

>KL14-LUH5541_[KC526906]

ttttcattgattgcacagtggaaaatcatagcatgctgcgtggtcttaagtttaatttgtgccttgctatacttacggataacgccagatacttattctgtagatgcaatggtacaagttgaggacagtaaaggcgctgcctctgctgctttgcttggtgacctttctaaagtcagtggaggtctatctcaaaaatcaccagctgatccagaaatagagattcttcgttctagaatggttttaggacaagtcatccagaaccttaatcttgatattaacgtcaaagataaccaatcaggattgatcgacaagttaatttcacaagataagagtagacttgagtatcgtcatgaatctgtactgtacagtaatcaaaataataatttgattatacgagaacttaaagtacctgaatattatttagataaacccttaaagttagagttcaaaggtgcaaatcaattcactttaacatacaaagatcaagttgtatttaatggtcaattaaacaaaaaaaatatactaaatacaaatagaggtctttggcaggtacaacttaatgctcaaggtaacctgaaagaacaaagctatactttaactaaactcgctctaccaacagccgttaaaaattttaatgatatctatagtgttgctgaaaaaggtaaagtgactggtgttattggtttaaattatctaggtcaagatcctgagcacattacacaagtactaaataatgttctaactgtttatcaccaacaaaacattga-

>KL14-D46 [KF030679.2]

ttttcattgattgcacagtggaaaatcatagcatgctgcgtggtcttaagtttaatttgtgccttgctatacttacggataacgccagatacttattctgtagatgcaatggtacaagttgaggacagtaaaggcgctgcctctgctgctttgcttggtgacctttctaaagtcagtggaggtctatctcaaaaatcaccagctgatccagaaatagagattcttcgttctagaatggttttaggacaagtcatccagaaccttaatcttgatattaacgtcaaagataaccaatcaggattgatcgacaagttaatttcacaagataagagtagacttgagtatcgtcatgaatctgtactgtacagtaatcaaaataataatttgattatacgagaacttaaagtacctgaatattatttagataaacccttaaagttagagttcaaaggtgcaaatcaattcactttaacatacaaagatcaagttgtatttaatggtcaattaaacaaaaaaaatatactaaatacaaatagaggtctttggcaggtacaacttaatgctcaaggtaacctgaaagaacaaagctatactttaactaaactcgctctaccaacagccgttaaaaattttaatgatatctatagtgttgctgaaaaaggtaaagtgactggtgttattggtttaaattatctaggtcaagatcctgagcacattacacaagtactaaataatgttctaactgtttatcaccaacaaaacattga-

>LUH3713_[KC526916]

ttttcactgattgcacagtggaaactcattgctctatgcgttattttaagcgttgtatgtgcactactctatttacgtgtaacaccagatacctattcggtagatgctttagttcaggttgaggacagcaagggagcttctgctgcacttttaggcgatctatcacaaatgatcgagcaaaaatcaccagctcaagcagaaatagaaattttaaaatctcgtttggttttaggttctgttattaaagatttacatctgaatatacaggtctctagcactgaaaatacatttacacatcgcttattgagtgatactgattacaaaactgaatatgctaaaaaatcggttttatttaaagatggtttaaaaagttttgatatacgtcagtttgagattccagcttattacttagataagaacttacttcttgattttgataaacagtctttacgtttaacagatccagatactgaagaagtcatcttaactgttccattaaaccaagctaacagcgttacaggaccgtatggtgtatggaaagttgctatatttacaaacgaccaatttgaatcaacttataatattaagaaattatcacttcctttagctataaaatctatcagttccgactattctgtagaagaaaaaggtaaactcacaggtatattaggacttagctatcaagggcaagatcaagaacatattactaaagtcttaaatgctattttagcgacctatagtgcacaaaatattga

>KL2-AB_1582-8 [AMHB00000000]

ttttcattgattgctcagtggaaactcattgctctatgcgttattttaagcgttgtatgtgccctactctatttacgtgtaacgccagatacctattcggtagatgctttggttcaggttgaagacagtaaaggggcttctgctgcacttttaggtgatctctcacaaatgattgagcaaaaatcaccagctcaagcagaaatagaaattttaaaatctcgtttggttttaggttctgtgattaaagatttacatctgaatatacaggtctctagcactgagaatacatttacacatcgcttattgagtgatactgattataaaactgaatatgctaaaaaatcggttttatttaaagatggtttaaaaagttttgatatacgtcagtttgagattccagcttattatctagataagaacttacttcttgactttgataaacagtctttacgtttaacagacccagatactgaagaagtcatcttaactgttccattaaaccaagctaacagcgttacaggaccgtatggtttatggaaagttgctatatttacaaaagaccaatttgactcaacttataatattaagaaattatcacttcctttagctataaaatctatcagttccgactattctgtagaagaaaaaggtaaactcacaggtatattaggacttagctatcaaggccaagatcaagaacatattactaaagtcttaaatgctattttagcgacttatagtgcacaaaatattga

>KL2-3990 [AEOY00000000]

ttttcattgattgctcagtggaaactcattgctctatgcgttattttaagcgttgtatgtgccctactctatttacgtgtaacgccagatacctattcggtagatgctttggttcaggttgaagacagtaaaggggcttctgctgcacttttaggtgatctctcacaaatgattgagcaaaaatcaccagctcaagcagaaatagaaattttaaaatctcgtttggttttaggttctgtgattaaagatttacatctgaatatacaggtctctagcactgagaatacatttacacatcgcttattgagtgatactgattataaaactgaatatgctaaaaaatcggttttatttaaagatggtttaaaaagttttgatatacgtcagtttgagattccagcttattatctagataagaacttacttcttgactttgataaacagtctttacgtttaacagacccagatactgaagaagtcatcttaactgttccattaaaccaagctaacagcgttacaggaccgtatggtttatggaaagttgctatatttacaaaagaccaatttgactcaacttataatattaagaaattatcacttcctttagctataaaatctatcagttccgactattctgtagaagaaaaaggtaaactcacaggtatattaggacttagctatcaaggccaagatcaagaacatattactaaagtcttaaatgctattttagcgacttatagtgcacaaaatattga

>KL2-Ab11111 [AKAQ00000000]

ttttcattgattgctcagtggaaactcattgctctatgcgttattttaagcgttgtatgtgccctactctatttacgtgtaacgccagatacctattcggtagatgctttggttcaggttgaagacagtaaaggggcttctgctgcacttttaggtgatctctcacaaatgattgagcaaaaatcaccagctcaagcagaaatagaaattttaaaatctcgtttggttttaggttctgtgattaaagatttacatctgaatatacaggtctctagcactgagaatacatttacacatcgcttattgagtgatactgattataaaactgaatatgctaaaaaatcggttttatttaaagatggtttaaaaagttttgatatacgtcagtttgagattccagcttattatctagataagaacttacttcttgactttgataaacagtctttacgtttaacagacccagatactgaagaagtcatcttaactgttccattaaaccaagctaacagcgttacaggaccgtatggtttatggaaagttgctatatttacaaaagaccaatttgactcaacttataatattaagaaattatcacttcctttagctataaaatctatcagttccgactattctgtagaagaaaaaggtaaactcacaggtatattaggacttagctatcaaggccaagatcaagaacatattactaaagtcttaaatgctattttagcgacttatagtgcacaaaatattga

>KL2-OIFC087[AMFS00000000]

ttttcattgattgctcagtggaaactcattgctctatgcgttattttaagcgttgtatgtgccctactctatttacgtgtaacgccagatacctattcggtagatgctttggttcaggttgaagacagtaaaggggcttctgctgcacttttaggtgatctctcacaaatgattgagcaaaaatcaccagctcaagcagaaatagaaattttaaaatctcgtttggttttaggttctgtgattaaagatttacatctgaatatacaggtctctagcactgagaatacatttacacatcgcttattgagtgatactgattataaaactgaatatgctaaaaaatcggttttatttaaagatggtttaaaaagttttgatatacgtcagtttgagattccagcttattatctagataagaacttacttcttgactttgataaacagtctttacgtttaacagacccagatactgaagaagtcatcttaactgttccattaaaccaagctaacagcgttacaggaccgtatggtttatggaaagttgctatatttacaaaagaccaatttgactcaacttataatattaagaaattatcacttcctttagctataaaatctatcagttccgactattctgtagaagaaaaaggtaaactcacaggtatattaggacttagctatcaaggccaagatcaagaacatattactaaagtcttaaatgctattttagcgacttatagtgcacaaaatattga

>KL2_A74[KJ459911.1]

ttttcattgattgctcagtggaaactcattgctctatgcgttattttaagcgttgtatgtgccctactctatttacgtgtaacgccagatacctattcggtagatgctttggttcaggttgaagacagtaaaggggcttctgctgcacttttaggtgatctctcacaaatgattgagcaaaaatcaccagctcaagcagaaatagaaattttaaaatctcgtttggttttaggttctgtgattaaagatttacatctgaatatacaggtctctagcactgagaatacatttacacatcgcttattgagtgatactgattataaaactgaatatgctaaaaaatcggttttatttaaagatggtttaaaaagttttgatatacgtcagtttgagattccagcttattatctagataagaacttacttcttgactttgataaacagtctttacgtttaacagacccagatactgaagaagtcatcttaactgttccattaaaccaagctaacagcgttacaggaccgtatggtttatggaaagttgctatatttacaaaagaccaatttgactcaacttataatattaagaaattatcacttcctttagctataaaatctatcagttccgactattctgtagaagaaaaaggtaaactcacaggtatattaggacttagctatcaaggccaagatcaagaacatattactaaagtcttaaatgctattttagcgacttatagtgcacaaaatattga

>KL2-AB_515_8 [AMHU00000000]

ttttcattgattgctcagtggaaactcattgctctatgcgttattttaagcgttgtatgtgccctactctatttacgtgtaacgccagatacctattcggtagatgctttggttcaggttgaagacagtaaaggggcttctgctgcacttttaggtgatctctcacaaatgattgagcaaaaatcaccagctcaagcagaaatagaaattttaaaatctcgtttggttttaggttctgtgattaaagatttacatctgaatatacaggtctctagcactgagaatacatttacacatcgcttattgagtgatactgattataaaactgaatatgctaaaaaatcggttttatttaaagatggtttaaaaagttttgatatacgtcagtttgagattccagcttattatctagataagaacttacttcttgactttgataaacagtctttacgtttaacagacccagatactgaagaagtcatcttaactgttccattaaaccaagctaacagcgttacaggaccgtatggtttatggaaagttgctatatttacaaaagaccaatttgactcaacttataatattaagaaattatcacttcctttagctataaaatctatcagttccgactattctgtagaagaaaaaggtaaactcacaggtatattaggacttagctatcaaggccaagatcaagaacatattactaaagtcttaaatgctattttagcgacttatagtgcacaaaatattga

>KL2-AB_1583_8 [AMHC00000000]

ttttcactgattgcacagtggaaactcattgctctatgcgttattttaagcgttgtatgtgcactactctatttacgtgtaacaccagatacctattcggtagatgctttagttcaggttgaggacagcaagggagcttctgctgcacttttaggcgatctatcacaaatgatcgagcaaaaatcaccagctcaagcagaaatagaaattttaaaatctcgtttggttttaggttctgttattaaagatttacatctgaatatacaggtctctagcactgaaaatacatttacacatcgcttattgagtgatactgattacaaaactgaatatgctaaaaaatcggttttatttaaagatggtttaaaaagttttgatatacgtcagtttgagattccagcttattacttagataagaacttacttcttgattttgataaacagtctttacgtttaacagatccagatactgaagaagtcatcttaactgttccattaaaccaagctaacagcgttacaggaccgtatggtgtatggaaagttgctatatttacaaacgaccaatttgaatcaacttataatattaagaaattatcacttcctttagctataaaatctatcagttccgactattctgtagaagaaaaaggtaaactcacaggtatattaggacttagctatcaagggcaagatcaagaacatattactaaagtcttaaatgctattttagcgacctatagtgcacaaaatattga

>KL2-AB_1595_8 [AMHE00000000]

ttttcattgattgctcagtggaaactcattgctctatgcgttattttaagcgttgtatgtgccctactctatttacgtgtaacgccagatacctattcggtagatgctttggttcaggttgaagacagtaaaggggcttctgctgcacttttaggtgatctctcacaaatgattgagcaaaaatcaccagctcaagcagaaatagaaattttaaaatctcgtttggttttaggttctgtgattaaagatttacatctgaatatacaggtctctagcactgagaatacatttacacatcgcttattgagtgatactgattataaaactgaatatgctaaaaaatcggttttatttaaagatggtttaaaaagttttgatatacgtcagtttgagattccagcttattatctagataagaacttacttcttgactttgataaacagtctttacgtttaacagacccagatactgaagaagtcatcttaactgttccattaaaccaagctaacagcgttacaggaccgtatggtttatggaaagttgctatatttacaaaagaccaatttgactcaacttataatattaagaaattatcacttcctttagctataaaatctatcagttccgactattctgtagaagaaaaaggtaaactcacaggtatattaggacttagctatcaaggccaagatcaagaacatattactaaagtcttaaatgctattttagcgacttatagtgcacaaaatattga

>KL2-AB_1766_8 [AMJO00000000]

ttttcattgattgctcagtggaaactcattgctctatgcgttattttaagcgttgtatgtgccctactctatttacgtgtaacgccagatacctattcggtagatgctttggttcaggttgaagacagtaaaggggcttctgctgcacttttaggtgatctctcacaaatgattgagcaaaaatcaccagctcaagcagaaatagaaattttaaaatctcgtttggttttaggttctgtgattaaagatttacatctgaatatacaggtctctagcactgagaatacatttacacatcgcttattgagtgatactgattataaaactgaatatgctaaaaaatcggttttatttaaagatggtttaaaaagttttgatatacgtcagtttgagattccagcttattatctagataagaacttacttcttgactttgataaacagtctttacgtttaacagacccagatactgaagaagtcatcttaactgttccattaaaccaagctaacagcgttacaggaccgtatggtttatggaaagttgctatatttacaaaagaccaatttgactcaacttataatattaagaaattatcacttcctttagctataaaatctatcagttccgactattctgtagaagaaaaaggtaaactcacaggtatattaggacttagctatcaaggccaagatcaagaacatattactaaagtcttaaatgctattttagcgacttatagtgcacaaaatattga

>KL2-AB_2008_23_07_01_7 [AMHR00000000]

ttttcattgattgctcagtggaaactcattgctctatgcgttattttaagcgttgtatgtgccctactctatttacgtgtaacgccagatacctattcggtagatgctttggttcaggttgaagacagtaaaggggcttctgctgcacttttaggtgatctctcacaaatgattgagcaaaaatcaccagctcaagcagaaatagaaattttaaaatctcgtttggttttaggttctgtgattaaagatttacatctgaatatacaggtctctagcactgagaatacatttacacatcgcttattgagtgatactgattataaaactgaatatgctaaaaaatcggttttatttaaagatggtttaaaaagttttgatatacgtcagtttgagattccagcttattatctagataagaacttacttcttgactttgataaacagtctttacgtttaacagacccagatactgaagaagtcatcttaactgttccattaaaccaagctaacagcgttacaggaccgtatggtttatggaaagttgctatatttacaaaagaccaatttgactcaacttataatattaagaaattatcacttcctttagctataaaatctatcagttccgactattctgtagaagaaaaaggtaaactcacaggtatattaggacttagctatcaaggccaagatcaagaacatattactaaagtcttaaatgctattttagcgacttatagtgcacaaaatattga

>KL2-AB_2009_04_02_7[AMHT00000000]

ttttcattgattgctcagtggaaactcattgctctatgcgttattttaagcgttgtatgtgccctactctatttacgtgtaacgccagatacctattcggtagatgctttggttcaggttgaagacagtaaaggggcttctgctgcacttttaggtgatctctcacaaatgattgagcaaaaatcaccagctcaagcagaaatagaaattttaaaatctcgtttggttttaggttctgtgattaaagatttacatctgaatatacaggtctctagcactgagaatacatttacacatcgcttattgagtgatactgattataaaactgaatatgctaaaaaatcggttttatttaaagatggtttaaaaagttttgatatacgtcagtttgagattccagcttattatctagataagaacttacttcttgactttgataaacagtctttacgtttaacagacccagatactgaagaagtcatcttaactgttccattaaaccaagctaacagcgttacaggaccgtatggtttatggaaagttgctatatttacaaaagaccaatttgactcaacttataatattaagaaattatcacttcctttagctataaaatctatcagttccgactattctgtagaagaaaaaggtaaactcacaggtatattaggacttagctatcaaggccaagatcaagaacatattactaaagtcttaaatgctattttagcgacttatagtgcacaaaatattga

>KL2-AB_908_12[AMHV00000000]

ttttcattgattgctcagtggaaactcattgctctatgcgttattttaagcgttgtatgtgccctactctatttacgtgtaacgccagatacctattcggtagatgctttggttcaggttgaagacagtaaaggggcttctgctgcacttttaggtgatctctcacaaatgattgagcaaaaatcaccagctcaagcagaaatagaaattttaaaatctcgtttggttttaggttctgtgattaaagatttacatctgaatatacaggtctctagcactgagaatacatttacacatcgcttattgagtgatactgattataaaactgaatatgctaaaaaatcggttttatttaaagatggtttaaaaagttttgatatacgtcagtttgagattccagcttattatctagataagaacttacttcttgactttgataaacagtctttacgtttaacagacccagatactgaagaagtcatcttaactgttccattaaaccaagctaacagcgttacaggaccgtatggtttatggaaagttgctatatttacaaaagaccaatttgactcaacttataatattaagaaattatcacttcctttagctataaaatctatcagttccgactattctgtagaagaaaaaggtaaactcacaggtatattaggacttagctatcaaggccaagatcaagaacatattactaaagtcttaaatgctattttagcgacttatagtgcacaaaatattga

>KL2-AB_909_05[AMIA00000000]

ttttcattgattgctcagtggaaactcattgctctatgcgttattttaagcgttgtatgtgccctactctatttacgtgtaacgccagatacctattcggtagatgctttggttcaggttgaagacagtaaaggggcttctgctgcacttttaggtgatctctcacaaatgattgagcaaaaatcaccagctcaagcagaaatagaaattttaaaatctcgtttggttttaggttctgtgattaaagatttacatctgaatatacaggtctctagcactgagaatacatttacacatcgcttattgagtgatactgattataaaactgaatatgctaaaaaatcggttttatttaaagatggtttaaaaagttttgatatacgtcagtttgagattccagcttattatctagataagaacttacttcttgactttgataaacagtctttacgtttaacagacccagatactgaagaagtcatcttaactgttccattaaaccaagctaacagcgttacaggaccgtatggtttatggaaagttgctatatttacaaaagaccaatttgactcaacttataatattaagaaattatcacttcctttagctataaaatctatcagttccgactattctgtagaagaaaaaggtaaactcacaggtatattaggacttagctatcaaggccaagatcaagaacatattactaaagtcttaaatgctattttagcgacttatagtgcacaaaatattga

>KL2-AB_909_14_7[AMIB00000000]

ttttcattgattgctcagtggaaactcattgctctatgcgttattttaagcgttgtatgtgccctactctatttacgtgtaacgccagatacctattcggtagatgctttggttcaggttgaagacagtaaaggggcttctgctgcacttttaggtgatctctcacaaatgattgagcaaaaatcaccagctcaagcagaaatagaaattttaaaatctcgtttggttttaggttctgtgattaaagatttacatctgaatatacaggtctctagcactgagaatacatttacacatcgcttattgagtgatactgattataaaactgaatatgctaaaaaatcggttttatttaaagatggtttaaaaagttttgatatacgtcagtttgagattccagcttattatctagataagaacttacttcttgactttgataaacagtctttacgtttaacagacccagatactgaagaagtcatcttaactgttccattaaaccaagctaacagcgttacaggaccgtatggtttatggaaagttgctatatttacaaaagaccaatttgactcaacttataatattaagaaattatcacttcctttagctataaaatctatcagttccgactattctgtagaagaaaaaggtaaactcacaggtatattaggacttagctatcaaggccaagatcaagaacatattactaaagtcttaaatgctattttagcgacttatagtgcacaaaatattga

>KL2-AB_TG2026[AMIH00000000]

ttttcattgattgctcagtggaaactcattgctctatgcgttattttaagcgttgtatgtgccctactctatttacgtgtaacgccagatacctattcggtagatgctttggttcaggttgaagacagtaaaggggcttctgctgcacttttaggtgatctctcacaaatgattgagcaaaaatcaccagctcaagcagaaatagaaattttaaaatctcgtttggttttaggttctgtgattaaagatttacatctgaatatacaggtctctagcactgagaatacatttacacatcgcttattgagtgatactgattataaaactgaatatgctaaaaaatcggttttatttaaagatggtttaaaaagttttgatatacgtcagtttgagattccagcttattatctagataagaacttacttcttgactttgataaacagtctttacgtttaacagacccagatactgaagaagtcatcttaactgttccattaaaccaagctaacagcgttacaggaccgtatggtttatggaaagttgctatatttacaaaagaccaatttgactcaacttataatattaagaaattatcacttcctttagctataaaatctatcagttccgactattctgtagaagaaaaaggtaaactcacaggtatattaggacttagctatcaaggccaagatcaagaacatattactaaagtcttaaatgctattttagcgacttatagtgcacaaaatattga

>KL2-AB_TG2631[AMIM00000000]

ttttcattgattgctcagtggaaactcattgctctatgcgttattttaagcgttgtatgtgccctactctatttacgtgtaacgccagatacctattcggtagatgctttggttcaggttgaagacagtaaaggggcttctgctgcacttttaggtgatctctcacaaatgattgagcaaaaatcaccagctcaagcagaaatagaaattttaaaatctcgtttggttttaggttctgtgattaaagatttacatctgaatatacaggtctctagcactgagaatacatttacacatcgcttattgagtgatactgattataaaactgaatatgctaaaaaatcggttttatttaaagatggtttaaaaagttttgatatacgtcagtttgagattccagcttattatctagataagaacttacttcttgactttgataaacagtctttacgtttaacagacccagatactgaagaagtcatcttaactgttccattaaaccaagctaacagcgttacaggaccgtatggtttatggaaagttgctatatttacaaaagaccaatttgactcaacttataatattaagaaattatcacttcctttagctataaaatctatcagttccgactattctgtagaagaaaaaggtaaactcacaggtatattaggacttagctatcaaggccaagatcaagaacatattactaaagtcttaaatgctattttagcgacttatagtgcacaaaatattga

>KL2-AB_TG27323[AMIN00000000]

ttttcattgattgctcagtggaaactcattgctctatgcgttattttaagcgttgtatgtgccctactctatttacgtgtaacgccagatacctattcggtagatgctttggttcaggttgaagacagtaaaggggcttctgctgcacttttaggtgatctctcacaaatgattgagcaaaaatcaccagctcaagcagaaatagaaattttaaaatctcgtttggttttaggttctgtgattaaagatttacatctgaatatacaggtctctagcactgagaatacatttacacatcgcttattgagtgatactgattataaaactgaatatgctaaaaaatcggttttatttaaagatggtttaaaaagttttgatatacgtcagtttgagattccagcttattatctagataagaacttacttcttgactttgataaacagtctttacgtttaacagacccagatactgaagaagtcatcttaactgttccattaaaccaagctaacagcgttacaggaccgtatggtttatggaaagttgctatatttacaaaagaccaatttgactcaacttataatattaagaaattatcacttcctttagctataaaatctatcagttccgactattctgtagaagaaaaaggtaaactcacaggtatattaggacttagctatcaaggccaagatcaagaacatattactaaagtcttaaatgctattttagcgacttatagtgcacaaaatattga

>KL2-AB_TG27327[AMIO00000000]

ttttcattgattgctcagtggaaactcattgctctatgcgttattttaagcgttgtatgtgccctactctatttacgtgtaacgccagatacctattcggtagatgctttggttcaggttgaagacagtaaaggggcttctgctgcacttttaggtgatctctcacaaatgattgagcaaaaatcaccagctcaagcagaaatagaaattttaaaatctcgtttggttttaggttctgtgattaaagatttacatctgaatatacaggtctctagcactgagaatacatttacacatcgcttattgagtgatactgattataaaactgaatatgctaaaaaatcggttttatttaaagatggtttaaaaagttttgatatacgtcagtttgagattccagcttattatctagataagaacttacttcttgactttgataaacagtctttacgtttaacagacccagatactgaagaagtcatcttaactgttccattaaaccaagctaacagcgttacaggaccgtatggtttatggaaagttgctatatttacaaaagaccaatttgactcaacttataatattaagaaattatcacttcctttagctataaaatctatcagttccgactattctgtagaagaaaaaggtaaactcacaggtatattaggacttagctatcaaggccaagatcaagaacatattactaaagtcttaaatgctattttagcgacttatagtgcacaaaatattga

>KL2-AB_TG27331[AMIP00000000]

ttttcattgattgctcagtggaaactcattgctctatgcgttattttaagcgttgtatgtgccctactctatttacgtgtaacgccagatacctattcggtagatgctttggttcaggttgaagacagtaaaggggcttctgctgcacttttaggtgatctctcacaaatgattgagcaaaaatcaccagctcaagcagaaatagaaattttaaaatctcgtttggttttaggttctgtgattaaagatttacatctgaatatacaggtctctagcactgagaatacatttacacatcgcttattgagtgatactgattataaaactgaatatgctaaaaaatcggttttatttaaagatggtttaaaaagttttgatatacgtcagtttgagattccagcttattatctagataagaacttacttcttgactttgataaacagtctttacgtttaacagacccagatactgaagaagtcatcttaactgttccattaaaccaagctaacagcgttacaggaccgtatggtttatggaaagttgctatatttacaaaagaccaatttgactcaacttataatattaagaaattatcacttcctttagctataaaatctatcagttccgactattctgtagaagaaaaaggtaaactcacaggtatattaggacttagctatcaaggccaagatcaagaacatattactaaagtcttaaatgctattttagcgacttatagtgcacaaaatattga

>KL2-Naval_113[AMZU00000000]

ttttcattgattgctcagtggaaactcattgctctatgcgttattttaagcgttgtatgtgccctactctatttacgtgtaacgccagatacctattcggtagatgctttggttcaggttgaagacagtaaaggggcttctgctgcacttttaggtgatctctcacaaatgattgagcaaaaatcaccagctcaagcagaaatagaaattttaaaatctcgtttggttttaggttctgtgattaaagatttacatctgaatatacaggtctctagcactgagaatacatttacacatcgcttattgagtgatactgattataaaactgaatatgctaaaaaatcggttttatttaaagatggtttaaaaagttttgatatacgtcagtttgagattccagcttattatctagataagaacttacttcttgactttgataaacagtctttacgtttaacagacccagatactgaagaagtcatcttaactgttccattaaaccaagctaacagcgttacaggaccgtatggtttatggaaagttgctatatttacaaaagaccaatttgactcaacttataatattaagaaattatcacttcctttagctataaaatctatcagttccgactattctgtagaagaaaaaggtaaactcacaggtatattaggacttagctatcaaggccaagatcaagaacatattactaaagtcttaaatgctattttagcgacttatagtgcacaaaatattga

>KL2-ACICU[CP000863]

ttttcattgattgctcagtggaaactcattgctctatgcgttattttaagcgttgtatgtgccctactctatttacgtgtaacgccagatacctattcggtagatgctttggttcaggttgaagacagtaaaggggcttctgctgcacttttaggtgatctctcacaaatgattgagcaaaaatcaccagctcaagcagaaatagaaattttaaaatctcgtttggttttaggttctgtgattaaagatttacatctgaatatacaggtctctagcactgagaatacatttacacatcgcttattgagtgatactgattataaaactgaatatgctaaaaaatcggttttatttaaagatggtttaaaaagttttgatatacgtcagtttgagattccagcttattatctagataagaacttacttcttgactttgataaacagtctttacgtttaacagacccagatactgaagaagtcatcttaactgttccattaaaccaagctaacagcgttacaggaccgtatggtttatggaaagttgctatatttacaaaagaccaatttgactcaacttataatattaagaaattatcacttcctttagctataaaatctatcagttccgactattctgtagaagaaaaaggtaaactcacaggtatattaggacttagctatcaaggccaagatcaagaacatattactaaagtcttaaatgctattttagcgacttatagtgcacaaaatattga

>KL2-OIFC189[AFDM00000000]

ttttcattgattgctcagtggaaactcattgctctatgcgttattttaagcgttgtatgtgccctactctatttacgtgtaacgccagatacctattcggtagatgctttggttcaggttgaagacagtaaaggggcttctgctgcacttttaggtgatctctcacaaatgattgagcaaaaatcaccagctcaagcagaaatagaaattttaaaatctcgtttggttttaggttctgtgattaaagatttacatctgaatatacaggtctctagcactgagaatacatttacacatcgcttattgagtgatactgattataaaactgaatatgctaaaaaatcggttttatttaaagatggtttaaaaagttttgatatacgtcagtttgagattccagcttattatctagataagaacttacttcttgactttgataaacagtctttacgtttaacagacccagatactgaagaagtcatcttaactgttccattaaaccaagctaacagcgttacaggaccgtatggtttatggaaagttgctatatttacaaaagaccaatttgactcaacttataatattaagaaattatcacttcctttagctataaaatctatcagttccgactattctgtagaagaaaaaggtaaactcacaggtatattaggacttagctatcaaggccaagatcaagaacatattactaaagtcttaaatgctattttagcgacttatagtgcacaaaatattga

>LUH5542_[KC526901]

ttttcactgattgcacagtggaaactaatcgcactctgcattattttaagtcttgtatgcgcactactttatttacgtgtaacaccagatacctactcggttgatgctttggttcaagttgaagacagcaaaggtgcttctgctgcacttttgggcgatttgtccgatatgatcgaacaaaagtcaccagcgcaagctgaaattgaaattttaaagtctcggttggttttaggctcggttattaaagaactacatctagatattcacgtttctagcaccgaaaacacacttactcaccgtttattaagtgatactgaatataaaactgaatacactaaaaattcagttttatttaaagatggcaccaaaaattttgatatacgaagatttgaagtacctgcatattatttagacaaacaacttgtattaagctttgatcaaaaatcttttaaattagtagatcctgagacggatgaagttgtactcacagctccaatcaatcaaaataaccaaattacaggagcttatggcgtatggaaggttgcgatctttactaaagatcaattagattcaaaatataatattaagaaactctctattccagcagctgtagataatataagttcaaattattcagtagccgaacgcggtaaattaacaggtattcttggtttaaattatcaaggatatgataaagaacatattactcaagtcttaaatgcgatcttggtaacatatggtgcccaaaatgttga

>KL1-AB307-0294[CP001172]

ttttcactgattgcacagtggaaactaatcgcactctgcattattttaagtcttgtatgcgcactactttatttacgtgtaacaccagatacctactcggttgatgctttggttcaagttgaagacagcaaaggtgcttctgctgcacttttgggcgatttgtccgatatgatcgaacaaaagtcaccagcgcaagctgaaattgaaattttaaagtctcggttggttttaggctcggttattaaagaactacatctagatattcacgtttctagcaccgaaaacacacttactcaccgtttattaagtgatactgaatataaaactgaatacactaaaaattcagttttatttaaagatggcaccaaaaattttgatatacgaagatttgaagtacctgcatattatttagacaaacaacttgtattaagctttgatcaaaaatcttttaaattagtagatcctgagacggatgaagttgtactcacagctccaatcaatcaaaataaccaaattacaggagcttatggcgtatggaaggttgcgatctttactaaagatcaattagattcaaaatataatattaagaaactctctattccagcagctgtagataatataagttcaaattattcagtagccgaacgcggtaaattaacaggtattcttggtttaaattatcaaggatatgataaagaacatattactcaagtcttaaatgcgatcttggtaacatatggtgcccaaaatgttga

>KL1-AYE[CU459141]

ttttcactgattgcacagtggaaactaatcgcactctgcattattttaagtcttgtatgcgcactactttatttacgtgtaacaccagatacctactcggttgatgctttggttcaagttgaagacagcaaaggtgcttctgctgcacttttgggcgatttgtccgatatgatcgaacaaaagtcaccagcgcaagctgaaattgaaattttaaagtctcggttggttttaggctcggttattaaagaactacatctagatattcacgtttctagcaccgaaaacacacttactcaccgtttattaagtgatactgaatataaaactgaatacactaaaaattcagttttatttaaagatggcaccaaaaattttgatatacgaagatttgaagtacctgcatattatttagacaaacaacttgtattaagctttgatcaaaaatcttttaaattagtagatcctgagacggatgaagttgtactcacagctccaatcaatcaaaataaccaaattacaggagcttatggcgtatggaaggttgcgatctttactaaagatcaattagattcaaaatataatattaagaaactctctattccagcagctgtagataatataagttcaaattattcagtagccgaacgcggtaaattaacaggtattcttggtttaaattatcaaggatatgataaagaacatattactcaagtcttaaatgcgatcttggtaacatatggtgcccaaaatgttga

>KL1-A1[CP010781.1]

ttttcactgattgcacagtggaaactaatcgcactctgcattattttaagtcttgtatgcgcactactttatttacgtgtaacaccagatacctactcggttgatgctttggttcaagttgaagacagcaaaggtgcttctgctgcacttttgggcgatttgtccgatatgatcgaacaaaagtcaccagcgcaagctgaaattgaaattttaaagtctcggttggttttaggctcggttattaaagaactacatctagatattcacgtttctagcaccgaaaacacacttactcaccgtttattaagtgatactgaatataaaactgaatacactaaaaattcagttttatttaaagatggcaccaaaaattttgatatacgaagatttgaagtacctgcatattatttagacaaacaacttgtattaagctttgatcaaaaatcttttaaattagtagatcctgagacggatgaagttgtactcacagctccaatcaatcaaaataaccaaattacaggagcttatggcgtatggaaggttgcgatctttactaaagatcaattagattcaaaatataatattaagaaactctctattccagcagctgtagataatataagttcaaattattcagtagccgaacgcggtaaattaacaggtattcttggtttaaattatcaaggatatgataaagaacatattactcaagtcttaaatgcgatcttggtaacatatggtgcccaaaatgttga

>LUH5543_[KC526913]

ttttcattgattgctcagtggaaactcattgctctatgcgttattttaagcgttgtatgtgctctactctatttacgtgtaacaccagatacctattcagtagatgctttggttcaggttgaagatagtaaaggtgcttctgctgcgcttttaggtgatctatcacaaatgatcgagcaaaaatcacccgctcaagcagaaatagaaattttaaaatctcgtttagtcctaggatctgtcattaaagatttacacctgaatatacaggtgtctagtactgaaaatacacttacccatcgtttattaagtgataccgaatataaaactgaatacactaaaaaatcagttttatttaaagataacttaaaaagttttgaagtgcgtgaatttgaagttccagcttactatctagacaaaaacttacttcttaattttgataaacagtctttacgtttaacagacccagatactgaagaagttatcttaactgttccattaaaccaagttaatcacgttgcagggcctcatggtttatggaaaattgccatctttactaaagatcaatttgacgcgacctataatattactaatttgtctttacctgctgctgtaaatgctcttagcgtaaattattctgtcgcagaacgaggcaaacttacaggtgttttaggcctaaattatcaaggccaagataaagaacatattactaaagttttaaatgcaattctggcaacttatagtgcacaaaatattga

>KL43-LUH5544[KC526905]

ttttcattgattgcacagtggaaaatcatagcatgctgcgtggtcttaagtttaatttgtgccttgctatacttacggataacgccagatacttattctgtagatgcaatggtacaagttgaggacagtaaaggcgctgcctctgctgctttgcttggtgacctttctaaagtcagtggaggtctatctcaaaaatcaccagctgatccagaaatagagattcttcgttctagaatggttttaggacaagtcattcagaaccttaatcttgatattaacgtcaaagataaccaatcaggattgatcgacaagttaatttcacaagataagagtagacttgagtatcgtcatgaatctgtactgtacagtaatcaaaataataatttgattatacgagaacttaaagtacctgaatattatttagataaacccttaaagttagagttcaaaggtgcaaatcaattcactttaacatacaaagatcaagttgtatttaatggtcaattaaacaaaaaaaatatactaaatacaaatagaggtctttggcaggtacaaattaatgctcaaggtaacctgaaagaacaaagctatactttaactaaactcgctctaccaacagccgttaaaaattttaatgatatctatagtgttgctgaaaaaggtaaagtgactggtgttattggtttaaattatctaggtcaagatcctgagcacattacacaagtactaaataatgttctaactgtttatcaccaacaaaacattga-

>-LUH5545_[KC526904]

ttttcattgattgcccaatggaaattgattgtattatgtattcttataagtctagtctgtgcattaatttatttacgtgtcactccaaatacttattcggttgatgcattggttcaggtagaagatactaaaagtgcagcttcggcagctttattaggtgaactatctaaaatggttgaccaaaaatctccagcagaggctgaaatacaagttcttacctctcgtatggtgctcggacaggttatccataatttaaatctcgatatcacaattgaaaatcatgatgatactttcttcaatcgtcttttaagacaagatcaacagaacattgattaccagaaagattctgtaacctttagtaataaagatagctatttctctatccaaaaacttcaagtacctgcatattatcttgataaaccccttttgttatcttttaaggatcaaggacattttacttttagctataaagataaaattatttttaatggtcaactcaatgctaataacatcattactactcgtgaaggacaatggaaaataagaatcaattctaagcgctctccatctagcgaacagcactttacaattagcaaacttgcgttaccaactgcagtacaaaaacttggctctacttatgctgtagctgaaaaaggcaaacaaactggagttatcggtttaagtttccaaggtacagataaagaacatataactgaagttttaaataatattttagctgtatatcatagccaaaatattga

>-AB900[ABXK00000000]

ttttcattgattgcccaatggaaattgattgtattatgtattcttataagtctagtctgtgcattaatttatttacgtgtcactccaaatacttattcggttgatgcattggttcaggtagaagatactaaaagtgcagcttcggcagctttattaggtgaactatctaaaatggttgaccaaaaatctccagcagaggctgaaatacaagttcttacctctcgtatggtgctcggacaggttatccataatttaaatctcgatatcacaattgaaaatcatgatgatactttcttcaatcgtcttttaagacaagatcaacagaacattgattaccagaaagattctgtaacctttagtaataaagatagctatttctctatccaaaaacttcaagtacctgcatattatcttgataaaccccttttgttatcttttaaggatcaaggacattttacttttagctataaagataaaattatttttaatggtcaactcaatgctaataacatcattactactcgtgaaggacaatggaaaataagaatcaattctaagcgctctccatctagcgaacagcactttacaattagcaaacttgcgttaccaactgcagtacaaaaacttggctctacttatgctgtagctgaaaaaggcaaacaaactggagttatcggtttaagtttccaaggtacagataaagaacatataactgaagttttaaataatattttagctgtatatcatagccaaaatattga

>-J9[KF002790.2]

ttttcattgattgcccaatggaaattgattgtattatgtattcttataagtctagtctgtgcattaatttatttacgtgtcactccaaatacttattcggttgatgcattggttcaggtagaagatactaaaagtgcagcttcggcagctttattaggtgaactatctaaaatggttgaccaaaaatctccagcagaggctgaaatacaagttcttacctctcgtatggtgctcggacaggttatccataatttaaatctcgatatcacaattgaaaatcatgatgatactttcttcaatcgtcttttaagacaagatcaacagaacattgattaccagaaagattctgtaacctttagtaataaagatagctatttctctatccaaaaacttcaagtacctgcatattatcttgataaaccccttttgttatcttttaaggatcaaggacattttacttttagctataaagataaaattatttttaatggtcaactcaatgctaataacatcattactactcgtgaaggacaatggaaaataagaatcaattctaagcgctctccatctagcgaacagcactttacaattagcaaacttgcgttaccaactgcagtacaaaaacttggctctacttatgctgtagctgaaaaaggcaaacaaactggagttatcggtttaagtttccaaggtacagataaagaacatataactgaagttttaaataatattttagctgtatatcatagccaaaatattga

>-OIFC111[AMFY00000000]

ttttcattgattgcccaatggaaattgattgtattatgtattcttataagtctagtctgtgcattaatttatttacgtgtcactccaaatacttattcggttgatgcattggttcaggtagaagatactaaaagtgcagcttcggcagctttattaggtgaactatctaaaatggttgaccaaaaatctccagcagaggctgaaatacaagttcttacctctcgtatggtgctcggacaggttatccataatttaaatctcgatatcacaattgaaaatcatgatgatactttcttcaatcgtcttttaagacaagatcaacagaacattgattaccagaaagattctgtaacctttagtaataaagatagctatttctctatccaaaaacttcaagtacctgcatattatcttgataaaccccttttgttatcttttaaggatcaaggacattttacttttagctataaagataaaattatttttaatggtcaactcaatgctaataacatcattactactcgtgaaggacaatggaaaataagaatcaattctaagcgctctccatctagcgaacagcactttacaattagcaaacttgcgttaccaactgcagtacaaaaacttggctctacttatgctgtagctgaaaaaggcaaacaaactggagttatcggtttaagtttccaaggtacagataaagaacatataactgaagttttaaataatattttagctgtatatcatagccaaaatattga

>-AB_2007_16_25_01_7[AMHI00000000]

ttttcattgattgcccaatggaaattgattgtattatgtattcttataagtctagtctgtgcattaatttatttacgtgtcactccaaatacttattcggttgatgcattggttcaggtagaagatactaaaagtgcagcttcggcagctttattaggtgaactatctaaaatggttgaccaaaaatctccagcagaggctgaaatacaagttcttacctctcgtatggtgctcggacaggttatccataatttaaatctcgatatcacaattgaaaatcatgatgatactttcttcaatcgtcttttaagacaagatcaacagaacattgattaccagaaagattctgtaacctttagtaataaagatagctatttctctatccaaaaacttcaagtacctgcatattatcttgataaaccccttttgttatcttttaaggatcaaggacattttacttttagctataaagataaaattatttttaatggtcaactcaatgctaataacatcattactactcgtgaaggacaatggaaaataagaatcaattctaagcgctctccatctagcgaacagcactttacaattagcaaacttgcgttaccaactgcagtacaaaaacttggctctacttatgctgtagctgaaaaaggcaaacaaactggagttatcggtttaagtttccaaggtacagataaagaacatataactgaagttttaaataatattttagctgtatatcatagccaaaatattga

>-AB_2007_16_27_01[AMHJ00000000]

ttttcattgattgcccaatggaaattgattgtattatgtattcttataagtctagtctgtgcattaatttatttacgtgtcactccaaatacttattcggttgatgcattggttcaggtagaagatactaaaagtgcagcttcggcagctttattaggtgaactatctaaaatggttgaccaaaaatctccagcagaggctgaaatacaagttcttacctctcgtatggtgctcggacaggttatccataatttaaatctcgatatcacaattgaaaatcatgatgatactttcttcaatcgtcttttaagacaagatcaacagaacattgattaccagaaagattctgtaacctttagtaataaagatagctatttctctatccaaaaacttcaagtacctgcatattatcttgataaaccccttttgttatcttttaaggatcaaggacattttacttttagctataaagataaaattatttttaatggtcaactcaatgctaataacatcattactactcgtgaaggacaatggaaaataagaatcaattctaagcgctctccatctagcgaacagcactttacaattagcaaacttgcgttaccaactgcagtacaaaaacttggctctacttatgctgtagctgaaaaaggcaaacaaactggagttatcggtttaagtttccaaggtacagataaagaacatataactgaagttttaaataatattttagctgtatatcatagccaaaatattga

>-AB_TG27339[AMIR00000000]

ttttcattgattgcccaatggaaattgattgtattatgtattcttataagtctagtctgtgcattaatttatttacgtgtcactccaaatacttattcggttgatgcattggttcaggtagaagatactaaaagtgcagcttcggcagctttattaggtgaactatctaaaatggttgaccaaaaatctccagcagaggctgaaatacaagttcttacctctcgtatggtgctcggacaggttatccataatttaaatctcgatatcacaattgaaaatcatgatgatactttcttcaatcgtcttttaagacaagatcaacagaacattgattaccagaaagattctgtaacctttagtaataaagatagctatttctctatccaaaaacttcaagtacctgcatattatcttgataaaccccttttgttatcttttaaggatcaaggacattttacttttagctataaagataaaattatttttaatggtcaactcaatgctaataacatcattactactcgtgaaggacaatggaaaataagaatcaattctaagcgctctccatctagcgaacagcactttacaattagcaaacttgcgttaccaactgcagtacaaaaacttggctctacttatgctgtagctgaaaaaggcaaacaaactggagttatcggtttaagtttccaaggtacagataaagaacatataactgaagttttaaataatattttagctgtatatcatagccaaaatattga

>-3.5D_[ NZ_MABZ00000000.1]

ttttcattgattgcccaatggaaattgattgtattatgtattcttataagtctagtctgtgcattaatttatttacgtgtcactccaaatacttattcggttgatgcattggttcaggtagaagatactaaaagtgcagcttcggcagctttattaggtgaactatctaaaatggttgaccaaaaatctccagcagaggctgaaatacaagttcttacctctcgtatggtgctcggacaggttatccataatttaaatctcgatatcacaattgaaaatcatgatgatactttcttcaatcgtcttttaagacaagatcaacagaacattgattaccagaaagattctgtaacctttagtaataaagatagctatttctctatccaaaaacttcaagtacctgcatattatcttgataaaccccttttgttatcttttaaggatcaaggacattttacttttagctataaagataaaattatttttaatggtcaactcaatgctaataacatcattactactcgtgaaggacaatggaaaataagaatcaattctaagcgctctccatctagcgaacagcactttacaattagcaaacttgcgttaccaactgcagtacaaaaacttggctctacttatgctgtagctgaaaaaggcaaacaaactggagttatcggtttaagtttccaaggtacagataaagaacatataactgaagttttaaataatattttagctgtatatcatagccaaaatattga

>-3207_[ CP015364.1]

ttttcattgattgcccaatggaaattgattgtattatgtattcttataagtctagtctgtgcattaatttatttacgtgtcactccaaatacttattcggttgatgcattggttcaggtagaagatactaaaagtgcagcttcggcagctttattaggtgaactatctaaaatggttgaccaaaaatctccagcagaggctgaaatacaagttcttacctctcgtatggtgctcggacaggttatccataatttaaatctcgatatcacaattgaaaatcatgatgatactttcttcaatcgtcttttaagacaagatcaacagaacattgattaccagaaagattctgtaacctttagtaataaagatagctatttctctatccaaaaacttcaagtacctgcatattatcttgataaaccccttttgttatcttttaaggatcaaggacattttacttttagctataaagataaaattatttttaatggtcaactcaatgctaataacatcattactactcgtgaaggacaatggaaaataagaatcaattctaagcgctctccatctagcgaacagcactttacaattagcaaacttgcgttaccaactgcagtacaaaaacttggctctacttatgctgtagctgaaaaaggcaaacaaactggagttatcggtttaagtttccaaggtacagataaagaacatataactgaagttttaaataatattttagctgtatatcatagccaaaatattga

>-1293320_[ NZ_JFEE00000000.1]

ttttcattgattgcccaatggaaattgattgtattatgtattcttataagtctagtctgtgcattaatttatttacgtgtcactccaaatacttattcggttgatgcattggttcaggtagaagatactaaaagtgcagcttcggcagctttattaggtgaactatctaaaatggttgaccaaaaatctccagcagaggctgaaatacaagttcttacctctcgtatggtgctcggacaggttatccataatttaaatctcgatatcacaattgaaaatcatgatgatactttcttcaatcgtcttttaagacaagatcaacagaacattgattaccagaaagattctgtaacctttagtaataaagatagctatttctctatccaaaaacttcaagtacctgcatattatcttgataaaccccttttgttatcttttaaggatcaaggacattttacttttagctataaagataaaattatttttaatggtcaactcaatgctaataacatcattactactcgtgaaggacaatggaaaataagaatcaattctaagcgctctccatctagcgaacagcactttacaattagcaaacttgcgttaccaactgcagtacaaaaacttggctctacttatgctgtagctgaaaaaggcaaacaaactggagttatcggtttaagtttccaaggtacagataaagaacatataactgaagttttaaataatattttagctgtatatcatagccaaaatattga

>-1461963_[ NZ_JEWQ00000000.1]

ttttcattgattgcccaatggaaattgattgtattatgtattcttataagtctagtctgtgcattaatttatttacgtgtcactccaaatacttattcggttgatgcattggttcaggtagaagatactaaaagtgcagcttcggcagctttattaggtgaactatctaaaatggttgaccaaaaatctccagcagaggctgaaatacaagttcttacctctcgtatggtgctcggacaggttatccataatttaaatctcgatatcacaattgaaaatcatgatgatactttcttcaatcgtcttttaagacaagatcaacagaacattgattaccagaaagattctgtaacctttagtaataaagatagctatttctctatccaaaaacttcaagtacctgcatattatcttgataaaccccttttgttatcttttaaggatcaaggacattttacttttagctataaagataaaattatttttaatggtcaactcaatgctaataacatcattactactcgtgaaggacaatggaaaataagaatcaattctaagcgctctccatctagcgaacagcactttacaattagcaaacttgcgttaccaactgcagtacaaaaacttggctctacttatgctgtagctgaaaaaggcaaacaaactggagttatcggtttaagtttccaaggtacagataaagaacatataactgaagttttaaataatattttagctgtatatcatagccaaaatattga

>-ABBL038_[ NZ_LLDS00000000.1]

ttttcattgattgcccaatggaaattgattgtattatgtattcttataagtctagtctgtgcattaatttatttacgtgtcactccaaatacttattcggttgatgcattggttcaggtagaagatactaaaagtgcagcttcggcagctttattaggtgaactatctaaaatggttgaccaaaaatctccagcagaggctgaaatacaagttcttacctctcgtatggtgctcggacaggttatccataatttaaatctcgatatcacaattgaaaatcatgatgatactttcttcaatcgtcttttaagacaagatcaacagaacattgattaccagaaagattctgtaacctttagtaataaagatagctatttctctatccaaaaacttcaagtacctgcatattatcttgataaaccccttttgttatcttttaaggatcaaggacattttacttttagctataaagataaaattatttttaatggtcaactcaatgctaataacatcattactactcgtgaaggacaatggaaaataagaatcaattctaagcgctctccatctagcgaacagcactttacaattagcaaacttgcgttaccaactgcagtacaaaaacttggctctacttatgctgtagctgaaaaaggcaaacaaactggagttatcggtttaagtttccaaggtacagataaagaacatataactgaagttttaaataatattttagctgtatatcatagccaaaatattga

>-ABBL071_[ NZ_LLGB00000000.1]

ttttcattgattgcccaatggaaattgattgtattatgtattcttataagtctagtctgtgcattaatttatttacgtgtcactccaaatacttattcggttgatgcattggttcaggtagaagatactaaaagtgcagcttcggcagctttattaggtgaactatctaaaatggttgaccaaaaatctccagcagaggctgaaatacaagttcttacctctcgtatggtgctcggacaggttatccataatttaaatctcgatatcacaattgaaaatcatgatgatactttcttcaatcgtcttttaagacaagatcaacagaacattgattaccagaaagattctgtaacctttagtaataaagatagctatttctctatccaaaaacttcaagtacctgcatattatcttgataaaccccttttgttatcttttaaggatcaaggacattttacttttagctataaagataaaattatttttaatggtcaactcaatgctaataacatcattactactcgtgaaggacaatggaaaataagaatcaattctaagcgctctccatctagcgaacagcactttacaattagcaaacttgcgttaccaactgcagtacaaaaacttggctctacttatgctgtagctgaaaaaggcaaacaaactggagttatcggtttaagtttccaaggtacagataaagaacatataactgaagttttaaataatattttagctgtatatcatagccaaaatattga

>LUH5546_[KC526899]

ttttcattgattgcacagtggaaaatcatagcatgctgcgtggtcttaagtttaatttgtgccttgctatacttacggataacgccagatacttattctgtagatgcaatggtacaagttgaggacagtaaaggcgctgcctctgctgctttgcttggtgacctttctaaagtcagtggaggtctatctcaaaaatcaccagctgatccagaaatagagattcttcgttctagaatggttttaggacaagtcattcagaaccttaatcttgatattaacgtcaaagataaccaatcaggattgatcgacaagttaatttcacaagataagagtagacttgagtatcgtcatgaatctgtactgtacagtaatcaaaataataatttgattatacgagaacttaaagtacctgaatattatttagataaacccttaaagttagagttcaaaggtgcaaatcaattcactttaacatacaaagatcaagttgtatttaatggtcaattaaacaaaaaaaatatactaaatacaaatagaggtctttggcaggtacaacttaatgctcaaggtaacctgaaagaacaaagctatactttaactaaactcgctctaccaacagccgttaaaaattttaatgatatctatagtgttgctgaaaaaggtaaagtgactggtgttattggtttaaattatctaggtcaagatcctgagcacattacacaagtactaaataatgttctaactgtttatcaccaacaaaacattga

>KL87a-LUH5547[KC526918]

ttctcactgattgctcagtggaagctgattgcgctctgtattattttgagccttgtgtgtgctctactctatttacgtgtaacaccagatacctactcggttgatgctttggttcaagttgaagacagtaaaggagcctctgctgccctgctaggtgatttatcgcagatgattgagcaaaaatcaccagctcaagctgagattgaaattttaaaatcacgtttggttttaggttctgtcattaaagacttacatctaaatatacaaatttctagtaccgaaaatacgcttactcatcgtttattaagtgatcctgaatataaaactgagtacactaaaaaatcagttatctttaaagatgacttaaaaagttttgaagtgcgtgaatttgaagttccgaattattatctagacaaaaacttacttcttaattttgataaacaatctttaactttaactgacccagaaactgaagaggtaatattaacagttccgttgaaccaagctaatcatgttactgggccacatggactctggaaggttgcaatttttactaaagatcaactcgatgcgacctataatattactaatttgtctttacctgctgctgtaaatgcccttagcgtaaattattctgtcgcagaacgaggcaaacttacaggtgttttaggcctaaattatcaaggccaagataaagaacatattactaaagttttaaatgcaattctggcaacttatagtgcacaaaatattga

>KL88-LUH5548[KC526910]

ttttcattgattgcccaatggaaattgattgtactatgtattcttataagtctagtctgtgcattaatttatttacgtgtcactccaaatacttattcggttgatgcattggttcaggtcgaagatactaaaagtgcagcttcagcagctttattaggtgaactatctaaaatggttgaccaaaaatctccagcagaggctgaaatacaggttcttacctctcgtatggtgctcggtcaggttatccacaatttaaatctagatattacaattaaaaatcatgatgatactttcttcaaccgtcttttaagtcaagataagcagaatattgattataagaaagatgctgtaacttttagtaataaagatagttatttctctatccaacagttacagattccttcgtattatcttgataaaccccttttactttcttttaaagatcaggggcatttcactttcagctataaagataaaattatttttagtggtcagctcaatagtaataatttagtaactgctcgtgaaggacaatggaaagtacgaattaattctacacatgctccatctgtagagcagcaatttacaatcagtaaacttgctttaccaactgccgtacaaaaactcggctctacttacggggtagctgaacgaggcaaacaaacgggagttatcggtttaagttttcaaggaactgataaagaacacatcactgaagtcttaaataatattttagctgtatatcatagccaaaatattga

>BAL_058_KL32[KT359615.1]

ttttcactgattgctcagtggaaactcattgctctatgcgttattttaagcgttgtatgtgccctactctatttacgtgtaacgccagatacctattcggtagatgctttagttcaggttgaggacagtaaaggggcttctgctgcacttttaggtgatctatcacaaatgattgagcaaaaatcaccagctcaagcagaaatagaaattttaaaatctcgtttggttttaggttctgttattaaagatttacatcttaatatacaggtttctagcacagagaatactttcactcatcgcttattaagtaatccagaatataaaactgaatataatacgaaatcagtcatttttaaggatggtttaaaaagttttgatattcatcagtttgatattcctgcttattatctagataaaaatttacttcttaattttgataaacagtctttacgtttaaccgacccagcatctgaagaagttatcttaactgttccattaaaccaagctaatcacgttgctggtcctcatggtttatggaaagttgctatctttacaaaagatcaatttgatgcaacttataatattaaaattcaatcaataccagcagcagtagatgcacttagcgcaaattattcagtggcagaacggggtaaactaactggtattttagggctaagctaccaagggcaagatcaagaacacattaccaaagttctcaatgcgattttggcgacctatagtgcacaaaatattga

>KL42-LUH5550[KC526903]

ttttcactgattgcacagtggaaactcattgctctatgcgttattttaagtgttgtatgtgcactactctatttacgtgtaacaccagatacctattcggtagatgctttagttcaggttgaggacagcaagggagcttctgctgcacttttaggcgatctatcacaaatgatcgagcaaaaatcaccagctcaagcagaaatagaaattttaaaatctcgtttagttttaggttctgttattaaagatttacatctggatatacaggtctctagcactgaaaatacatttacacatcgcttattgagtgatactgattacaaaactgaatatgctaaaaaatcggttttatttaaagatggtttaaaaagttttgatatacgtcagtttgagattccagcttattatctagataagaacttacttcttgattttgataaacagtctttacgtttaacagatccagatactgaagaagtcatcttaactgttccattaaaccaagctaacagcgttacaggaccgtatggtgtatggaaagttgctatatttacaaacgaccaatttgaatcaacttataatattaagaaattatcacttcctttagctataaaatctatcagttccgactattctgtagaagaaaaaggtaaactcacaggtatattaggacttagctatcaagggcaagatcaagaacatattactaaagtcttaaatgctattttagcgacctatagtgcacaaaatattga

>LUH3714_[KC526911]

ttttctttgattgcacagtggaaaatcatagcatgctgtgtgattttaagtttaatttgtgccttattatatttacggataacaccagatatttatgctgtagatgcaatggtacaagtggaagatagtaaaggcgctgcctctgctgctttgcttggagacctttctaaagtcagtggaggtctatctcaaaaatcaccagctgatccagaaatagagattttaaaatctcgtatggttttagggcaagtcatccagaatcttaatcttgatattaacatcaaagataatcaatcaggattgattgataagttaatttcacaagataagagtaaaattgaatatcatcatgaatctgtactttacagtaatcaaaataataatttgattatacgagaatttaaagtacctgaatattattttgataaacctttaaaattagagtttaaaggtacaaatcaattcactttaatgtacaaagatcaagttgtatttaatggcctgctaaacaagaaaaatatactaaacacagataggggcctttggcagatacaaattaatgctcaaggtaatttgaaagaacaaagctacactttaactaagctcgctctaccaacagctgttaaaaattttaataatatttatagtgttgccgagaaaggtaaattgacgggcgttattggtttaagttatttaggacaggaccctgaacacattacccaagtacttaataatgttctatctgtctatcatgaacaaaatattga-

>LUH3712_[KC526914]

ttttctttgattgcacagtggaaaatcatagcatgctgtgtgattttaagtttaatttgtgccttattatatttacggataacaccagatatttatgctgtagatgcaatggtacaagtggaagatagtaaaggcgctgcctctgctgctttgcttggagacctttctaaagtcagtggaggtctatctcaaaaatcaccagctgatccagaaatagagattttaaaatctcgtatggttttagggcaagtcatccagaatcttaatcttgatattaacatcaaagataatcaatcaggattgattgataagttaatttcacaagataagagtaaaattgaatatcatcatgaatctgtactttacagtaatcaaaataataatttgattatacgagaatttaaagtacctgaatattattttgataaacctttaaaattagagtttaaaggtacaaatcaattcactttaatgtacaaagatcaagttgtatttaatggcctgctaaacaagaaaaatatactaaacacagataggggcctttggcagatacaaattaatgctcaaggtaatttgaaagaacaaagctacactttaactaagctcgctctaccaacagctgttaaaaattttaataatatttatagtgttgccgagaaaggtaaattgacgggcgttattggtttaagttatttaggacaggaccctgaacacattacccaagtacttaataatgttctatctgtctatcatgaacaaaatattga-

>BAL_103_KL63[KX712117.2]

ttctcactcattgcacagtggaagcttattgctctatgcattattttgagcctagtatgtgctctactctatttacgtgtaacacctgacacttattcggttgatgctttggttcaggtcgaagacagtaaaggtgcttctgctgcacttttgggtgatttgtccaatatgattgaacaaaagtcaccagctcaagcagaaattgagattttaaaatctcgtctagttttaggttcagtcgttaaagacttacatctcaatatacgagtttctagtacagaaaatacacttactcaccgtttattaagtaatagtgactataaaactgaatataataaaaaatctgtcatttttaaggatgggctcaagaattttgagattcagcaattcgatattccagcctattatctggacaaaaatttgctactcaacttcgataaacaatccttgcgtttaaccgatccagatactgagcaaaccatattgacactgccacttaatcagttaaaccaagttacaggcccacatgggacttggaaagttgctatatttaccaaagatcaatttgatgccacatataatattaaaagtctatctttacctgctgccgtcagctcaatcagtgcacaatattttgtagctgagcgcggtaaattaacaggtattcttggcttaaattatcaaggtcaagatccagaacatatcactaaagttttaaatgctattttagctacctatagtgcccaaaatattga

>LUH5552_[KC526919]

ttttcattgattgcacagtggaaaatcatagcatgctgcgtggtcttaagtttaatttgtgccttgctatacttacggataacgccagatatttatgctgtagatgcaatggtacaagttgaggacagtaaaggcgctgcctctgctgctttgcttggtgacctttcaaaagccagtggaggtctatctcaaaaatcaccagctgatccagaaatagagattttgaaatctcgtatggttttagggcaggtcatccagaatcttaaccttgatattaacattaaagataatcaatctagtttaattggtaaacttgtctcacaagaccaaagcaaactcgagtatcgtcatgatgctgtaatttacactaaccaaaacagtaatttaatagttaaacaactcagtgtgcctgaatattatttagacaaacctttaaaattagagtttaaagatgtaaatcaatttactctaacttataaagatcaagttgtatttaatggtttactaaacaagaaaaatgtactgaatacacaaaaagggttatggcaagttcagattaatacacatagaaacttgaaagatcacagctatactttaactaaactcgctctaccaacagccgttaaaaattttaatgatatctatagtgttgctgaaaaaggtaaagtgactggtgttattggtttaaattatctaggtcaagatcctgagcacattacacaagtactcaataatgttctatctgtctatcatgaacaaaatattga-

>LUH5553_[KC526917]

ttttcactgattgctcagtggaagcttattgctctatgcgttattttaagcattgtatgtgctctactctatttacgtgtaacgccagatacctattcggtagatgctttggttcaggttgaagacagtaagggtgcttctgctgcgcttttaggtgatttatcacaaatgatcgagcaaaaatcaccagctcaggcagaaatagaaattttaaaatctcgtttggttttaggttctgtgattaaagatttacatcttaatatacaggtctctagcacagagaatactttcactcatcgcttactaagtaatccagaatatcaaactgaatataattcgaaatcagtcatttttaaggatggtttaaaaagttttgatatccggcagtttgagattcctacttattacctagataagaacttacttcttgattttgataaacagtctttacgtttaaccgatcctgcaactgaagaagttatcttaactgttccactaaaccaagttaaccaagttacaggtcctcatggtgtatggaaagttgctatctttacaaaagatcaatttgacgcgacctataatattaaaagtctatctttacctattgctgtaaatgcgattagtgcaaattatgctgtggccgaacgtggtaaacttactggggttttaggcttaacttatcaaggacaagataaagagcatattaccaaagtcttaaatgctattttagcgacttatagtgcgcaaaatattga

>LUH5554_[KC526900]

ttttcactcattgcacagtggaagcttattgcactctgcgttattttaagtcttgtatgtgctctactctacttacgtgtcaccccaaatacctactctgttgatgcgctagttcaagtagaagatagtaagggggcttctgctgcactattaggtgatttatctaatatgattgagcaaaaatctcctgctcaagctgaaatcgaaattctaaaatctcgattagttttaggctcagtaattaaggaattgcacctagatattcaaatttctagtactgaaaatacctttactcatcgtctattaagcgacgttgactataaaactgaatataataaaaaatttgttacttttaaggatggaccaaaaagttttgaaattcgtacattagaagttccagcctactatttagataaaactttacatcttaactttgataaacagtctttacgtttaactgacccagcaacagaacaaactcttttaacggtaccccttaatcagctaaatcaagtcacaggaccccatgggttttggaaagttggtatttttacaaaagatcattttgatacaacttataatatcaggagcttatctttacctgctgcagtcaatgtaataagttcaaattactccgttgcagaacaaggaaagcttaccggagtgttaggcttaaactatcaaggtcaagacaaagagcacattactaaagttttaaatgcaattttggcaacttatagtgcacaaaatattga

>KL4-AB0057[CP001182]

ttttcactgattgctcagtggaaactcattgctctatgcgttattttaagcgttgtatgtgccctactctatttacgtgtaacgccagatacctattcggtagatgctttagttcaggtagaagatagtaaaggagcttctgctgctcttctaggcgatttatcgaatatgattgagcagaaatcacctgctcaagctgaaattgaaattttaaaatctcgtttggttttgggctcggtcattaaagatttacatctgaatatacaggtctctagcactgaaaatacacttacccatcgcttattaagtgatactgactataaaactgaatatacaaaaaattcagtcatatttaaggatagtttaaagagttttgaaattcgtcagtttgaaattccagcctattatctcgatagaactttacaccttaactttgataaacagtctttacgtttaactgatccaaccactgaagaagtcatattaactttacctcttaaccaattaaaccaagtgacaggtccacaaggaacttggaaagttggtattttcactaaagatcagcttgaggccacttataatattaagagcttatctattcctgcagctgtaaatgctattagtgcaaattactctgttgctgaacgcggcaaacttactggagttttgggcttaaattatcaaggtcaagacaaagagcacattactaaagttttaaatgcaattttagtaacttatagcgcgcaaaatattga

>KL4-D81_[ NZ_FBXC00000000.1]

ttttcactgattgctcagtggaaactcattgctctatgcgttattttaagcgttgtatgtgccctactctatttacgtgtaacgccagatacctattcggtagatgctttagttcaggtagaagatagtaaaggagcttctgctgctcttctaggcgatttatcgaatatgattgagcagaaatcacctgctcaagctgaaattgaaattttaaaatctcgtttggttttgggctcggtcattaaagatttacatctgaatatacaggtctctagcactgaaaatacacttacccatcgcttattaagtgatactgactataaaactgaatatacaaaaaattcagtcatatttaaggatagtttaaagagttttgaaattcgtcagtttgaaattccagcctattatctcgatagaactttacaccttaactttgataaacagtctttacgtttaactgatccaaccactgaagaagtcatattaactttacctcttaaccaattaaaccaagtgacaggtccacaaggaacttggaaagttggtattttcactaaagatcagcttgaggccacttataatattaagagcttatctattcctgcagctgtaaatgctattagtgcaaattactctgttgctgaacgcggcaaacttactggagttttgggcttaaattatcaaggtcaagacaaagagcacattactaaagttttaaatgcaattttagtaacttatagcgcgcaaaatattga

>KL6-AB_908-14-7[AMHX00000000]

ttttcactcattgctcagtggaaactgattgcaatttgcatcattttaagccttgtttgtgcattgctttatttacgtgtaacaccagacacctactcagttgatgctttggttcaggttgaagacagcaagggtgcttctgcagcacttttaggcgatttgtcacaaatgattgagcaaaaatcaccagcccaagcagaaatagaaattttaaagtctcgtctggttttgggttcagtcattaaagatttacatctaaatattcaaatttccagtactgaaaatacatttactcatcgcttattaagtgatacggaatataaaactgagtacaccaaaaaatcggttttatttaaagatggcctaaaaagttttgaagtacgtgagcttgaagtccctgcatattacttagataaaaacttacttcttaattttgataagcagtctttacgtttaacagatccagatactgaagaagttatcttaactgttccattaaaccaagcgaaccatgttacaggccctcatggtttatggaaagttgccatcttcacaaaagaccaatttgacgcgacttataatattactaacttgtctttaccagctgctgtagatgcaataagttcaaattattcagttggtgagcgtggtaagttgacaggtgttttaggtcttaactatcaaggtcaagataaagagcatattaccaaagtacttaatgcgattttagtgacttatagtgctcaaaatattga

>KL6-AB_2008-15-45[AMHL00000000]

ttttcactcattgctcagtggaaactgattgcaatttgcatcattttaagccttgtttgtgcattgctttatttacgtgtaacaccagacacctactcagttgatgctttggttcaggttgaagacagcaagggtgcttctgcagcacttttaggcgatttgtcacaaatgattgagcaaaaatcaccagcccaagcagaaatagaaattttaaagtctcgtctggttttgggttcagtcattaaagatttacatctaaatattcacatttccagtactgaaaatacatttactcatcgcttattaagtgatacggaatataaaactgagtacaccaaaaaatcggttttatttaaagatggcctaaaaagttttgaagtacgtgagcttgaagtccctgcatattacttagataaaaacttacttcttaattttgataagcagtctttacgtttaacagatccagatactgaagaagttatcttaactgttccattaaaccaagcgaaccatgttacaggccctcatggtttatggaaagttgccatcttcacaaaagaccaatttgacgcgacttataatattactaacttgtctttaccagctgctgtagatgcaataagttcaaattattcagttggtgagcgtggtaagttgacaggtgttttaggtcttaactatcaaggtcaagataaagagcatattaccaaagtacttaatgcgattttagtgacttatagtgctcaaaatattga

>KL6a-1656_2[CP001921]

ttttcactcattgctcagtggaaactgattgcaatttgcatcattttaagccttgtttgtgcattgctttatttacgtgtaacaccagacacctactcagttgatgctttggttcaggttgaagacagcaagggtgcttctgcagcacttttaggcgatttgtcacaaatgattgagcaaaaatcaccagcccaagcagaaatagaaattttaaagtctcgtctggttttgggttcagtcattaaagatttacatctaaatattcaaatttccagtactgaaaatacatttactcatcgcttattaagtgatacggaatataaaactgagtacaccaaaaaatcggttttatttaaagatggcctaaaaagttttgaagtacgtgagcttgaagtccctgcatattacttagataaaaacttacttcttaattttgataagcagtctttacgtttaacagatccagatactgaagaagttatcttaactgttccattaaaccaagcgaaccatgttacaggccctcatggtttatggaaagttgccatcttcacaaaagaccaatttgacgcgacttataatattactaacttgtctttaccagctgctgtagatgcaataagttcaaattattcagttggtgagcgtggtaagttgacaggtgttttaggtcttaactatcaaggtcaagataaagagcatattaccaaagtacttaatgcgattttagtgacttatagtgctcaaaatattga

>KL6-AB_2008_15_70[AMHO00000000]

ttttcactcattgctcagtggaaactgattgcaatttgcatcattttaagccttgtttgtgcattgctttatttacgtgtaacaccagacacctactcagttgatgctttggttcaggttgaagacagcaagggtgcttctgcagcacttttaggcgatttgtcacaaatgattgagcaaaaatcaccagcccaagcagaaatagaaattttaaagtctcgtctggttttgggttcagtcattaaagatttacatctaaatattcacatttccagtactgaaaatacatttactcatcgcttattaagtgatacggaatataaaactgagtacaccaaaaaatcggttttatttaaagatggcctaaaaagttttgaagtacgtgagcttgaagtccctgcatattacttagataaaaacttacttcttaattttgataagcagtctttacgtttaacagatccagatactgaagaagttatcttaactgttccattaaaccaagcgaaccatgttacaggccctcatggtttatggaaagttgccatcttcacaaaagaccaatttgacgcgacttataatattactaacttgtctttaccagctgctgtagatgcaataagttcaaattattcagttggtgagcgtggtaagttgacaggtgttttaggtcttaactatcaaggtcaagataaagagcatattaccaaagtacttaatgcgattttagtgacttatagtgctcaaaatattga

>KL6-ABNIH3[AFTB00000000]

ttttcactcattgctcagtggaaactgattgcaatttgcatcattttaagccttgtttgtgcattgctttatttacgtgtaacaccagacacctactcagttgatgctttggttcaggttgaagacagcaagggtgcttctgcagcacttttaggcgatttgtcacaaatgattgagcaaaaatcaccagcccaagcagaaatagaaattttaaagtctcgtctggttttgggttcagtcattaaagatttacatctaaatattcacatttccagtactgaaaatacatttactcatcgcttattaagtgatacggaatataaaactgagtacaccaaaaaatcggttttatttaaagatggcctaaaaagttttgaagtacgtgagcttgaagtccctgcatattacttagataaaaacttacttcttaattttgataagcagtctttacgtttaacagatccagatactgaagaagttatcttaactgttccattaaaccaagcgaaccatgttacaggccctcatggtttatggaaagttgccatcttcacaaaagaccaatttgacgcgacttataatattactaacttgtctttaccagctgctgtagatgcaataagttcaaattattcagttggtgagcgtggtaagttgacaggtgttttaggtcttaactatcaaggtcaagataaagagcatattaccaaagtacttaatgcgattttagtgacttatagtgctcaaaatattga

>KL6-RBH4_[ KF130871]

ttttcactcattgctcagtggaaactgattgcaatttgcatcattttaagccttgtttgtgcattgctttatttacgtgtaacaccagacacctactcagttgatgctttggttcaggttgaagacagcaagggtgcttctgcagcacttttaggcgatttgtcacaaatgattgagcaaaaatcaccagcccaagcagaaatagaaattttaaagtctcgtctggttttgggttcagtcattaaagatttacatctaaatattcaaatttccagtactgaaaatacatttactcatcgcttattaagtgatacggaatataaaactgagtacaccaaaaaatcggttttatttaaagatggcctaaaaagttttgaagtacgtgagcttgaagtccctgcatattacttagataaaaacttacttcttaattttgataagcagtctttacgtttaacagatccagatactgaagaagttatcttaactgttccattaaaccaagcgaaccatgttacaggccctcatggtttatggaaagttgccatcttcacaaaagaccaatttgacgcgacttataatattactaacttgtctttaccagctgctgtagatgcaataagttcaaattattcagttggtgagcgtggtaagttgacaggtgttttaggtcttaactatcaaggtcaagataaagagcatattaccaaagtacttaatgcgattttagtgacttatagtgctcaaaatattga

>KL19-AB_1594-8[AMHD00000000]

ttttcactaattgctcagtggaaactagtcgcactctgcattattttaagccttgtatgtgcactactttatttacgtgtaacgccagatacctactcggttgatgctttggttcaagtcgaagacagcaaaggtgcttctgctgcacttttaggcgatttatcacaaatgattgagcaaaaatctcctgctcaagctgaaattgaaattctaaagtctcggttagttttaggttcggttattaaagaattacatttagatattcagatttctagcactgaaaataccttaactcaccgcttattaagtgatactgaatataaaactgaatacactaaaaattcagttttgtttaaagatggcactaaaaattttgatatacgaaaacttgaagttcctccatattatttagataaacagcttgtattaagctttgataaaaaatcttttaaattagtagatcctgagacagatgaggttgtactcacagctccactcaatcaaagtagtcaaattacaggagcctatggtgtatggaaggtggctatctttactaaagatttattagattcaaaatataatattaaaaaattgtctattcccgcagctgtagataatataagttcaaattattcagtagctgaacgcggtaaattaacaggtattcttggtttaaattatcaaggacatgacaaagaacatattactcaagtcttaaatgcgatcttagtgacatatggcgcccaaaatgttga

>KL19-AB_1536_8[AMHA00000000]

ttttcactaattgctcagtggaaactagtcgcactctgcattattttaagccttgtatgtgcactactttatttacgtgtaacgccagatacctactcggttgatgctttggttcaagtcgaagacagcaaaggtgcttctgctgcacttttaggcgatttatcacaaatgattgagcaaaaatctcctgctcaagctgaaattgaaattctaaagtctcggttagttttaggttcggttattaaagaattacatttagatattcaaatttctagcactgaaaataccttaactcaccgcttattaagtgatactgaatataaaactgaatacactaaaaattcagttttgtttaaagatggcactaaaaattttgatatacgaaaacttgaagttcctccatattatttagataaacagcttgtattaagctttgataaaaaatcttttaaattagtagatcctgagacagatgaggttgtactcacagctccactcaatcaaagtagtcaaattacaggagcctatggtgtatggaaggtggctatctttactaaagatttattagattcaaaatataatattaaaaaactgtctattcccgcagctgtagataatataagttcaaattattcagtagctgaacgcggtaaattaacaggtattcttggtttaaattatcaaggacatgacaaagaacatattactcaagtcttaaatgcgatcttagtgacatatggcgcccaaaatgttga

>KL19-AB_2009_04_01_7[AMHS00000000]

ttttcactaattgctcagtggaaactagtcgcactctgcattattttaagccttgtatgtgcactactttatttacgtgtaacgccagatacctactcggttgatgctttggttcaagtcgaagacagcaaaggtgcttctgctgcacttttaggcgatttatcacaaatgattgagcaaaaatctcctgctcaagctgaaattgaaattctaaagtctcggttagttttaggttcggttattaaagaattacatttagatattcagatttctagcactgaaaataccttaactcaccgcttattaagtgatactgaatataaaactgaatacactaaaaattcagttttgtttaaagatggcactaaaaattttgatatacgaaaacttgaagttcctccatattatttagataaacagcttgtattaagctttgataaaaaatcttttaaattagtagatcctgagacagatgaggttgtactcacagctccactcaatcaaagtagtcaaattacaggagcctatggtgtatggaaggtggctatctttactaaagatttattagattcaaaatataatattaaaaaattgtctattcccgcagctgtagataatataagttcaaattattcagtagctgaacgcggtaaattaacaggtattcttggtttaaattatcaaggacatgacaaagaacatattactcaagtcttaaatgcgatcttagtgacatatggcgcccaaaatgttga

>KL25-AB5075[AHAH00000000]

ttttcactgattgctcagtggaaactcattgctctatgcgttattttaagcgttgtatgtgccctactctatttacgtgtaacgccagatacctattcggtagatgctttagttcaggttgaggacagtaaaggggcttctgctgcacttttaggtgatttgtcagatatgattgagcaaaaatctcctgctcaagctgaaatcgagattttaaaatctcgtttggttttgggctcagtcattaaagatttacatcttaacatacaagtttctagcactgaaaatacacttactcatcgcttattaagtgatactgattataaaactgaatatacaaaaaactctgtgatatttaaggacggtttaaagagctttgaaattcgccagtttgaaattccagcttactatctcgataaaaccttacaacttaactttaataaacagtctttacgtttaactgacccagcgacagaacaaactcttttaacggtaccccttaatcagctaaatcaagtcacaggtccacaaggaatttggaaagttagtatttttactaaagataaacttgatacaacctataatattactagcttatctttaccagcagcagtagatactattggttcaaactattctgtagctgagagaggcaagcttacaggtgtgttaggtcttaactatcaaggtcaagacaaagagcatatcaccaaagttttaaatgctattttagcaacttatagtgcgcaaaatattga

>Ab689_KL13[MF522810.1]

ttttcactgattgcacagtggaagcttatcgctctatgcgttattttaagcctagtatgtgctctattgtacttacgggtaacccctgatacttattcagtggatgcactggttcaagttgaagacagtaaaggggcttctgctgcgcttttaggtgacttatcacaaatgattgagcaaaaatcaccagctcaagcagaaattgagattttaaaatctcgtttagttttaggttcagtcattaaagacttacatctcaacatacaagtttctagtaccgaaaatacatttactcatcgtttattgagtaatccagaatataaaactgaatatactaagaaatctgttatttttaaggatggattaaaaagttttgatatccatcagtttgatataccagcttattacttagataaaaatttatttcttgattttgataaacaatctttccgtttaacagacccaaagactgaagaagttatcttaactcttccacttaatcagttaaaccaagttactggcccacatgggacgtggaaagttgctatctttacaaaagaccaatttgatgccacttataatattagaaatctatctttacctgctgctgtaaattcaattagttcaaactactcagttgctgaacgtggtaaattaacgggtattctaggtttaaattatcagggtcaagatcaagaacatattactaaggttttaaacgcaattttagctacttacagtgcacaaaatattga

>OIFC180[AMDQ00000000]

ttttcactgattgcacagtggaagcttatcgctctatgcgttattttaagcctagtatgtgctctattgtacttacgggtaacccctgatacttattcagtggatgcactggttcaagttgaagacagtaaaggggcttctgctgcgcttttaggtgacttatcacaaatgattgagcaaaaatcaccagctcaagcagaaattgagattttaaaatctcgtttagttttaggttcagtcattaaagacttacatctcaacatacaagtttctagtaccgaaaatacatttactcatcgtttattgagtaatccagaatataaaactgaatatactaagaaatctgttatttttaaggatggattaaaaagttttgatatccatcagtttgatataccagcttattacttagataaaaatttatttcttgattttgataaacaatctttccgtttaacagacccaaagactgaagaagttatcttaactcttccacttaatcagttaaaccaagttactggcccacatgggacgtggaaagttgctatctttacaaaagaccaatttgatgccacttataatattagaaatctatctttacctgctgctgtaaattcaattagttcaaactactcagttgctgaacgtggtaaattaacgggtattctaggtttaaattatcagggtcaagatcaagaacatattactaaggttttaaacgcaattttagctacttacagtgcacaaaatattga

>KL13-AB_2008_15_34_7[AMHK00000000]

ttttcactgattgcacagtggaagcttatcgctctatgcgttattttaagcctagtatgtgctctattgtacttacgggtaacccctgatacttattcagtggatgcactggttcaagttgaagacagtaaaggggcttctgctgcgcttttaggtgacttatcacaaatgattgagcaaaaatcaccagctcaagcagaaattgagattttaaaatctcgtttagttttaggttcagtcattaaagacttacatctcaacatacaagtttctagtaccgaaaatacatttactcatcgtttattgagtaatccagaatataaaactgaatatactaagaaatctgttatttttaaggatggattaaaaagttttgatatccatcagtttgatataccagcttattacttagataaaaatttatttcttgattttgataaacaatctttccgtttaacagacccaaagactgaagaagttatcttaactcttccacttaatcagttaaaccaagttactggcccacatgggacgtggaaagttgctatctttacaaaagaccaatttgatgccacttataatattagaaatctatctttacctgctgctgtaaattcaattagttcaaactactcagttgctgaacgtggtaaattaacgggtattctaggtttaaattatcagggtcaagatcaagaacatattactaaggttttaaacgcaattttagctacttacagtgcacaaaatattga

>KL13-6014059[ACYS02000025]

ttttcactgattgcacagtggaagcttatcgctctatgcgttattttaagcctagtatgtgctctattgtacttacgggtaacccctgatacttattcagtggatgcactggttcaagttgaagacagtaaaggggcttctgctgcgcttttaggtgacttatcacaaatgattgagcaaaaatcaccagctcaagcagaaattgagattttaaaatctcgtttagttttaggttcagtcattaaagacttacatctcaacatacaagtttctagtaccgaaaatacatttactcatcgtttattgagtaatccagaatataaaactgaatatactaagaaatctgttatttttaaggatggattaaaaagttttgatatccatcagtttgatataccagcttattacttagataaaaatttatttcttgattttgataaacaatctttccgtttaacagacccaaagactgaagaagttatcttaactcttccacttaatcagttaaaccaagttactggcccacatgggacgtggaaagttgctatctttacaaaagaccaatttgatgccacttataatattagaaatctatctttacctgctgctgtaaattcaattagttcaaactactcagttgctgaacgtggtaaattaacgggtattctaggtttaaattatcagggtcaagatcaagaacatattactaaggttttaaacgcaattttagctacttacagtgcacaaaatattga

>KL13-Naval_17[AFDO00000000]

ttttcactgattgcacagtggaagcttatcgctctatgcgttattttaagcctagtatgtgctctattgtacttacgggtaacccctgatacttattcagtggatgcactggttcaagttgaagacagtaaaggggcttctgctgcgcttttaggtgacttatcacaaatgattgagcaaaaatcaccagctcaagcagaaattgagattttaaaatctcgtttagttttaggttcagtcattaaagacttacatctcaacatacaagtttctagtaccgaaaatacatttactcatcgtttattgagtaatccagaatataaaactgaatatactaagaaatctgttatttttaaggatggattaaaaagttttgatatccatcagtttgatataccagcttattacttagataaaaatttatttcttgattttgataaacaatctttccgtttaacagacccaaagactgaagaagttatcttaactcttccacttaatcagttaaaccaagttactggcccacatgggacgtggaaagttgctatctttacaaaagaccaatttgatgccacttataatattagaaatctatctttacctgctgctgtaaattcaattagttcaaactactcagttgctgaacgtggtaaattaacgggtattctaggtttaaattatcagggtcaagatcaagaacatattactaaggttttaaacgcaattttagctacttacagtgcacaaaatattga

>KL13-UMB001[AEPK00000000]

ttttcactgattgcacagtggaagcttatcgctctatgcgttattttaagcctagtatgtgctctattgtacttacgggtaacccctgatacttattcagtggatgcactggttcaagttgaagacagtaaaggggcttctgctgcgcttttaggtgacttatcacaaatgattgagcaaaaatcaccagctcaagcagaaattgagattttaaaatctcgtttagttttaggttcagtcattaaagacttacatctcaacatacaagtttctagtaccgaaaatacatttactcatcgtttattgagtaatccagaatataaaactgaatatactaagaaatctgttatttttaaggatggattaaaaagttttgatatccatcagtttgatataccagcttattacttagataaaaatttatttcttgattttgataaacaatctttccgtttaacagacccaaagactgaagaagttatcttaactcttccacttaatcagttaaaccaagttactggcccacatgggacgtggaaagttgctatctttacaaaagaccaatttgatgccacttataatattagaaatctatctttacctgctgctgtaaattcaattagttcaaactactcagttgctgaacgtggtaaattaacgggtattctaggtttaaattatcagggtcaagatcaagaacatattactaaggttttaaacgcaattttagctacttacagtgcacaaaatattga

>KL5-SDF[CU468230]

ttttcattgattgctcagtggaaactcattgctctatgcgttattttaagcgttgtatgtgccctactctatttacgtgtaacgccagatacctattcggtagatgctttggttcaggttgaggatagtaaaggggcttctgctgcgcttttaggtgatttatcacaaatgatcgagcaaaaatcaccagctcaggcagaaatagaaattttaaaatcacgtctggttttaggttctgtgattaaggatttacatcttaatatacaggtctctagcactgaaaatacgcttacccatcgcttattgagtgatactgactataaaactgaatatactacgaaatcagtcatttttaaggatggcttaaaaagttttgatatccgtcagtttgagattcctacttattacctagataagaacttacttcttgattttgataaacaatctttacgtttaacaaacccagatactgaagaagtcatcttaactgttccattaaaccaagttaaccaagttacaggtcctcatggtgtatggaaagttgctatctttacaaaagatcaatttgacgcgacctataatattaaaagtttatctttacctattgctgtaaatgctattagtgcgaattatgctgtggccgaacgtggtaaacttactggggttttaggtctaagttaccaagggcaagacaaagaacatatcactaaagttttaaacgctattttagcgacttatagtgcacaaaatattga

>KL26-BZICU-2[ALOH00000000]

ttttcattgattgcacagtggaaaatcatagcatgctgcgtgatcttaagtttaatttgtgccttgttatatttacgcataacaacagatacttattctgtagatgctatggtacaagttgaagacagtaaaggcgctgcctctgctgctttattgggagacctttctaaagttactggaggtatatctcaaaaatcaccagctgatccagaaatagagattttaaaatctcgtatggttttagggcaagtgattcataaccttaatcttgatattaaaattaaagataatcaatcaggattgattgataagttaatttcacaagataagagtaaaattgaatatcgtcatgaatcggtagtttacaataacttaaacgctagtttgattattcaagaatttaaggtgcctgaatattattttgataaacctttgaaattagagtttaaagatacaaatcaatttactctaatgtataaagatcaagttgtatttaatggtcaattaaacaagaaaaatatattaaattcaaataaaggtttatggcaagttcaaatcaatgcacaaggcaatttaaaagatcaaagttatactttaactaaattagctcttttaacagctgtaaatcaatttaattcaatatattcagttaatgaaaaaggtaaaatgaccggagttatcggtttaagctatttaggccaagatcctgaacatattactcaagtgctcaataatgttctaaatgtttatcatgaacaaaatattga-

>BAL_097_KL8[KX712116.2]

ttttcactcattgcacagtggaagcttattgctctatgcattattttaagtcttgtatgtgctctactttatttacgtgtaacaccagatacctattcggttgatgctttggttcaggtcgaagacagtaaaggggcttctgctgcgcttttgggtgatctgtctaatatgattgagcaaaagtctccagctcaagcagaaattgagattttaaagtcgcgattagttttaggttctgtgattaaagatttacaccttaatatacaaatttccagtactgaaaatacattaactcaccgtttattaagcgatgttgagtataaaacagaatataataaacaagcagttatttttaaagatgggtttaaaagttttgaaattagacagtttgagattcctgcttattatttagataaaaatttatatcttagttttgacaaacaatctttccgtttaactgatcctaatactgaacaaactatattgacgctaccacttaatcagttaaaccaagttgctggtccacatgggacttggaaagttgctatctttacaagagatcaatttaacaccacttataatattaccaatttatccttgcctgcagctgtaaatttagtgagttctaattattctgttgcagaacgtggcaagcttacaggtgttcttggattaaattatcaaggtgaggataaacaacatattactaaagttttaaatgctattttagctacctatagtgcccaaaatattga

>KL36-Naval-72[AMFI00000000]

ttttcattgattgcacagtggaaaatcatagcatgctgcgtggtcttaagtttaatttgtgccttgttatatttgcgtataacaccagacatctacgctgtagatgcaatggtacaagttgaagatagtaaaggtgctgcctctgctgctttactgggagatctttccaaagttagtggtggcttaacacaaaaatcaccagccgatccagaaatagagattcttcgttctagaatggttttgggacaagtgattcataatctcaatcttgatattaaaattaaagataatcaattaggtttgattggtaaacttgtttcacaagataaaagtaaacttgagtatcatcatgatgctgtaacttatattaatcaaaacaatagattgatagttaaacaactgaacatccctgaatattatttagataaacctctaaaattagagtttaaagatgtaaatcaatttactctaacctataaagatcaagttgtattcagtggactgttaaacaagaaaaatgtactaaatggcgaaaaaggtttatggcaagtacaacttgatgcacaaggtgatttaaaaaaacagagtttcactttaactaaagcagctcttccaacggctgttaagaattttaataatatttatggtgttgcagaaaaaggaaagatgacaggggttattggtctaagttatttaggtcaagatcctgagcacattacgcaagtacttaataatgttttaaatgtttatcatgaacaaaacattga-

>KL23-OIFC143[AFDL00000000]

ttttcactcattgctcagtggaaactgattgcaatttgcatcattttaagccttgtttgtgcattgctttatttacgtgtaacaccagacacctactcagttgatgctttggttcaggttgaagacagtaaaggagcttctgcagcacttttaggcgatttgtcacaaatgattgagcaaaaatcacccgctcaagcagaaatagaaattttaaagtctcgtctggttttgggttcagtcattaaagatttacatctaaatattcaaatttccagtactgaaaatacatttactcatcgcttattaagtgatacggaatataaaactgagtacaccaaaaaatcggttttatttaaagatggcctaaaaagttttgaagtacgtgagcttgaagtccctgcatattacttagataaaaacttacttcttaattttgataagcagtctttacgtttaacagatccagatactgaagaagttatcttaactgttccattaaaccaagcgaaccatgttacaggccctcatggtttatggaaagttgccatcttcacaaaagaccaatttgacgcgacttataatattactaacttgtctttaccagctgctgtagatgcaataagttcaaattattcagttggtgagcgtggtaagttgacaggtgttttaggtcttaactatcaagggcaagataaagaacatatcactaaagttttaaatgctattttagcgacttatagtgcccaaaatattga

>KL23-WC-A-92[NZ_AMFU00000000.1]

ttttcactcattgctcagtggaaactgattgcaatttgcatcattttaagccttgtttgtgcattgctttatttacgtgtaacaccagacacctactcagttgatgctttggttcaggttgaagacagtaaaggagcttctgcagcacttttaggcgatttgtcacaaatgattgagcaaaaatcacccgctcaagcagaaatagaaattttaaagtctcgtctggttttgggttcagtcattaaagatttacatctaaatattcaaatttccagtactgaaaatacatttactcatcgcttattaagtgatacggaatataaaactgagtacaccaaaaaatcggttttatttaaagatggcctaaaaagttttgaagtacgtgagcttgaagtccctgcatattacttagataaaaacttacttcttaattttgataagcagtctttacgtttaacagatccagatactgaagaagttatcttaactgttccattaaaccaagcgaaccatgttacaggccctcatggtttatggaaagttgccatcttcacaaaagaccaatttgacgcgacttataatattactaacttgtctttaccagctgctgtagatgcaataagttcaaattattcagttggtgagcgtggtaagttgacaggtgttttaggtcttaactatcaaggtcaagacaaagagcatattaccaaagtacttaatgcgattttagtgacttatagtgctcaaaatattga

>KL31-OIFC0162[AMFH00000000]

ttttcactcattgctcagtggaaactgattgcaatttgcatcattttaagccttgtttgtgcattgctttatttacgtgtaacaccagacacctactcagttgatgctttggttcaggttgaagacagcaagggtgcttctgcagcacttttaggcgatttgtcacaaatgattgagcaaaaatcacccgctcaagcagaaatagaaattttaaagtctcgtctggttttgggttcagtcattaaagatttacatctaaatattcaaatttccagtactgaaaatacatttactcatcgcttattaagtgatacggaatataaaactgagtacaccaaaaaatcggttttatttaaagatggcctaaaaagttttgaagtacgtgagcttgaagtccctgcatattacttagataaaaacttacttcttaattttgataagcagtctttacgtttaacagatccagatactgaagaagttatcttaactgttccattaaaccaagcgaaccatgttacaggccctcatggtttatggaaagttgccatcttcacaaaagaccaatttgacgcgacttataatattactaacttgtctttaccagctgctgtagatgcaataagttcaaattattcagttggtgagcgtggtaagttgacaggtgttttaggtcttaactatcaaggtcaagataaagagcatattaccaaagtacttaatgcgattttagtgacttatagtgctcaaaatattga

>KL10-TYTH_1[CP003856]

ttttctttgattgcacagtggaaaatcatagcatgctgtgtgattttaagtttaatttgtgccttattatatttacggataacaccagatatttatgctgtagatgcaatggtacaagtggaagatagtaaaggcgctgcctctgctgctttgcttggagacctttctaaagtcagtggaggtctatctcaaaaatcaccagctgatccagaaatagagattttaaaatctcgtatggttttagggcaagtcatccagaatcttaatcttgatattaacatcaaagataatcaatcaggattgattgataagttaatttcacaagataagagtaaaattgaatatcatcatgaatctgtactttacagtaatcaaaataataatttgattatacgagaatttaaagtacctgaatattattttgataaacctttaaaattagagtttaaaggtacaaatcaattcactttaatgtacaaagatcaagttgtatttaatggcctgctaaacaagaaaaatatactaaacacagataggggcctttggcagatacaaattaatgctcaaggtaatttgaaagaacaaagctacactttaactaagctcgctctaccaacagctgttaaaaattttaataatatttatagtgttgccgagaaaggtaaattgacgggcgttattggtttaaattatctaggtcaagatcctgagcacattacacaagtgctcaataacgttctaaatgtttatcatcaacaaaatattga-

>BAL_030_KL10[KY434633.1]

ttttcattgattgcacagtggaaaatcatagcatgctgcgtggtcttaagtttaatttgtgccttgctatacttacggataacgccagatacttattctgtagatgcaatggtacaagttgaggacagtaaaggcgcagcctctgctgctttgcttggtgacctttctaaagtcagtggaggtctatctcaaaaatcaccagctgatccagaaatagagattcttcgttctagaatggttttgggacaagtcatccagaaccttaatcttgatattaacgtcaaagataaccaatcaggattgatcgataagttaatttcacaagataagagtagacttgagtatcgtcatgaatctgtactgtacagtaatcaaaataataatttgattatacgagaacttaaagtacctgaatattatttagataaacccttaaagttagagttcaaaggtgcaaatcaatttactttaacgtacaaagatcaagttgtatttgatggtcaattaaacaagaaaaatatactaaatacagatagagggctttggcaggtacaacttaatgctcaaggtaatctgaaagaacaaagctacactttaactaagctcgctctaccaacagccgttaaaaattttaatgatatctatagtgttgctgaaaaaggtaaagtgaccggtgttattggtttaaattatctaggccaagatcctgagcacattacacaagtacttaataatgttttaaatgtttatcatgaacaaaatattga-

>XH857[CP014540.1]

ttttcattgattgcacagtggaaaatcatagcatgctgcgtggtcttaagtttaatttgtgccttgctatacttacggataacgccagatacttattctgtagatgcaatggtacaagttgaggacagtaaaggcgctgcctctgctgctttgcttggtgacctttctaaagtcagtggaggtctatctcaaaaatcaccagctgatccagaaatagagattcttcgttctagaatggttttaggacaagtcatccagaaccttaatcttgatattaacgtcaaagataaccaatcaggattgatcgacaagttaatttcacaagataagagtagacttgagtatcgtcatgaatctgtactgtacagtaatcaaaataataatttgattatacgagaacttaaagtacctgaatattatttagataaacccttaaagttagagttcaaaggtgcaaatcaattcactttaacatacaaagatcaagttgtatttaatggtcaattaaacaaaaaaaatatactaaatacaaatagaggtctttggcaggtacaacttaatgctcaaggtaacctgaaagaacaaagctatactttaactaaactcgctctaccaacagccgttaaaaattttaatgatatctatagtgttgctgaaaaaggtaaagtgaccggtgttattggtttaaattatctaggccaagatcctgagcacattacacaagtacttaataatgttttaaatgtttatcatgaacaaaatattga-

>NCGM_237[AP013357.1]

ttttctttgattgcacagtggaaaatcatagcatgctgtgtgattttaagtttaatttgtgccttattatatttacggataacaccagatatttatgctgtagatgcaatggtacaagtggaagatagtaaaggcgctgcctctgctgctttgcttggagacctttctaaagtcagtggaggtctatctcaaaaatcaccagctgatccagaaatagagattttaaaatctcgtatggttttagggcaagtcatccagaatcttaatcttgatattaacatcaaagataatcaatcaggattgattgataagttaatttcacaagataagagtaaaattgaatatcatcatgaatctgtactttacagtaatcaaaataataatttgattatacgagaatttaaagtacctgaatattattttgataaacctttaaaattagagtttaaaggtacaaatcaattcactttaatgtacaaagatcaagttgtatttaatggcctgctaaacaagaaaaatatactaaacacagataggggcctttggcagatacaaattaatgctcaaggtaatttgaaagaacaaagctacactttaactaagctcgctctaccaacagctgttaaaaattttaataatatttatagtgttgccgagaaaggtaaattgacgggcgttattggtttaaattatctaggtcaagatcctgagcacattacacaagtgctcaataacgttctaaatgtttatcatcaacaaaatattga-

>KL33-WC_141[AMSS00000000]

ttttcactcattgctcagtggaagctgatcgcgctctgtattattttgagccttgtgtgtgccctgctctatttacgtgtaacaccggatacctattcggttgatgctttggttcaggttgaagatagtaaaggggcctctgcagcacttttaggcgatttgtcacaaatgattgagcaaaaatcaccagctcaagcagaaattgaaattttaaaatcacgtctggttttaggttcagtcattaaagatttacatctgaatatacaagtttccagcacagaaaatacccttactcatcgtttattaagcgatactgaatataaaactgaatacacccaaaaatcggttttatttaaagacgacttaaaaagttttgaaattcgtgaatttgaagtaccagcatactatttagataaaaatttacttcttaattttgataaacaatctttacgtttaactgatccaaaaacagaagaggtcctattaaccgttcctctaaatcatgctaaccgtgttgctggtcctcacggagtttggaaagttgccgtctttactaaagatcagcttgatgcaacttataacattactaattcatctttacctgcaactgtagatgctattactgctaactactcagtaggagaacgcggtaagcttactggagtgctaggtttaaattatcagggtcaagataaagagcatatcaccaaagtacttaatgcaattttagcgacttatagtgcacaaaatattga

>APD1[CR543861]

ttttcactgattgtgcagtggaaacttatcttactttgcgtattacttagcatagttttagcgttactttatttacgtgttacatccgatacttactctgtcgatgctctcgtgcaagttgaaagtcctaaaggcggtgcttcagcagcactgttaggtcaagaactttctaacgttatggatacgtctggactaggccaacagcttgcgcaagcagaaattgagatattgagatcacgcttggttgtaggtacaaccatcgagaaattaaatctggatatcactgttcaacctaaaaatgactccgtgattcaacgattaatttcaagctcagattttagcactcaatattcttcacgtggcgtactggtagaaaatgattcagatcatttcgatattcaacaatttactgtacctgaaaaatatttgaacagctcactgctattaaatatatcagagaacaagcagattaccctgacagatcttgataaagaagaagtggtttttaaagggcagttaaatcaaaacaatatcttatctacccgtgatggtctctggaaagttactatttttggtaatcctttagaagaagaatatattgttaccaaacaagcacttccaacagcagtaaatagcctcctagataacttttctgccgccgaacgtggcaaacaaacaggtgtcattgggcttagctatcaaggtcatgataagactcaaattaccagtgtactgaatgttattttgcaaacctataagcagcaaaatattga

>DR1[CP002080]

ttttcactaattgcccaatggaaactaattgcgctctgcattattttgagcctcgtatgcgctttactgtatttacgcgtaacgccagatacctattcggtggatgctttggttcaggttgaagatagtaaaggggcttctgccgctcttttaggtgaactatcaaatgttatggagcaaaaatcaccggcccaagcggagattgagattttacaatcccgtttggttctaggctctgtaattaaaaacttacatcttgatatacaagtttccagcacagaagatactctttctcatcgcctattgagtgatactgattataaaactgaatacactaaagatgctgttttatttaaagatgggctaaagagttttgaaatccgtcaatttgaaattccagcttactatttagataggaatttaatccttaattttgataaacaatccgtgcgtttaagtgatgctagtactgagcagactattctaactgtaccacttaatcagttaaatcaagtcacaggaccacatggctcttggaaagttgctgtttttagtaaagatcaatttgatacttcttataatattaagcatttatctttacctgcagcagttggggcaattagctcaaattattctgtagcagagcaaggaaaactaacaggtgttttaggcctaacgtatcaaggtcaagataaagaacatattactaaagttttaaatgcgattttagcaacttatagtgctcaaaatattga

>PHEA-2[CP002177]

ttttcactaattgcccaatggaaacttattgcgctctgcattattttgagcctcgtatgcgctttactgtatttacgcgtaacgccagatacctattcggtggatgctttggttcaggttgaagatagtaaaggggcttctgccgctcttttaggtgaactatcaaatgttatggagcaaaaatcacctgcccaagcggagattgaaattttacaatcacgtctggttctaggctctgtaattaaaaacttacatcttgatatacaagtttccagcacagaagatactctttctcatcgcctattgagtgatactgattataaaactgaatacactaaagatggtgttttatttaaagatgggctaaaaagttttgaaatccgtcaatttgaaattccagcttactatttagatagaaatttaattcttaattttgataaacaatccgtgcgtttaagtgatgctagtactgagcagactattctaactgtaccacttaatcaattaaatcaagtcacaggtccacatggctcttggaaagttgctatttttagtaaagatcaatttaatacttcttataatattaagcatttatctttacctgcagcagttggttcaattagctcaaattattctgtagcagaacaaggaaaactaacaggtgttctaggactgacttatcaaggtcaagataaagaacatattactaaagttttaaatgcgattttggcaacttatagtgctcaaaatattga

>Ab908[MF522807.1]

ttttcactgattgctcagtggaagcttattgctctatgcgttattttaagcgttgtatgtgctctactctatttacgtgtaacgccagatacctattcggtagatgctttggttcaggttgaagacagtaagggagcttctgctgcgcttttaggtgatttatcacaaatgatcgagcaaaaatcaccagctcaggcagaaatagaaattttaaaatctcgtttggttttaggttctgtgattaaagatttacatcttaatatacaggtctctagcacagagaatactttcactcatcgcttactaagtaatccagaatatcaaactgaatataattcgaaatcagtcatttttaaggatggtttaaaaagttttgatatccggcagtttgagattcctacttattacctagataagaacttacttcttgattttgataaacagtctttacgtttaaccgatcctgcaactgaagaagttatcttaactgttccactaaaccaagttaaccaagttacaggtcctcatggtgtatggaaagttgctatctttacaaaagatcaatttgacgcgacctataatattaaaagtctatctttacctattgctgtaaatgcgattagtgcaaattatgctgtggccgaacgtggtaaacttactggggttttaggcttaacttatcaaggacaagataaagagcatattaccaaagtcttaaatgctattttagcaacttatagtgcgcaaaatattga

>OIFC035[AMTB01000027]

ttttcactgattgctcagtggaagcttattgctctatgcgttattttaagcgttgtatgtgctctactctatttacgtgtaacgccagatacctattcggtagatgctttggttcaggttgaagacagtaagggagcttctgctgcgcttttaggtgatttatcacaaatgatcgagcaaaaatcaccagctcaggcagaaatagaaattttaaaatctcgtttggttttaggttctgtgattaaagatttacatcttaatatacaggtctctagcacagagaatactttcactcatcgcttactaagtaatccagaatatcaaactgaatataattcgaaatcagtcatttttaaggatggtttaaaaagttttgatatccggcagtttgagattcctacttattacctagataagaacttacttcttgattttgataaacagtctttacgtttaaccgatcctgcaactgaagaagttatcttaactgttccactaaaccaagttaaccaagttacaggtcctcatggtgtatggaaagttgctatctttacaaaagatcaatttgacgcgacctataatattaaaagtctatctttacctattgctgtaaatgcgattagtgcaaattatgctgtggccgaacgtggtaaacttactggggttttaggcttaacttatcaaggacaagataaagagcatattaccaaagtcttaaatgctattttagcaacttatagtgcgcaaaatattga

>KL12-6013113[ACYR02000042]

ttttcactgattgcacagtggaagcttatcgctctatgcgttattttaagcctagtatgtgctctattgtacttacgggtaacccctgatacttattcagtggatgcactggttcaagttgaagacagtaaaggggcttctgctgcgcttttaggtgacttatcacaaatgattgagcaaaaatcaccagctcaagcagaaattgagattttaaaatctcgtttagttttaggttcagtcattaaagacttacatctcaacatacaagtttctagtaccgaaaatacatttactcatcgtttattgagtaatccagaatataaaactgaatatactaagaaatctgttatttttaaggatggattaaaaagttttgatatccatcagtttgatataccagcttattacttagataaaaatttatttcttgattttgataaacaatctttccgtttaacagacccaaagactgaagaagttatcttaactcttccacttaatcagttaaaccaagttactggcccacatgggacgtggaaagttgctatctttacaaaagaccaatttgatgccacttataatattagaaatctatctttacctgctgctgtaaattcaattagttcaaactactcagttgctgaacgtggtaaattaacgggtattctaggtttaaattatcagggtcaagatcaagaacatattactaaggttttaaacgcaattttagctacttacagtgcacaaaatattga

>KL12-6013150[ACYQ02000047]

ttttcactgattgcacagtggaagcttatcgctctatgcgttattttaagcctagtatgtgctctattgtacttacgggtaacccctgatacttattcagtggatgcactggttcaagttgaagacagtaaaggggcttctgctgcgcttttaggtgacttatcacaaatgattgagcaaaaatcaccagctcaagcagaaattgagattttaaaatctcgtttagttttaggttcagtcattaaagacttacatctcaacatacaagtttctagtaccgaaaatacatttactcatcgtttattgagtaatccagaatataaaactgaatatactaagaaatctgttatttttaaggatggattaaaaagttttgatatccatcagtttgatataccagcttattacttagataaaaatttatttcttgattttgataaacaatctttccgtttaacagacccaaagactgaagaagttatcttaactcttccacttaatcagttaaaccaagttactggcccacatgggacgtggaaagttgctatctttacaaaagaccaatttgatgccacttataatattagaaatctatctttacctgctgctgtaaattcaattagttcaaactactcagttgctgaacgtggtaaattaacgggtattctaggtttaaattatcagggtcaagatcaagaacatattactaaggttttaaacgcaattttagctacttacagtgcacaaaatattga

>KL12-D36[JN107991.2]

ttttcactgattgcacagtggaagcttatcgctctatgcgttattttaagcctagtatgtgctctattgtacttacgggtaacccctgatacttattcagtggatgcactggttcaagttgaagacagtaaaggggcttctgctgcgcttttaggtgacttatcacaaatgattgagcaaaaatcaccagctcaagcagaaattgagattttaaaatctcgtttagttttaggttcagtcattaaagacttacatctcaacatacaagtttctagtaccgaaaatacatttactcatcgtttattgagtaatccagaatataaaactgaatatactaagaaatctgttatttttaaggatggattaaaaagttttgatatccatcagtttgatataccagcttattacttagataaaaatttatttcttgattttgataaacaatctttccgtttaacagacccaaagactgaagaagttatcttaactcttccacttaatcagttaaaccaagttactggcccacatgggacgtggaaagttgctatctttacaaaagaccaatttgatgccacttataatattagaaatctatctttacctgctgctgtaaattcaattagttcaaactactcagttgctgaacgtggtaaattaacgggtattctaggtttaaattatcagggtcaagatcaagaacatattactaaggttttaaacgcaattttagctacttacagtgcacaaaatattga

>KL24-1043794[JEYX01000013]

ttctcattgattgctcaatggaaactgattgtattatgtattcttataagtctagtctgtgcattaatttacttacgtatcacaccaaatacttattctgttgacgcattggttcaagtagaagatagtaaaggtgcagcttcagcagcattattaggtgaactatctaaaacggtaggcattgaacaaaaatctccagcagatgctgaaatacaaattcttagttcacgtatggtacttagtcaagttatacataacttaaatctagatatcacaattaaaaatcatgatgatactttcttcaaccgtcttttaaaccaagataaacagaatattgattataagaaagatgctgtaacttttagtaataaagatagttacttctctatccaacagcttcagattccttcgtattatcttgataaaccccttttactgtcttttaaagatcagaggcatttcactttcagctataaagataaagttatttttaatggtcagctcaatagtaataatttagtaactgctcgtgaaggacaatggaaagtacgaattaattctacacacgctccatctgtagagcagcaatttacaataagtaagtttgctctaccaactgcattacaaaaattcagttctacttacggcgtagctgaaaaaggtaaacaaacgggagttattgctttaaatttccaaggcactgataaagagcatatcactgaagtattaaacaatgtcttagccgtataccatagccagaatattga-

>KL24-BAL255[CZWB01000054]

ttctcattgattgctcaatggaaactgattgtattatgtattcttataagtctagtctgtgcattaatttacttacgtatcacaccaaatacttattctgttgacgcattggttcaagtagaagatagtaaaggtgcagcttcagcagcattattaggtgaactatctaaaacggtaggcattgaacaaaaatctccagcagatgctgaaatacaaattcttagttcacgtatggtacttagtcaagttatacataacttaaatctagatatcacaattaaaaatcatgatgatactttcttcaaccgtcttttaaaccaagataaacagaatattgattataagaaagatgctgtaacttttagtaataaagatagttacttctctatccaacagcttcagattccttcgtattatcttgataaaccccttttactgtcttttaaagatcagaggcatttcactttcagctataaagataaagttatttttaatggtcagctcaatagtaataatttagtaactgctcgtgaaggacaatggaaagtacgaattaattctacacacgctccatctgtagagcagcaatttacaataagtaagtttgctctaccaactgcattacaaaaattcagttctacttacggcgtagctgaaaaaggtaaacaaacgggagttattgctttaaatttccaaggcactgataaagagcatatcactgaagtattaaacaatgtcttagccgtataccatagccagaatattga-

>KL24-232184[JEYI01000009]

ttctcattgattgctcaatggaaactgattgtattatgtattcttataagtctagtctgtgcattaatttacttacgtatcacaccaaatacttattctgttgacgcattggttcaagtagaagatagtaaaggtgcagcttcagcagcattattaggtgaactatctaaaacggtaggcattgaacaaaaatctccagcagatgctgaaatacaaattcttagttcacgtatggtacttagtcaagttatacataacttaaatctagatatcacaattaaaaatcatgatgatactttcttcaaccgtcttttaaaccaagataaacagaatattgattataagaaagatgctgtaacttttagtaataaagatagttacttctctatccaacagcttcagattccttcgtattatcttgataaaccccttttactgtcttttaaagatcagaggcatttcactttcagctataaagataaagttatttttaatggtcagctcaatagtaataatttagtaactgctcgtgaaggacaatggaaagtacgaattaattctacacacgctccatctgtagagcagcaatttacaataagtaagtttgctctaccaactgcattacaaaaattcagttctacttacggcgtagctgaaaaaggtaaacaaacgggagttattgctttaaatttccaaggcactgataaagagcatatcactgaagtattaaacaatgtcttagccgtataccatagccagaatattga-

>KL24-268680[JEYN01000012]

ttctcattgattgctcaatggaaactgattgtattatgtattcttataagtctagtctgtgcattaatttacttacgtatcacaccaaatacttattctgttgacgcattggttcaagtagaagatagtaaaggtgcagcttcagcagcattattaggtgaactatctaaaacggtaggcattgaacaaaaatctccagcagatgctgaaatacaaattcttagttcacgtatggtacttagtcaagttatacataacttaaatctagatatcacaattaaaaatcatgatgatactttcttcaaccgtcttttaaaccaagataaacagaatattgattataagaaagatgctgtaacttttagtaataaagatagttacttctctatccaacagcttcagattccttcgtattatcttgataaaccccttttactgtcttttaaagatcagaggcatttcactttcagctataaagataaagttatttttaatggtcagctcaatagtaataatttagtaactgctcgtgaaggacaatggaaagtacgaattaattctacacacgctccatctgtagagcagcaatttacaataagtaagtttgctctaccaactgcattacaaaaattcagttctacttacggcgtagctgaaaaaggtaaacaaacgggagttattgctttaaatttccaaggcactgataaagagcatatcactgaagtattaaacaatgtcttagccgtataccatagccagaatattga-

>KL24-Ab655378[NZ_JFCE00000000.2]

ttctcattgattgctcaatggaaactgattgtattatgtattcttataagtctagtctgtgcattaatttacttacgtatcacaccaaatacttattctgttgacgcattggttcaagtagaagatagtaaaggtgcagcttcagcagcattattaggtgaactatctaaaacggtaggcattgaacaaaaatctccagcagatgctgaaatacaaattcttagttcacgtatggtacttagtcaagttatacataacttaaatctagatatcacaattaaaaatcatgatgatactttcttcaaccgtcttttaaaccaagataaacagaatattgattataagaaagatgctgtaacttttagtaataaagatagttacttctctatccaacagcttcagattccttcgtattatcttgataaaccccttttactgtcttttaaagatcagaggcatttcactttcagctataaagataaagttatttttaatggtcagctcaatagtaataatttagtaactgctcgtgaaggacaatggaaagtacgaattaattctacacacgctccatctgtagagcagcaatttacaataagtaagtttgctctaccaactgcattacaaaaattcagttctacttacggcgtagctgaaaaaggtaaacaaacgggagttattgctttaaatttccaaggcactgataaagagcatatcactgaagtattaaacaatgtcttagccgtataccatagccagaatattga-

>KL24-FDAARGOS_123[LORJ01000003]

ttctcattgattgctcaatggaaactgattgtattatgtattcttataagtctagtctgtgcattaatttacttacgtatcacaccaaatacttattctgttgacgcattggttcaagtagaagatagtaaaggtgcagcttcagcagcattattaggtgaactatctaaaacggtaggcattgaacaaaaatctccagcagatgctgaaatacaaattcttagttcacgtatggtacttagtcaagttatacataacttaaatctagatatcacaattaaaaatcatgatgatactttcttcaaccgtcttttaaaccaagataaacagaatattgattataagaaagatgctgtaacttttagtaataaagatagttacttctctatccaacagcttcagattccttcgtattatcttgataaaccccttttactgtcttttaaagatcagaggcatttcactttcagctataaagataaagttatttttaatggtcagctcaatagtaataatttagtaactgctcgtgaaggacaatggaaagtacgaattaattctacacacgctccatctgtagagcagcaatttacaataagtaagtttgctctaccaactgcattacaaaaattcagttctacttacggcgtagctgaaaaaggtaaacaaacgggagttattgctttaaatttccaaggcactgataaagagcatatcactgaagtattaaacaatgtcttagccgtataccatagccagaatattga-

>KL24-M2[LAKP01000004]

ttctcattgattgctcaatggaaactgattgtattatgtattcttataagtctagtctgtgcattaatttacttacgtatcacaccaaatacttattctgttgacgcattggttcaagtagaagatagtaaaggtgcagcttcagcagcattattaggtgaactatctaaaacggtaggcattgaacaaaaatctccagcagatgctgaaatacaaattcttagttcacgtatggtacttagtcaagttatacataacttaaatctagatatcacaattaaaaatcatgatgatactttcttcaaccgtcttttaaaccaagataaacagaatattgattataagaaagatgctgtaacttttagtaataaagatagttacttctctatccaacagcttcagattccttcgtattatcttgataaaccccttttactgtcttttaaagatcagaggcatttcactttcagctataaagataaagttatttttaatggtcagctcaatagtaataatttagtaactgctcgtgaaggacaatggaaagtacgaattaattctacacacgctccatctgtagagcagcaatttacaataagtaagtttgctctaccaactgcattacaaaaattcagttctacttacggcgtagctgaaaaaggtaaacaaacgggagttattgctttaaatttccaaggcactgataaagagcatatcactgaagtattaaacaatgtcttagccgtataccatagccagaatattga-

>KL24-UMB002_[ AEPL01000017]

ttctcattgattgctcaatggaaactgattgtattatgtattcttataagtctagtctgtgcattaatttacttacgtatcacaccaaatacttattctgttgacgcattggttcaagtagaagatagtaaaggtgcagcttcagcagcattattaggtgaactatctaaaacggtaggcattgaacaaaaatctccagcagatgctgaaatacaaattcttagttcacgtatggtacttagtcaagttatacataacttaaatctagatatcacaattaaaaatcatgatgatactttcttcaaccgtcttttaaaccaagataaacagaatattgattataagaaagatgctgtaacttttagtaataaagatagttacttctctatccaacagcttcagattccttcgtattatcttgataaaccccttttactgtcttttaaagatcagaggcatttcactttcagctataaagataaagttatttttaatggtcagctcaatagtaataatttagtaactgctcgtgaaggacaatggaaagtacgaattaattctacacacgctccatctgtagagcagcaatttacaataagtaagtttgctctaccaactgcattacaaaaattcagttctacttacggcgtagctgaaaaaggtaaacaaacgggagttattgctttaaatttccaaggcactgataaagagcatatcactgaagtattaaacaatgtcttagccgtataccatagccagaatattga-

>KL27-4190_[ KT266827.1]

ttttcactcattgctcagtggaaactgattgcaatttgcatcattttaagccttgtttgtgcattgctttatttacgtgtaacaccagacacctactcagttgatgctttggttcaggttgaagacagtaaaggagcttctgcagcgcttttaggtgatttatcgaatatgattgagcaaaaatcacccgctcaagcagaaatagaaattttaaaatcacgtctggtgttgggttcagtcattaaagatttacatctaaatattcaaatttccagtactgaaaatacatttactcatcgtttattaagtgatacggaatataaaactgagtacaccaaaaaatcggttttatttaaagatggcctaaaaagttttgaagtacgtgagcttgaagtccctgcatattacttagataaaaaattacttcttaattttgataagcagtctttacgtttaactaaccctgaaaccgaagaagttatcttaactgttccattaaaccaagcgaaccatgttgcaggccctcatggtttatggaaaattgccatctttactaaagatcaatttgacgcgacctataatattaccaatttatctttaccagctgccgtaaatgcacttagtgcaaactatgctgtagcagaacgcggtaagcttacaggagttttaggtcttaactatcaagggcaagataaagaacatatcactaaagttttaaatgctattttagcgacttatagtgcccaaaatattga

>KL30-NIPH190_[ MN166189.1]

ttttcattgattgcacagtggaaaatcatagcatgctgcgtggtcttaagtttaatttgtgccttgctatacttacggataacaccagatacttattctgtagatgcaatggtacaagttgaggacagtaaaggtgcggcctctgctgctctacttggagatctttctaaagccagtggaggtctatctcaaaaatcaccagctgatccagaaatagagattttgaaatctcgtatggttttagggcaggtcatccagaatcttaaccttgatattaacattaaagataatcaatctagtttaattggtaaacttgtctcacaagaccaaagcaaactcgagtatcgtcatgatgctgtaatttacactaaccaaaacagtaatttaatagttaaacaactcagtgtgcctgaatattatttagacaaacctttaaaattagagtttaaagatgtaaatcaatttactctaacttataaagatcaagttgtatttaatggtttactaaacaagaaaaatgtactgaatacacaaaaagggttatggcaagttcagattaatacacatagaaacttgaaagatcacagctatacgctaactaagctcgctcttctaacagccgtaaatcaatttaattcaatttatagtgttgctgaaaaaggtaaaatgacaggtgttattggtttaagctacttaggtcaagatcctgagcatattactcaagtgctcaataatgttttaaatgtttatcatcaacaaaatatcga-

>KL37-NIPH146_[ APOU01000009]

ttttcattgattgcacagtggaaaatcatagcatgctgcgtggtcttaagtttaatttgtgccttgctatacttacggataacgccagatacttattctgtagatgcaatggtacaagttgaggacagtaaaggcgctgcctctgctgctttgcttggtgacctttctaaagtcagtggaggtctatctcaaaaatcaccagctgatccagaaatagagattcttcgttctagaatggttttaggacaagtcattcagaaccttaatcttgatattaacgtcaaagataaccaatcaggattgatcgacaagttaatttcacaagataagagtagacttgagtatcgtcatgaatctgtactgtacagtaatcaaaataataatttgattatacgagaacttaaagtacctgaatattatttagataaacccttaaagttagagttcaaaggtgcaaatcaattcactttaacatacaaagatcaagttgtatttaatggtcaattaaacaaaaaaaatatactaaatacaaatagaggtctttggcaggtacaacttaatgctcaaggtaacctgaaagaacaaagctatactttaactaaactcgctctaccaacagccgttaaaaattttaatgatatctatagtgttgctgaaaaaggtaaagtgactggtgttattggtttaaattatctaggtcaagatcctgagcacattacacaagtactaaataatgttctaactgtttatcaccaacaaaacattga-

>KL39-AB_2008-15-71_20_[ AMHP01000019.1]

ttttcactaattgctcagtggaaactagtcgcactctgcattattttaagccttgtatgtgcactactttatttacgtgtaacgccagatacctactcggttgatgctttggttcaagtcgaagacagcaaaggtgcttctgctgcacttttaggcgatttatcacaaatgattgagcaaaaatctcctgctcaagctgaaattgaaattctaaagtctcggttagttttaggttcggttattaaagaattacatttagatattcagatttctagcactgaaaataccttaactcaccgcttattaagtgatactgaatataaaactgaatacactaaaaattcagttttgtttaaagatggcactaaaaattttgatatacgaaaacttgaagttcctccatattatttagataaacagcttgtattaagctttgataaaaaatcttttaaattagtagatcctgagacagatgaggttgtactcacagctccactcaatcaaagtagtcaaattacaggagcctatggtgtatggaaggtggctatctttactaaagatttattagattcaaaatataatattaaaaaattgtctattcccgcagctgtagataatataagttcaaattattcagtagctgaacgcggtaaattaacaggtattcttggtttaaattatcaaggacatgacaaagaacatattactcaagtcttaaatgcgatcttagtgacatatggcgcccaaaatgttga

>KL44-NIPH_70_[ MN148385.1]

ttttcattgattgctcagtggaaactcattgctctgtgcgttattttaagcgttgtatgtaccctactctatttacgtgtaacgccagatacctattcggtagatgctttggttcaggttgaggacagtaaaggggcttctgcagcgcttttaggtgatctatcacaaatgatcgagcaaaaatcaccagctcaagcagaaatagaaattttaaaatcacgtctagttttaggttctgttattaaggatttacatctgaatatacaggtctctagcactgaaaatacactgactcatcgcttattaagtgatactgaatataaaactgaatacactaagaaatcagttttatttaaagataacttaaaaagttttgaagtgcgtgaatttgaagttccagcttactatctagacaaaaacttacttcttaattttgataaacagtctttacgtttaacagacccagatactgaagaagttatcttaactgttccattaaaccaagctaatcacgttgcaggtcctcatggtttatggaaaattgccatctttactaaagatcaatttgacgcgacctataatattaccaatttatctttaccagctgctgtaaatgcacttagtgcaaattattcagtagcagaacgcggtaagcttacaggagttttaggtcttaactatcaagggcaagataaagaacacattaccaaagttcttaatgcgattttagctacctatagtgcccaaaatattga

>KL45-NIPH201_[ MN166190.1]

ttttcattgattgcccaatggaaattgattgtattatgtattctcataagtttagtctgtgcattaatttatttacgtgtcacgcccaatacttattcagttgatgcattggttcaggtcgaagatactaaaagtgcagcttcagcagctttactaggtgaactatctaaaatggttgaccaaaagtctcctgctcaggcagaaatagaagttcttacttcacgtatggtacttggtcaagttatcaacaacttaaatcttgatattacaattaaaaatcacgatgatacttttttcaatcgtcttttaagtcaagataaacagaatattgattataagaaagatgctgtaacttttagtaataaagatagttatttctctatccaacagcttcagattccttcgtattatcttgataaaccgcttttactttcttttaaagatcagaggcatttcactttcagctataaagataaagttatttttagtggtcagctcaatagtaataatttagtaactgctcgtgaaggacaatggaaagtacgaattaattctacacacgctccatctgtagagcagcaatttactatcagtaaacttgctttaccaactgccgtacaaaaacttggctctacttacggggtagctgaacgaggcaaacaaacgggagttatcagcttaaattatcagggaacagataaagaacatatcactgaagtcttaaataatattttagctgtatatcatagccaaaatattga

>KL47-NIPH601_[ APQZ01000009]

ttttcattgattgcccaatggaaattgattgtactatgtattcttataagtctagtctgtgcattaatttatttacgtgtcactccaaatacttattcggttgatgcattggttcaggtcgaagatactaaaagtgcagcttcagcagctttattaggtgaactatctaaaatggttgaccaaaaatctccagcagaggctgaaatacaggttcttacctctcgtatggtgctcggtcaggttatccacaatttaaatctagatatcacaattaaaaatcatgatgatactttcttcaaccgtcttttaagtcaagataaacagaatattgattataagaaagatgctgtaacttttagtaataaagatagttatttctctatccaacagtttcagattccttcgtattatcttgataaaccccttttactttcttttaaagatcaggggcatttcactttcagctataaagataaagttatttttagtggtcagctcaatagtaataatttagtaactgctcgtgaaggacaatggaaagtacgaattaattctacacacgctccatctgtagagcagcagtttacaatcagtaaacttgctttaccaactgccgtacaaaaactcggctctacttacggggtagctgaacgaggcaaacaaacgggagttatcggattaagttttcaaggaactgataaagaacacatcactgaagtcttaaataatattttagctgtatatcatagccaaaatattga

>KL47-UV1043_[ KX661320.1]

ttttcattgattgcccaatggaaattgattgtactatgtattcttataagtctagtctgtgcattaatttatttacgtgtcactccaaatacttattcggttgatgcattggttcaggtcgaagatactaaaagtgcagcttcagcagctttattaggtgaactatctaaaatggttgaccaaaaatctccagcagaggctgaaatacaggttcttacctctcgtatggtgctcggtcaggttatccacaatttaaatctagatatcacaattaaaaatcatgatgatactttcttcaaccgtcttttaagtcaagataaacagaatattgattataagaaagatgctgtaacttttagtaataaagatagttatttctctatccaacagtttcagattccttcgtattatcttgataaaccccttttactttcttttaaagatcaggggcatttcactttcagctataaagataaagttatttttagtggtcagctcaatagtaataatttagtaactgctcgtgaaggacaatggaaagtacgaattaattctacacacgctccatctgtagagcagcagtttacaatcagtaaacttgctttaccaactgccgtacaaaaactcggctctacttacggggtagctgaacgaggcaaacaaacgggagttatcggattaagttttcaaggaactgataaagaacacatcactgaagtcttaaataatattttagctgtatatcatagccaaaatattga

>KL48-NIPH615_[ MN166191.1]

ttttctttgattgcacagtggaaaatcatagcatgctgtgtgattttaagtttaatttgtgccttattatatttacggataacaccagatatttatgctgtagatgcaatggtacaagtggaagatagtaaaggtgctgcctctgctgctttgcttggtgacctttctaaagtcagtggaggtctatctcaaaaatcaccagctgatccagaaatagagattcttcgttctagaatggttttaggacaagtcatccagaaccttaatcttgatattaacgtcaaagataaccaatcaggattgatcgacaagttaatttcacaagataagagtagacttgagtatcgtcatgaatctgtactgtacagtaatcaaaataataatttgattatacgagaacttaaagtacctgaatattatttagataaacccttaaagttagagttcaaaggtgcaaatcaattcactttaacatacaaagatcaagttgtatttaatggtcaattaaacaaaaaaaatatactaaatacaaatagaggtctttggcaggtacaacttaatgctcaaggtaacctgaaagaacaaagctatactttaactaaactcgctctaccaacagccgttaaaaattttaatgatatctatagtgttgctgaaaaaggtaaagtgactggtgttattggtttaaattatctaggtcaagatcctgagcacattacacaagtactaaataatgttctaactgtttatcaccaacaaaacattga-

>KL49-NIPH1734_[ NZ_KB849325]

ttctcattgattacacagtggaaattaattgcactctgcaccattttaagtcttgtatgcgctctactctatttacgtgtaacaccaaatacctattcggtggatgctttggttcaggttgaagacagtaaaggcgcctctgctgcactcttaggtgatttatcacaaatgattgagcaaaaatcacctgctcaagcagagattgaaattttaaaatctcgtttggttttaggctcagtcattaaagatctacatttaaatgtacaaatttccagtactgaaaacactttaactcaccgtttattaagtggaacggaatataaaactgaatacacacaaaaatcagttatttttaaagatggcctaaaaagtttcgaagttcgtgaattcgaaattcctgcttattatttagataaaactttacatcttaattttgacaagcaatctttacgtttaacagatccaaagactgaagaagttatcttaactgtgccattaaaccaagttaaccgtgttgcaggccctcatggtttatggaaagtcgccatctttacaaaagaacaatttgacgcgacctataatattaccaatttgtccttaccggctaccgtaagtgccattagttcaaactactcagtcgcagagcgaggtaagctcactggtgtattaagtttaagctatcaaggtcaagataaagagtatattactaaagttttaaatgctattttatcaacttatagtgcacaaaatattga

>KL49-BAL_173_[ KT359616.1]

ttctcattgattacacagtggaaattaattgcactctgcaccattttaagtcttgtatgcgctctactctatttacgtgtaacaccaaatacctattcggtggatgctttggttcaggttgaagacagtaaaggcgcctctgctgcactcttaggtgatttatcacaaatgattgagcaaaaatcacctgctcaagcagagattgaaattttaaaatctcgtttggttttaggctcagtcattaaagatctacatttaaatgtacaaatttccagtactgaaaacactttaactcaccgtttattaagtggaacggaatataaaactgaatacacacaaaaatcagttatttttaaagatggcctaaaaagtttcgaagttcgtgaattcgaaattcctgcttattatttagataaaactttacatcttaattttgacaagcaatctttacgtttaacagatccaaagactgaagaagttatcttaactgtgccattaaaccaagttaaccgtgttgcaggccctcatggtttatggaaagtcgccatctttacaaaagaacaatttgacgcgacctataatattaccaatttgtccttaccggctaccgtaagtgccattagttcaaactactcagtcgcagagcgaggtaagctcactggtgtattaagtttaagctatcaaggtcaagataaagagtatattactaaagttttaaatgctattttatcaacttatagtgcacaaaatattga

>KL49-NIPH335_[ NZ_KB849886]

ttctcattgattacacagtggaaattaattgcactctgcaccattttaagtcttgtatgcgctctactctatttacgtgtaacaccaaatacctattcggtggatgctttggttcaggttgaagacagtaaaggcgcctctgctgcactcttaggtgatttatcacaaatgattgagcaaaaatcacctgctcaagcagagattgaaattttaaaatctcgtttggttttaggctcagtcattaaagatctacatttaaatgtacaaatttccagtactgaaaacactttaactcaccgtttattaagtggaacggaatataaaactgaatacacacaaaaatcagttatttttaaagatggcctaaaaagtttcgaagttcgtgaattcgaaattcctgcttattatttagataaaactttacatcttaattttgacaagcaatctttacgtttaacagatccaaagactgaagaagttatcttaactgtgccattaaaccaagttaaccgtgttgcaggccctcatggtttatggaaagtcgccatctttacaaaagaacaatttgacgcgacctataatattaccaatttgtccttaccggctaccgtaagtgccattagttcaaactactcagtcgcagagcgaggtaagctcactggtgtattaagtttaagctatcaaggtcaagataaagagtatattactaaagttttaaatgctattttatcaacttatagtgcacaaaatattga

>KL49-LAC4_[ JICJ01000028]

ttctcattgattacacagtggaaattaattgcactctgcaccattttaagtcttgtatgcgctctactctatttacgtgtaacaccaaatacctattcggtggatgctttggttcaggttgaagacagtaaaggcgcctctgctgcactcttaggtgatttatcacaaatgattgagcaaaaatcacctgctcaagcagagattgaaattttaaaatctcgtttggttttaggctcagtcattaaagatctacatttaaatgtacaaatttccagtactgaaaacactttaactcaccgtttattaagtggaacggaatataaaactgaatacacacaaaaatcagttatttttaaagatggcctaaaaagtttcgaagttcgtgaattcgaaattcctgcttattatttagataaaactttacatcttaattttgacaagcaatctttacgtttaacagatccaaagactgaagaagttatcttaactgtgccattaaaccaagttaaccgtgttgcaggccctcatggtttatggaaagtcgccatctttacaaaagaacaatttgacgcgacctataatattaccaatttgtccttaccggctaccgtaagtgccattagttcaaactactcagtcgcagagcgaggtaagctcactggtgtattaagtttaagctatcaaggtcaagataaagagtatattactaaagttttaaatgctattttatcaacttatagtgcacaaaatattga

>KL57-BAL_212_[ KY434631.1]

ttttcgttgattgctcagtggaaaatcatagcatgctgcgtggtcttaagtttaatttgtgccttgctatacttacgtataacaacagatacttattctgtagatgcaatggtacaagttgaagacagtaaaggtgcggcctctgctgctctacttggagatctttctaaagtaagtggaggtctatctcaaaaatcacctgctgatccagaaatagagattttgaaatctcgtatggttctagggcaagttatccaaaatcttaatcttgatattaatatcaaagataaccaatcaggattaatcaataagttaatatcaccagaccaaagtaaactcgagtatcatcatgaagctgtaacctacactaaccataatgatactttaatagttcgccaactaaaggtgcctgaatattatttagataaacctttaaaattagattttaaaagtacaaatcaatttactctaacctataaagatgaagttgtatttaatggtctgctaaaccaaaaaaatagtctaaatacaaacaaaggtctttggcaggtacaacttaacacccaaggtaatctgaaagatcatagttacactttaactaagctcgctctactaacagccgtaaatcaatttaattcaatttatagtgttgctgaaaaaggtaagatgacaggtgttattggtttaagctatttaggtcaagatccagagcatattacacaagtgctcaataacgttctaaatgtttatcatcaacaaaatatcga-

>KL58-BAL_114_[ KT359617.1]

ttttcattgattgctcagtggaaactcattgctctatgcgttattttaagcgttgtatgtgccctactctatttacgtgtaacgccagatacctattcggtagatgctttggttcaggttgaagacagtaaaggggcttctgctgcacttttaggtgatctctcacaaatgattgagcaaaaatcaccagctcaagcagaaatagaaattttaaaatctcgtttggttttaggttctgtgattaaagatttacatctgaatatacaggtctctagcactgagaatacatttacacatcgcttattgagtgatactgattataaaactgaatatgctaaaaaatcggttttatttaaagatggtttaaaaagttttgatatacgtcagtttgagattccagcttattatctagataagaacttacttcttgactttgataaacagtctttacgtttaacagacccagatactgaagaagtcatcttaactgttccattaaaccaagctaacagcgttacaggaccgtatggtttatggaaagttgctatatttacaaaagaccaatttgactcaacttataatattaagaaattatcacttcctttagctataaaatctatcagttccgactattctgtagaagaaaaaggtaaactcacaggtatattaggacttagctatcaaggccaagatcaagaacatattactaaagtcttaaatgctattttagcgacttatagtgcacaaaatattga

>KL73-SGH0703_[ MF362178.1]

ttttcactgattgcacagtggaagcttatcgctctatgcgttattttaagcctagtatgtgctctattgtacttacgggtaacccctgatacttattcagtggatgcactggttcaagttgaagacagtaaaggggcttctgctgcgcttttaggtgacttatcacaaatgattgagcaaaaatcaccagctcaagcagaaattgagattttaaaatctcgtttagttttaggttcagtcattaaagacttacatctcaacatacaagtttctagtaccgaaaatacatttactcatcgtttattgagtaatccagaatataaaactgaatatactaagaaatctgttatttttaaggatggattaaaaagttttgatatccatcagtttgatataccagcttattacttagataaaaatttatttcttgattttgataaacaatctttccgtttaacagacccaaagactgaagaagttatcttaactcttccacttaatcagttaaaccaagttactggcccacatgggacgtggaaagttgctatctttacaaaagaccaatttgatgccacttataatattagaaatctatctttacctgctgctgtaaattcaattagttcaaactactcagttgctgaacgtggtaaattaacgggtattctaggtttaaattatcagggtcaagatcaagaacatattactaaggttttaaacgcaattttagctacttacagtgcacaaaatattga

>KL91-1053_[ KM402814.1]

ttttcactcattgcacagtggaagttaatcgtactctgcattattttaagccttatatgtgcactactttatttacgtgtaacgccagatacctactcggttgatgctttggttcaagtcgaagacagcaaaggtgcttctgctgcgcttttaggcgatttgtcagatatgattgagcaaaaatctcctgctcaagctgaaatcgagattttaaaatctcgtttggttttgggctcagtcattaaagatttacatcttaacatacaagtttctagcactgaaaatacacttactcatcgcttattaagtgatactgattataaaactgaatatacaaaaaactctgtgatatttaaggacggtttaaagagctttgaaattcgccagtttgaaattccagcttactatctcgataaaaccttacaacttaactttaataaacagtctttacgtttaactgacccagcgacagaacaaactcttttaacggtaccccttaatcagctaaatcaagtcacaggtccacaaggaatttggaaagttagtatttttactaaagataaacttgatacaacctataatattactagcttatctttaccagcagcagtagatactattggttcaaactattctgtagctgagagaggcaagcttacaggtgtgttaggtcttaactatcaaggtcaagacaaagagcatatcactaaagttttaaatgctattttagcaacttatagtgcgcaaaatattga

>KL93-B11911_[ BK010902]

Ttttcactgattgcacagtggaaactcattgctctatgcgttattttaagcgttgtatgtgcactactctatttacgtgtaacaccagatacctattcggtagatgctttagttcaggttgaggacagcaagggagcttctgctgcacttttaggcgatctatcacaaatgatcgagcaaaaatcaccagctcaagcagaaatagaaattttaaaatctcgtttggttttaggttctgttattaaagatttacatctgaatatacaggtctctagcactgaaaatacatttacacatcgcttattgagtgatactgattacaaaactgaatatgctaaaaaatcggttttatttaaagatggtttaaaaagttttgatatacgtcagtttgagattccagcttattacttagataagaacttacttcttgattttgataaacagtctttacgtttaacagatccagatactgaagaagtcatcttaactgttccattaaaccaagctaacagcgttacaggaccgtatggtgtatggaaagttgctatatttacaaacgaccaatttgaatcaacttataatattaagaaattatcacttcctttagctataaaatctatcagttccgactattctgtagaagaaaaaggtaaactcacaggtatattaggacttagctatcaagggcaagatcaagaacatattactaaagtcttaaatgctattttagcgacctatagtgcacaaaatattga

>KL102_KZ1102 [MK399429]

ttttcattgattgcccaatggaaattgattgtactatgtattcttataagtctagtctgtgcattaatttatttacgtgtcactccaaatacttattcggttgatgcattggttcaggtcgaagatactaaaagtgcagcttcagcagctttattaggtgaactatctaaaatggttgaccaaaaatctccagcagaggctgaaatacaggttcttacctctcgtatggtgctcggtcaggttatccacaatttaaatctagatatcacaattaaaaatcatgatgatactttcttcaaccgtcttttaagtcaagataaacagaatattgattataagaaagatgctgtaacttttagtaataaagatagttatttctctatccaacagtttcagattccttcgtattatcttgataaaccccttttactttcttttaaagatcaggggcatttcactttcagctataaagataaagttatttttagtggtcagctcaatagtaataatttagtaactgctcgtgaaggacaatggaaagtacgaattaattctacacacgctccatctgtagagcagcagtttacaatcagtaaacttgctttaccaactgccgtacaaaaactcggctctacttacggggtagctgaacgaggcaaacaaacgggagttatcggattaagttttcaaggaactgataaagaacatatcactgaagtcttaaataatattttagctgtatatcatagccaaaatattga

>KL105-625974_[ JEXD01000015.1]

ttttcattgattgcacagtggaaaatcatagcatgctgcgtgatcttaagtttaatttgtgccttgttatatttacgcataacaacagatacttattctgtagatgctatggtacaagttgaagacagtaaaggcgctgcctctgctgctttattgggagacctttctaaagttactggaggtatatctcaaaaatcaccagctgatccagaaatagagattttaaaatctcgtatggttttagggcaagtgattcataaccttaatcttgatattaaaattaaagataatcaatcaggattgattgataagttaatttcacaagataagagtaaaattgaatatcgtcatgaatcggtagtttacaataacttaaacgctagtttgattattcaagaatttaaggtgcctgaatattattttgataaacctttgaaattagagtttaaagatacaaatcaatttactctaatgtataaagatcaagttgtatttaatggtcaattaaacaagaaaaatatattaaattcaaataaaggtttatggcaagttcaaatcaatgcacaaggcaatttaaaagatcaaagttatactttaactaaattagctcttttaacagctgtaaatcaatttaattcaatatattcagttaatgaaaaaggtaaaatgaccggagttatcggtttaagctatttaggccaagatcctgaacatattactcaagtgctcaataatgttctaaatgtttatcatgaacaaaatattga-

>KL106-219_ABAU_[ JVPN01000008.1]

ttttcattgattgcacagtggaaaatcatagcatgctgcgtggtcttaagtttaatttgtgccttgctatatttacgcataacaacagatacttattctgtagatgctatggtacaagttgaagacagtaaaggcgctgcctctgctgctttattgggagacctttctaaagttactggagctatatctcaaaaatcaccagctgatccagaaatagagattttaaaatctcgtatggttttaggacaagttattcagaatcttaatcttgatattaagattaaagataatcaatcaggattgattgataagttaatttcacaagataagagtaaaattgaatatcgtcatgaatctgtactttacaaaaatttaaataatagtttaattattcgagaacttaaagtacctgaatattatttagataaacctttaaaattagagtttaaaggtataaatcaatttactttagcttataaagatcaaattgtatttaatggtcaactaaacaagaaaaatacattaaattcaaataaaggtttatggcaagttcaaattaatacacaaggtaatttaaaagatcaaagctatacattaactaaattggcccttttaacagctgtaaatcaatttaattcaatctatagtgttgctgaaaaaggtaaaatgactggagttattggtttaagctatttaggtcaagatccagagcacattactcaagtactcaataacgttttaaatgtttatcataaacaaaacattga-

>KL106-TG22198_[ NZ_ASFT01000013]

ttttcattgattgcacagtggaaaatcatagcatgctgcgtggtcttaagtttaatttgtgccttgctatatttacgcataacaacagatacttattctgtagatgctatggtacaagttgaagacagtaaaggcgctgcctctgctgctttattgggagacctttctaaagttactggagctatatctcaaaaatcaccagctgatccagaaatagagattttaaaatctcgtatggttttaggacaagttattcagaatcttaatcttgatattaagattaaagataatcaatctggattgattgataagttaatttcacaagataagagtaaaattgaatatcgtcatgaatctgtactttacaaaaatttaaataatagtttaattattcgagaacttaaagtacctgaatattatttagataaacctttaaaattagagtttaaaggtacaaatcaatttactttagcttataaagatcaaattgtatttaatggtcaactaaacaagaaaaatacattaaattcaaataaaggattatggcaagttcaaattaatacacaaggtaatttaaaagatcaaagctatacattaactaaattggcccttttaacagctgtaaatcaatttaattcaatctatagtgttgctgaaaaaggtaaaatgactggagttattggtttaagctatttaggtcaagatccagagcacattactcaagtactcaataacgttttaaatgtttatcataaacaaaacattga-

>KL107_MSHR_183[MK370022.1]

ttttcactgattgcacagtggaaactaatcgcactctgcattattttaagccttgtatgcgcactactttatttacgtgtaacaccagatacctactcggttgatgctttggttcaagttgaagacagcaaaggtgcttctgctgcacttttgggcgatttgtccgatatgatcgaacaaaagtcaccagcgcaagctgaaattgaaattttaaagtctcggttggttttaggctcggttattaaagaactacatctagatattcacgtttctagcaccgaaaacacacttactcaccgtttattaagtgatactgaatataaaactgaatacactaaaaattcagttttatttaaagatggcaccaaaaattttgatatacgaagatttgaagtacctgcatattatttagacaaacaacttgtattaagctttgatcaaaaatcttttaaattagtagatcctgagacggatgaagttgtactcacagctccaatcaatcaaaataaccaaattacaggagcttatggcgtatggaaggttgcgatctttactaaagatcaattagattcaaaatataatattaagaaactctctattccagcagctgtagataatataagttcaaattattcagtagccgaacgcggtaaattaacaggtattcttggtttaaattatcaaggatatgataaagaacatattactcaagtcttaaatgcgatcttggtaacatatggtgcccaaaatgttga

**The RECORD statement – checklist of items, extended from the STROBE statement, that should be reported in observational studies using routinely collected health data.**

|  | **Item No.** | **STROBE items** | **Location in manuscript where items are reported** | **RECORD items** | **Location in manuscript where items are reported** |
| --- | --- | --- | --- | --- | --- |
| **Title and abstract** | | | | | |
|  | 1 | (a) Indicate the study’s design with a commonly used term in the title or the abstract (b) Provide in the abstract an informative and balanced summary of what was done and what was found |  | RECORD 1.1: The type of data used should be specified in the title or abstract. When possible, the name of the databases used should be included.  RECORD 1.2: If applicable, the geographic region and timeframe within which the study took place should be reported in the title or abstract.  RECORD 1.3: If linkage between databases was conducted for the study, this should be clearly stated in the title or abstract. | P.3-4 |
| **Introduction** | | | | | |
| Background rationale | 2 | Explain the scientific background and rationale for the investigation being reported |  |  | P.5-6 |
| Objectives | 3 | State specific objectives, including any prespecified hypotheses |  |  | P.6 |
| **Methods** | | | | | |
| Study Design | 4 | Present key elements of study design early in the paper |  |  | P.7-8 |
| Setting | 5 | Describe the setting, locations, and relevant dates, including periods of recruitment, exposure, follow-up, and data collection |  |  | P.7-8, 11-12 |
| Participants | 6 | *(a) Cohort study* - Give the eligibility criteria, and the sources and methods of selection of participants. Describe methods of follow-up  *Case-control study* - Give the eligibility criteria, and the sources and methods of case ascertainment and control selection. Give the rationale for the choice of cases and controls  *Cross-sectional study* - Give the eligibility criteria, and the sources and methods of selection of participants  *(b) Cohort study* - For matched studies, give matching criteria and number of exposed and unexposed  *Case-control study* - For matched studies, give matching criteria and the number of controls per case |  | RECORD 6.1: The methods of study population selection (such as codes or algorithms used to identify subjects) should be listed in detail. If this is not possible, an explanation should be provided.  RECORD 6.2: Any validation studies of the codes or algorithms used to select the population should be referenced. If validation was conducted for this study and not published elsewhere, detailed methods and results should be provided.  RECORD 6.3: If the study involved linkage of databases, consider use of a flow diagram or other graphical display to demonstrate the data linkage process, including the number of individuals with linked data at each stage. | P.7-8 |
| Variables | 7 | Clearly define all outcomes, exposures, predictors, potential confounders, and effect modifiers. Give diagnostic criteria, if applicable. |  | RECORD 7.1: A complete list of codes and algorithms used to classify exposures, outcomes, confounders, and effect modifiers should be provided. If these cannot be reported, an explanation should be provided. | P.11-12 |
| Data sources/ measurement | 8 | For each variable of interest, give sources of data and details of methods of assessment (measurement).  Describe comparability of assessment methods if there is more than one group |  |  | P.7-12 |
| Bias | 9 | Describe any efforts to address potential sources of bias |  |  | P.7-8, 11-12 |
| Study size | 10 | Explain how the study size was arrived at |  |  | P.7-8 |
| Quantitative variables | 11 | Explain how quantitative variables were handled in the analyses. If applicable, describe which groupings were chosen, and why |  |  | P.11-12 |
| Statistical methods | 12 | (a) Describe all statistical methods, including those used to control for confounding  (b) Describe any methods used to examine subgroups and interactions  (c) Explain how missing data were addressed  (d) *Cohort study* - If applicable, explain how loss to follow-up was addressed  *Case-control study* - If applicable, explain how matching of cases and controls was addressed  *Cross-sectional study* - If applicable, describe analytical methods taking account of sampling strategy  (e) Describe any sensitivity analyses |  |  | P.12-13 |
| Data access and cleaning methods |  | .. |  | RECORD 12.1: Authors should describe the extent to which the investigators had access to the database population used to create the study population.  RECORD 12.2: Authors should provide information on the data cleaning methods used in the study. | P.7-8 |
| Linkage |  | .. |  | RECORD 12.3: State whether the study included person-level, institutional-level, or other data linkage across two or more databases. The methods of linkage and methods of linkage quality evaluation should be provided. | P.7-8, 11-12 |
| **Results** | | | | | |
| Participants | 13 | (a) Report the numbers of individuals at each stage of the study (*e.g.*, numbers potentially eligible, examined for eligibility, confirmed eligible, included in the study, completing follow-up, and analysed)  (b) Give reasons for non-participation at each stage.  (c) Consider use of a flow diagram |  | RECORD 13.1: Describe in detail the selection of the persons included in the study (*i.e.,* study population selection) including filtering based on data quality, data availability and linkage. The selection of included persons can be described in the text and/or by means of the study flow diagram. | P.13 |
| Descriptive data | 14 | (a) Give characteristics of study participants (*e.g.*, demographic, clinical, social) and information on exposures and potential confounders  (b) Indicate the number of participants with missing data for each variable of interest  (c) *Cohort study* - summarise follow-up time (*e.g.*, average and total amount) |  |  | P.13 |
| Outcome data | 15 | *Cohort study* - Report numbers of outcome events or summary measures over time  *Case-control study* - Report numbers in each exposure category, or summary measures of exposure  *Cross-sectional study* - Report numbers of outcome events or summary measures |  |  | P.13-17 |
| Main results | 16 | (a) Give unadjusted estimates and, if applicable, confounder-adjusted estimates and their precision (e.g., 95% confidence interval). Make clear which confounders were adjusted for and why they were included  (b) Report category boundaries when continuous variables were categorized  (c) If relevant, consider translating estimates of relative risk into absolute risk for a meaningful time period |  |  | P.13-17 |
| Other analyses | 17 | Report other analyses done—e.g., analyses of subgroups and interactions, and sensitivity analyses |  |  | P.13-17 |
| **Discussion** | | | | | |
| Key results | 18 | Summarise key results with reference to study objectives |  |  | P.20 |
| Limitations | 19 | Discuss limitations of the study, taking into account sources of potential bias or imprecision. Discuss both direction and magnitude of any potential bias |  | RECORD 19.1: Discuss the implications of using data that were not created or collected to answer the specific research question(s). Include discussion of misclassification bias, unmeasured confounding, missing data, and changing eligibility over time, as they pertain to the study being reported. | P.19, 20-21, 22 |
| Interpretation | 20 | Give a cautious overall interpretation of results considering objectives, limitations, multiplicity of analyses, results from similar studies, and other relevant evidence |  |  | P.18-23 |
| Generalisability | 21 | Discuss the generalisability (external validity) of the study results |  |  | P.19-21 |
| **Other Information** | | | | | |
| Funding | 22 | Give the source of funding and the role of the funders for the present study and, if applicable, for the original study on which the present article is based |  |  | P.23-24 |
| Accessibility of protocol, raw data, and programming code |  | .. |  | RECORD 22.1: Authors should provide information on how to access any supplemental information such as the study protocol, raw data, or programming code. | P.8-11 |

*Reference: Benchimol EI, Smeeth L, Guttmann A, Harron K, Moher D, Petersen I, Sørensen HT, von Elm E, Langan SM, the RECORD Working Committee. The REporting of studies Conducted using Observational Routinely-collected health Data (RECORD) Statement. *PLoS Medicine* 2015; in press.

*Checklist is protected under Creative Commons Attribution ([CC BY](http://creativecommons.org/licenses/by/4.0/)) license.
